# Phosphonium fullerides: isolable zwitterionic adducts of a phosphine with C_60_†

**DOI:** 10.1039/d5sc00367a

**Published:** 2025-06-26

**Authors:** Maike B. Röthel, Jonas H. Franzen, Daniel Leitner, Thomas S. Hofer, Michael Seidl, Fabian Dielmann

**Affiliations:** a Institute of General, Inorganic and Theoretical Chemistry, Universität Innsbruck Innrain 80-82 6020 Innsbruck Austria fabian.dielmann@uibk.ac.at

## Abstract

Although fullerene derivatization has been extensively studied for decades, zwitterionic adducts with neutral Lewis bases are rare, and those with tertiary phosphines remain elusive. This work presents a combined experimental and computational study on the first isolable zwitterionic phosphonium fullerides. The reaction of tris(tetramethylguanidinyl)phosphine ((tmg)_3_P) with C_60_ results in the formation of the zwitterionic adducts (tmg)_3_PC_60_ and (tmg)_3_PC_60_P(tmg)_3_ in quantitative yield. Stoichiometric studies demonstrate that up to two (tmg)_3_P molecules can reversibly bind to C_60_, forming bisphosphine adducts as regioisomeric mixtures with reduced P–C bond stability. Spectroscopic, crystallographic, and computational analyses reveal the presence of σ-type dative P–C bonds and significant charge redistribution within the fullerene cage. Furthermore, functionalizations of the phosphonium fullerides with electrophiles yield ionic derivatives, highlighting their reactivity and potential for further modification.

## Abstract 2

The reaction of phosphine (tmg)_3_P with C_60_ leads to isolable zwitterionic phosphonium fullerides with reversible P–C dative bonds and increased negative charge on the C_60_ core, enabling further electrophilic functionalization.

## Graphical abstract

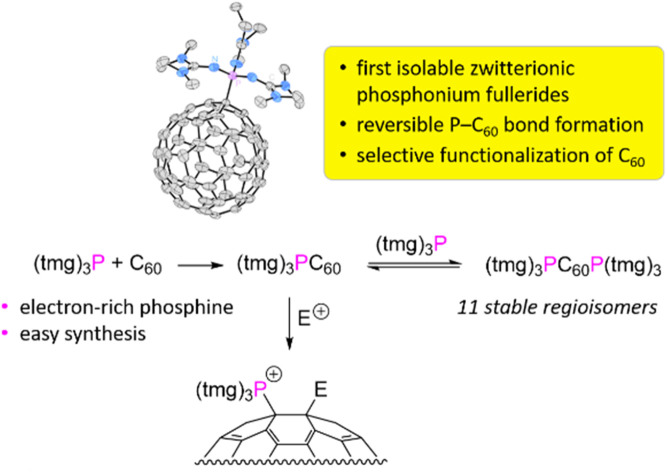

## Introduction

**Fig. 1:**
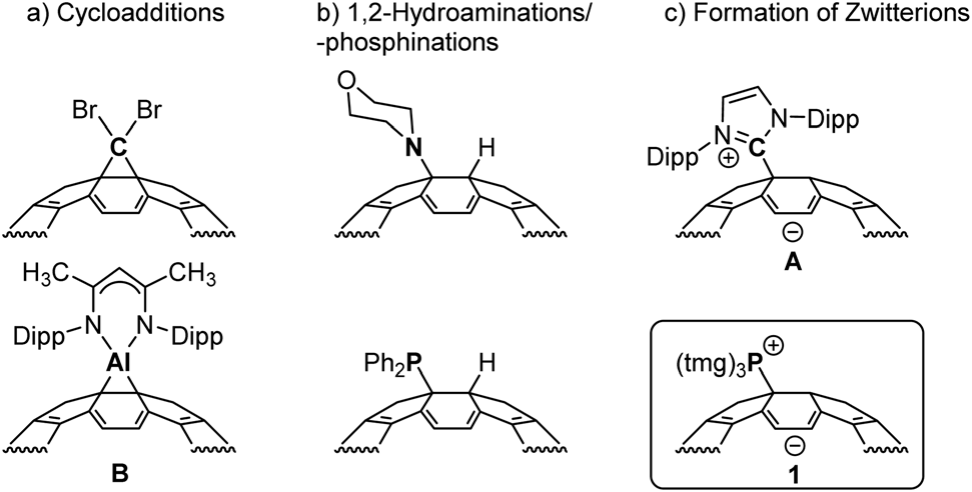
Selected fullerene derivatives obtained by reaction of buckminsterfullerene with neutral Lewis bases *via* (a) cycloadditions,^10,22^ (b) 1,2-hydroaminations^12^ or 1,2-hydrophosphinations,^13^ (c) formation of zwitterionic adducts^15^ including the herein presented phosphonium fulleride (Dipp = 2,6-diisopropylphenyl, tmg = tetramethylguanidinyl).

With the development of a multigram preparation method for buckminsterfullerene (C_60_)^1^ and its ton-scale production,^2,3^ research into fullerene derivatization has intensified.^3,4^ A particular focus lies in the selective functionalization of the C_60_ cage, aiming to achieve unique structural and electronic properties with multifaceted application potential for high-tech, fullerene-based materials.^5,6^ Due to its pronounced electrophilicity,^7^ C_60_ reacts preferentially with nucleophilic reagents and accepts electrons from strong electron donors. In this context the reactivity with various neutral Lewis bases was investigated. A common reaction pathway of ambiphilic reagents is the cycloaddition at the C–C bond bisecting two six-membered rings of the C_60_ cage, as observed with carbenes^8^ and their heavier homologues^9,10^ (Fig. 1a). Primary and secondary amines undergo hydroamination,^11,12^ analogous to secondary phosphines, reacting to form 1,2-hydrophosphination products^13,14^ (Fig. 1b). While such 1,2-additions with neutral nucleophiles are widely documented, there are few examples where the nucleophile binds to a single carbon atom, forming a zwitterionic adduct. For example, the zwitterionic product A has been isolated from the reaction of the *N*-heterocyclic carbene (NHC) 1,3-bis(2,6-diisopropylphenyl)imidazolin-2-ylidene with C_60_ (Fig. 1c).^15–17^ In the reaction between the non-nucleophilic base 1,8-diazabicyclo[5.4.0]undec-7-ene (DBU) and C_60_, initial electron transfer forms [DBU]^+^[C_60_]^−^, and spectroscopic data suggest subsequent formation of the zwitterionic adduct.^18^ Recent studies by Hobza and coworkers provide evidence for the formation of dative piperidine N–C_60_ bonds, which is enhanced cooperatively *via* hydrogen bonding interactions between the piperidine molecules.^19^ To date, however, isolable zwitterionic Lewis base adducts with tertiary amines or tertiary phosphines remain elusive.^20,21^

Regarding tertiary phosphines, computational studies by Hobza and coworkers have shown that the substituents at the phosphorus atom significantly influence the stability of the resulting C_60_–P bond.^23^ Specifically, π-donating nitrogen substituents were found to enhance dative C_60_–P interaction, suggesting near thermoneutral C_60_ complexation when phosphines bear dimethylamino or 1-pyrrolidinyl substituents. Our group has further demonstrated that phosphines with strongly π-donating guanidine-type substituents exhibit superbasic character^24^ and act as strong nucleophiles towards various substrates, including carbon dioxide,^25^ chlorazolium salts,^26^ sulfur dioxide^27^ and sulfur hexafluoride.^28^ In this study we investigate the complexation reaction of tris(tetramethylguanidinyl)phosphine^29^ – a synthetically easily accessible phosphorus superbase – with buckminsterfullerene.

## Results and discussion

### Synthesis and characterization of tris(tetramethylguanidinyl)phosphonium fulleride (1)

**Fig. 2:**
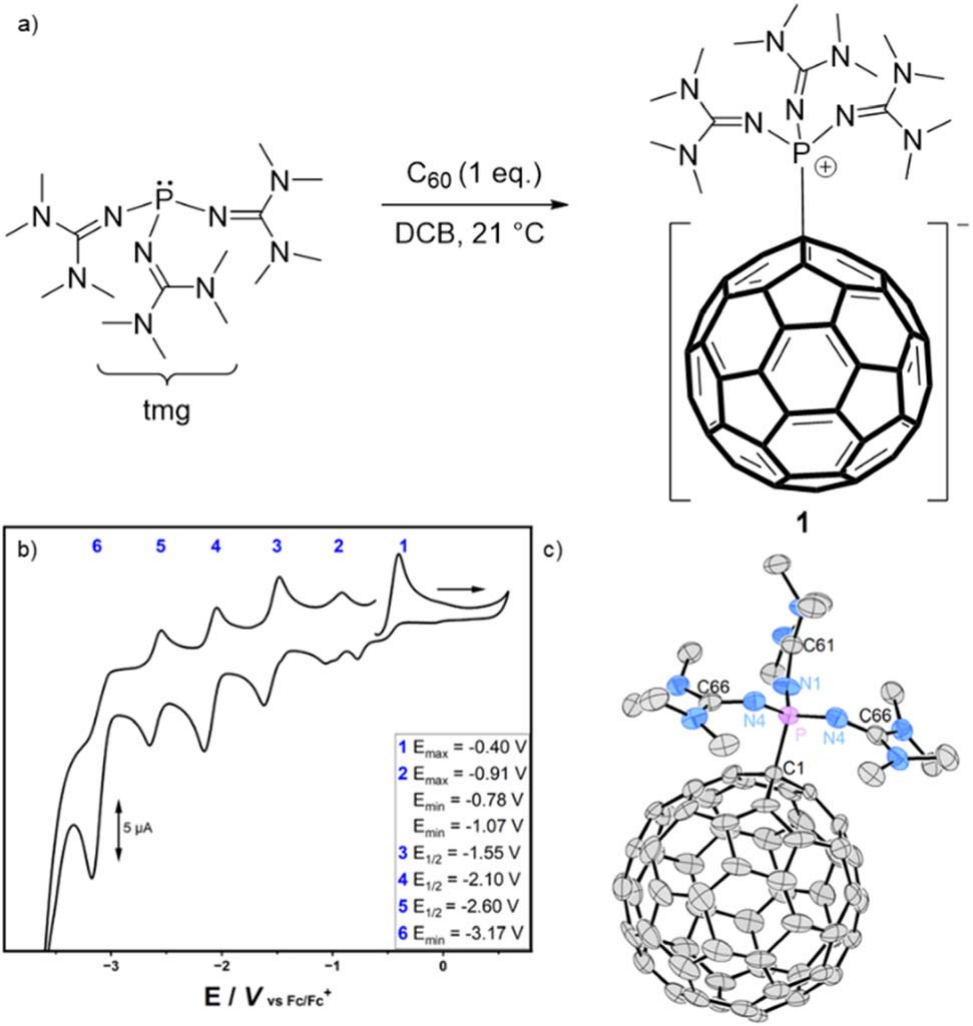
(a) Reaction of (tmg)_3_P with C_60_ to give 1; (b) cyclic voltammogram of 1 in THF at a scan rate of 100 mV s^−1^ (Bu_4_NPF_6_, 0.1 M as electrolyte; Pt working electrode); (c) solid-state structure (positional disorder of C_60_ and THF solvent molecule as well as hydrogen atoms are omitted for clarity); thermal ellipsoids are set at 50% probability, selected bond lengths (Å) and angles (°): P–C1 1.873(2), P–N4 1.6021(16), P–N1 1.606(2), N1–C61 1.304(3), N4–C66 1.308(3), N4–P–N1 117.55(7), N4–P–N4 103.18(12).

Inspired by Hobza's prediction,^23^ we initially examined the reactivity of tris(dialkylamino)phosphines with C_60_. However, no reaction between tris(dimethylamino)phosphine or tris(diethylamino)phosphine and C_60_ was observed in the absence of molecular oxygen,^21^ even under irradiation with light at different wavelengths (ESI, Chapter 1.2†). By contrast tris(tetramethylguanidinyl)phosphine, (tmg)_3_P, reacts with C_60_ at room temperature in 1,2-dichlorobenzene (DCB) to form tris(tetramethylguanidinyl)phosphonium fulleride (1, (tmg)_3_P–C_60_) in quantitative yield (Fig. 2a). Compound 1 is isolated as a crystalline dark green solid after solvent removal. It is moderately soluble in THF, DCM and halogenated aromatics. In contrast to the air-sensitive free phosphine, the fullerene adduct 1 is stable in wet THF and can be briefly handled in air as a solid (see the ESI† for further details). The formation of the zwitterionic adduct is indicated by the pronounced highfield shift of the ^31^P resonance *δ* = −18.7 ppm (P(tmg)_3_: *δ* = 83.5 ppm). Phosphorus carbon coupling constants between the phosphonium center and the C_60_-cage (^1^*J*_PC_ = 147 Hz and ^2^*J*_PC_ = 9 Hz) are deduced from carbon satellites in the ^31^P{^1^H} NMR spectrum as well as from the corresponding resonances in the ^13^C{^1^H} NMR spectrum.

An X-ray diffraction study of single crystals obtained from a concentrated tetrahydrofuran solution confirmed that 1 is an adduct of the phosphine (tmg)_3_P and C_60_ connected *via* a single phosphorus–carbon bond (Fig. 2c). The bond length (1.873 Å) is in the range of a σ-single bond and in good agreement with the P–C bond length of the zwitterionic carbon dioxide complex (tmg)_3_P–CO_2_ (1.879 Å).^29^ The sum of N–P–N angles (338.3°) shows a planarization of the P moiety relative to the free phosphine (296.2°) which is slightly more pronounced than in the CO_2_ adduct (332.3°).

We next studied the electrochemical properties of 1 using cyclic voltammetry (Fig. 2b and S10–S14†). Four sequential reduction events were observed during the reductive sweep (Fig. 2b, 3–6), consistent with the stepwise reduction of the fulleride core of R_3_P–C_60_ (1), aligning with established fullerene electrochemistry.‡‡Due to the limitations imposed by the solvent window, it remains unclear whether the fourth reduction of 1 is reversible, as would be expected for fullerenes. Notably, up to six reduction events have been observed for buckminsterfullerene, albeit at lower temperatures.^41^ These reduction processes remain consistent upon repeated cycling, reversing the scan direction, or using lower scan rates. Furthermore, the observed reduction potentials do not match those of buckminsterfullerene (Fig. S15†), indicating that the fullerene–phosphine adduct remains intact throughout the redox events during the cyclic voltammetry. Remarkably, the first reduction potential of 1 (*E*^red1^_1/2_ = −1.55 V) appears at significantly more negative potential than both that of the first and second reduction waves of pristine C_60_ (*E*^red1^_1/2_ = −0.82 V, *E*^red2^_1/2_ = −1.35 V). This observation is attributed to the zwitterionic nature of 1, consisting of a cationic phosphonium moiety bound to an anionic fulleride core. The addition of an electron to 1 during the first reduction therefore produces a radical dianionic fulleride moiety. By contrast, the dianionic fulleride C_60_^2−^ is generated at more positive potential due to the more efficient delocalization of the π electrons. The similarity in redox chemistry to the dianionic fulleride C_60_^2−^ can be explained using the orbital interaction diagram (Fig. S48†). This diagram shows that the formation of the P–C bond, arising from the orbital overlap between the HOMO of (tmg)_3_P and the LUMO of C_60_, results in a doubly occupied, high-energy, C_60_-centered HOMO. At the same time, the number and energies of the remaining frontier orbitals of C_60_ remain largely unchanged. Experimental evidence supporting the assignment of the reduction events was obtained by treating 1 with cobaltocene (*E*°(DCM) = −1.33 V *vs.* Fc)^30^ and decamethylcobaltocene (*E*°(DCM) = −1.94 V *vs.* Fc).^30^ While pristine C_60_ is already reduced by cobaltocene,^31^ no reaction was observed between 1 and cobaltocene. However, 1 is readily reduced by decamethylcobaltocene as the color of the solution changes from dark green to dark brown and the resonance of phosphine (tmg)_3_P appears in the ^31^P NMR spectrum of the reaction mixture. This observation shows that the dissociation of the radical anion 1^−^ into free phosphine and C_60_^−^ is energetically favored. However, cleavage of the P–C bond seems to be kinetically hindered, as even the polyanions 1*^n^*^−^ (*n* = 1–4) were persistent during the cyclic voltammetry studies.

**Fig. 3:**
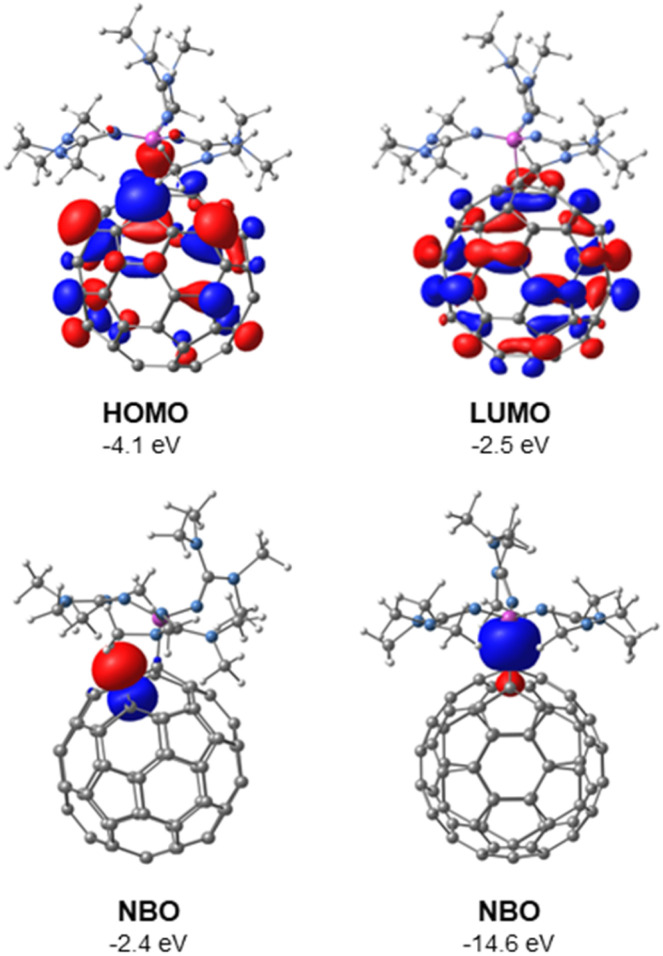
Molecular orbitals (HOMO and LUMO) and two selected natural bond orbitals (NBOs) of 1, determined by DFT using B3LYP(GD3BJ)/6-31G(d,p)/SMD(THF).

In addition to the reduction processes, the voltammogram of 1 exhibits two oxidation processes, labeled 1 and 2 (Fig. 2b). Based on the frontier orbital energies of 1 (Fig. 3 and S46†), the fulleride core undergoes the first oxidation (process 2), resulting in the formation of the corresponding cationic phosphonium fullerenyl radical. The second oxidation process is likely to involve the removal of another electron from the fullerene core since the guanidinyl groups are expected to be oxidized at significantly more positive potentials (for comparison: *E*_max_[{tmg}_4_P^+^] = 0.80 V, Fig. S15†). The resulting phosphonium fullerenyl dication (R_3_P^+^–C_60_^+^) resembles organofullerenyl cations (R–C_60_^+^)^32^ which are important intermediates in fullerene derivatization and can be generated from the corresponding anions (RC_60_^−^) through successive oxidation using rather mild oxidants such as Cu^II^ ions or iodine.^33,34^ Notably, alkylfullerenyl cation intermediates are stabilized by adjacent donor atoms,^33,35^ a scenario that is also conceivable for the guanidinyl nitrogen atoms in 1^2+^.

A UV/Vis spectroscopic analysis of 1 in aromatic solvents (Fig. S15 and S16†) reveals a strong absorption at 330 nm, consistent with that observed for the neutral fullerene molecule.^36^ Additionally, a broad absorption band emerges around 650 nm which has been also observed for fulleride B.^10^

The molecular and natural bond orbitals of 1 were calculated using density functional theory (DFT) at the B3LYP(GD3BJ)/6-31G(d,p)/SMD(THF) level. In analogy to the zwitterionic phosphonium fulleride A,^15^ the highest occupied molecular orbital (HOMO) and lowest unoccupied molecular orbital (LUMO) are both localized on the C_60_ cage. The HOMO (*E* = −4.1 eV) exhibits its highest orbital coefficients adjacent to the C–P bond, while the LUMO (*E* = −2.5 eV) is more centrally located on the C_60_ cage (Fig. 3 top). The natural bond orbitals (NBO) analysis indicates that the highest energy orbital corresponds to the lone pair on the carbon atom positioned adjacent to the quaternary carbon atom, both situated between two hexagonal rings (Fig. 3 bottom left, *E* = −2.4 eV, occupancy: 1.17 e^−^, 99.43% p orbital character).§§The next NBO lower in energy represents an aromatic π bond of two carbon atoms of the C_60_ cage (*E* = −6.3 eV, occupancy: 1.62 e^−^), NBOs representing lone pairs at the nitrogen atoms of the phosphonium unit are even lower in energy (*E* = −8.3 eV to *E* = −6.9 eV). The NBO analysis furthermore confirms the σ-type nature of the C–P bond (Fig. 3 bottom right, Wiberg bond index = 0.72, bond length: 1.886 Å).

### Reactivity of 1 with selenium and electrophiles

To evaluate the reversibility of the reaction between C_60_ and (tmg)_3_P, gray selenium was added to a solution of 1 in THF. No reaction was observed at ambient temperature. However, upon heating the mixture to 100 °C for 16 hours, complete conversion to the corresponding phosphine selenide was achieved, indicating dissociation of the P–C bond at elevated temperature.

The potential frustrated Lewis pair (FLP) character of the zwitterionic fullerene–NHC adduct A was highlighted by Alcarazo and coworker.^37^ To investigate the possible ambiphilic behavior of 1, THF solutions of 1 were treated with carbon dioxide, dihydrogen, and diphenylacetylene, and the reaction mixtures were heated up to 100 °C. NMR analysis revealed no reaction with these substrates, which are known to undergo transformations with FLPs.^38^

**Fig. 4:**
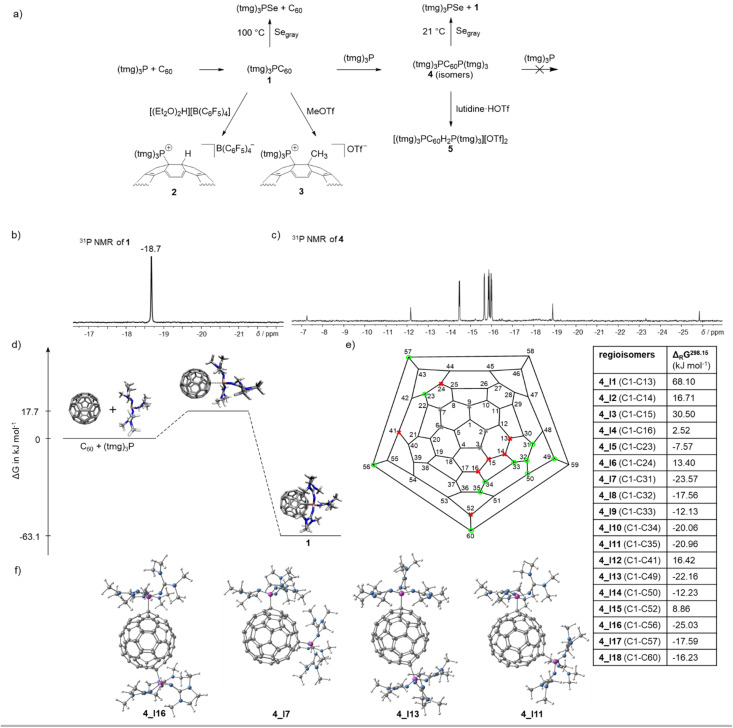
(a) Overview of the reactivity of 1 and 4; (b) ^31^P NMR spectrum of the monoadduct 1 in THF; (c) ^31^P NMR spectrum in THF of the bisadducts (tmg)_3_PC_60_P(tmg)_3_ (4) obtained as mixture of positional isomers; (d) reaction energy profile for the formation of 1 determined by DFT using B3LYP(GD3BJ)/6-31G(d,p)/SMD(THF); (e) calculated free energies for the formation of the positional isomers 4 according to: 1 + (tmg)_3_P → 4 determined by DFT using B3LYP(GD3BJ)/6-31G(d,p)/SMD(THF), Schlegel diagram of C_60_ with possible structural isomers^39^ if one phosphonium center is located at C1 (gray: positional isomers with less than three CC bond between the phosphonium centers that were not considered for steric reasons, red: endergonic isomers, green: exergonic isomers); (f) optimized geometries of the four most stable isomers of 4.

The novel phosphine–fullerene adduct 1 was reacted with electrophiles (E) to investigate the feasibility of further functionalizing the anionic fulleride moiety. Treatment with proton and methyl cation sources resulted in the selective formation of a single regioisomer of the cation [(tmg)_3_P–C_60_–E]^+^ accompanied by a color change from green to brown¶¶This color change is also observed when water is added to 1, in which case the products could not be identified due to their insolubility. (Fig. 4a and Table 1). Specifically, the stochiometric reaction of 1 with [H(Et_2_O)_2_][B(C_6_F_5_)_4_] in dichloromethane quantitatively yielded [(tmg)_3_PC_60_H][B(C_6_F_5_)_4_] (2). Consistent with the NBO analysis, the protonation was found to occur at the carbon atom adjacent to the P–C bond, as evidenced by the characteristic ^3^*J*_PH_ coupling constant of 32 Hz (Table 1). Additionally, the ^1^H–^13^C{^1^H} heteronuclear multiple bond correlation (HMBC) 2D NMR spectrum revealed long-range coupling between the newly attached proton and the quaternary carbon bonded to the phosphonium unit. It is noteworthy that 2,6-lutidinium triflate is also sufficiently acidic to protonate 1. However, attempts to remove 2,6-lutidine by heating to 90 °C under reduced pressure led to the formation of insoluble products. Treatment of 1 with methyl triflate in dichloromethane gave [(tmg)_3_PC_60_CH_3_][OTf] (3) in quantitative yield. The observation of a single set of signals in ^1^H, ^13^C and ^31^P NMR spectra suggests the selective methylation adjacent to the P–C bond, in agreement with the protonation reaction and NBO analysis. The ionic fullerene derivatives 2 and 3 exhibit good solubility in polar solvents such as dichloromethane, which is significantly better than that of the neutral adduct 1.

**Table 1:** Selected NMR chemical shifts *δ* in ppm of 1, 2, and 3 recorded in THF-d_8_ (1) and CD_2_Cl_2_ (2 and 3)

	1 (tmg)_3_PC_60_	2 [(tmg)_3_PC_60_H][B(C_6_F_5_)_4_]	3 [(tmg)_3_PC_60_CH_3_][OTf]
^31^P of C–*P*	−18.7	−20.7 (^3^*J*_PH_ = 32 Hz)	−27.3
^13^C of *C*–P	79.0	73.8	69.5
^13^C of *C*–E	—	59.3	56.0

### Synthesis and reactivity of fullerene adducts with two phosphines (4)

Monitoring the stoichiometric reaction of (tmg)_3_P with C_60_ by ^31^P NMR spectroscopy revealed the initial appearance of several new ^31^P resonances between −7 and −26 ppm. Over time, these resonances decreased in intensity, while the resonance of 1 at −18.7 ppm increased. This observation suggests the transient addition of more than one phosphine to C_60_, caused by the poor solubility of C_60_, which temporarily creates an excess of phosphine in solution. Indeed, varying the ratio of C_60_ to (tmg)_3_P reveals that up to two phosphines can attach to the fullerene core, forming a mixture of regioisomers (tmg)_3_PC_60_P(tmg)_3_ (4) (Table S1†). Adding more than two equivalents of phosphine does not lead to further association with the negatively charged C_60_ core. The bisphosphine fullerene adducts (4) exhibit ^31^P NMR resonances between −7 and −26 ppm (Fig. 4c), which is in a similar range to that of 1 (*δ* = −18.7 ppm). The most intensive resonances appear as two pairs of doublets at *δ* = −14.5 and −15.9 ppm and at *δ* = −15.7 and −16.0 ppm, with phosphorus–phosphorus coupling constants of *J*_PP_ = 3.2 and 3.7 Hz, respectively. The two pairs of doublets correspond to two regioisomers as confirmed by ^31^P–^31^P COSY spectroscopy (Fig. S37†). Furthermore, five singlets appear at *δ* = −7.3, −12.2, −15.9 ppm, −18.9, and −25.8 ppm, which either originate from five individual isomers with isochronous phosphonium centers or may also represent regioisomers with different ^31^P chemical shifts where the *J*_PP_ coupling is unresolved. Hence, among the 23 possible regioisomers of disubstituted fullerene RC_60_R^39^ (Fig. 4e), a total of five to seven isomers were detected *via*^31^P NMR spectroscopy. Notably, variable-temperature ^31^P and ^31^P–^31^P NOESY/EXSY NMR spectroscopy experiments at 70 °C reveal that the regioisomers of 4 undergo interconversion at elevated temperatures (Fig. S36 and S38†).

The assignment of the ^31^P NMR resonances to different regioisomers 4 is further supported by the addition of 2,6-lutidinium triflate, acting as proton source, to the isomeric mixture 4. The resulting ^31^P NMR spectrum shows several new resonances with chemical shifts and ^3^*J*_PH_ coupling constants similar to those of 2 (Fig. S41†). As expected, the number of regioisomers of the dications [((tmg)_3_P)_2_C_60_H_2_]^2+^ (5) increases, since the protons can attach to three different carbon sites adjacent to each phosphonium center.

Further evidence for the composition of 4 was obtained through its reaction with gray selenium, which produced 1 and (tmg)_3_PSe in a 1 : 1 ratio. Notably, this reaction occurs readily at ambient temperature, indicating that the P–C_60_ bond dissociation energy is significantly lower than that of 1. This observation can be rationalized by the increased negative charge imparted to the fullerene core with each successive phosphine complexation.

Collectively, these experiments demonstrate that up to two phosphines (tmg)_3_P can reversibly bind to C_60_. The formation of the first and second dative P–C bonds occurs with low energy barriers, as indicated by the rapid formation of 4 with excess phosphine. However, the P–C bonds in 4 are less stable than in 1, enabling selective conversion of 4 to 1 in the presence of free C_60_. The reactivity and trapping reactions with gray selenium indicate heterolytic dissociation of the dative P–C bond into neutral phosphine and fullerene fragments.

### Mechanistic investigations

Mechanistic investigations using DFT at the B3LYP(GD3BJ)/6-31G(d,p)/SMD(THF) level of theory reveal that the formation of compound 1 from C_60_ and (tmg)_3_P follows a low-energy pathway with a transition state barrier of Δ*G*^‡^ = 17.7 kJ mol^−1^ and an exergonic free energy change of Δ*G* = −63.1 kJ mol^−1^ (Fig. 4d). This aligns with experimental observations of a reversible reaction at elevated temperatures. An alternative electron transfer (ET) mechanism was considered, but the generation of the radical ion pair [(tmg)_3_P^+^˙][C_60_^−^˙] was found to be significantly less favorable, with a minimum energy barrier of 41.8 kJ mol^−1^. This contrasts with the literature reports on the C_60_–DBU system,^18^ where a stepwise ET mechanism was proposed based on the decrease in EPR signal intensity of the C_60_˙^−^ radical anion over time.

To determine whether radical species are generated during the formation of the zwitterionic adduct 1, DCB solutions containing (tmg)_3_P and C_60_ in various stoichiometric ratios were analyzed by EPR spectroscopy. No radical species were detected with an excess of C_60_. However, in a 1 : 1 stoichiometric mixture or in the presence of excess phosphine, at least three paramagnetic species were observed. These species were assigned to the C_60_˙^−^ radical anion and two phosphorus-containing radicals (ESI, Chapter 1.6.5†). Unlike the DBU–C_60_ system, the signal intensity of these species remained constant over time. The presence of paramagnetic species implies that ET processes or homolytic P–C bond cleavage may occur to some extent. However, the high selectivity of the trapping reactions, combined with the lack of reactivity between 1 and diphenylacetylene upon heating, indicates that these processes play a minor role. Instead, they are likely associated with ET from the electron rich bisadducts 4 (Fig. S44†).

DFT calculations were also performed to investigate the second addition of the phosphine to 1 and to assign the positional isomers of 4. Among the 23 possible positional isomers of RC_60_R (R = (tmg)_3_P),^39^ five were excluded due to the spatial proximity of the phosphonium centers (Fig. 4e). For the remaining 18 isomers (4_I1 to 4_I18), ^31^P NMR chemical shifts and the free energies of formation were calculated. The computed ^31^P NMR chemical shifts (*δ* = 2 to −23 ppm, Table S6†) fall within the observed range, but are not precise enough for definite isomer assignment. Free energy calculations (Fig. 4e) reveal that the distance between the phosphonium centers significantly influences stability. Isomers with three carbon–carbon bond separations (4_I1, Fig. S52† and 4_I3) are highly endergonic, while those with four CC bonds (4_I2, 4_I4 and 4_I6, Fig. S52†) are less endergonic. Starting at five CC bonds, isomers become exergonic (Δ*G* = −7 to −25 kJ mol^−1^). Exceptions, such as 4_I12 and 4_I15, demonstrate that the electronic structure also affects regioisomer stability. Overall, the DFT results suggest the existence of 11 stable isomers with varying stabilities. While steric factors significantly influence stability, electronic effects appear to play only a minor role. However, due to their similar stabilities, a definitive assignment of the isomers observed in the ^31^P NMR spectrum was not possible.

## Conclusions

Herein, we report the preparation of the first adducts between buckminsterfullerene (C_60_) and a phosphine. The electron-rich phosphine (tmg)_3_P reacts selectively with C_60_*via* a concerted nucleophilic addition pathway to form the zwitterionic phosphonium fulleride 1, a stable crystalline adduct featuring a dative P–C bond. In the presence of excess phosphine, a second phosphine rapidly binds to the C_60_ core yielding bisphosphine fullerene adducts 4 as a mixture of regioisomers. Given the increased negative charge of the C_60_ core with each successive phosphine addition, this behavior highlights the high nucleophilicity of the superbasic phosphine (tmg)_3_P and has not been observed for other Lewis bases.^15–18^ The formation of stable adducts between (tmg)_3_P and C_60_ agrees with predictions from Hobza,^23^ and suggests that phosphines bearing the same or greater basicity should also form adducts with C_60_ of other fullerenes. Trapping experiments provide evidence for heterolytic P–C bond dissociation and demonstrate the reversibility of the adduct formation. Furthermore, functionalization of 1 with electrophiles provides access to ionic fullerene derivatives, illustrating the potential for further chemical modifications. These findings contribute to a deeper understanding of phosphine–fullerene interactions and pave the way for the development of new functional fullerene derivatives with tailored properties. Potential applications of these compounds may extend from semiconductor technology to molecular rotors and integration into optoelectronic devices.^6,40^

## Author contributions

M. B. R. performed the experiments. DFT calculations were performed by J. H. F. and T. S. H. D. L. performed EPR measurements. SCXRD studies were performed by M. S. F. D. directed the investigation. The manuscript was written by M. B. R. and F. D. All authors have given approval for the final version of the manuscript.

## Conflicts of interest

There are no conflicts to declare.

## Supplementary Material

SC-OLF-D5SC00367A-s001

SC-OLF-D5SC00367A-s002

## Data Availability

Further details of the experimental procedures, the computational studies, and the characterization data for the new compounds have been included as part of the ESI.† Crystallographic data for 1 has been deposited at the CCDC under 2411442 and can be obtained from https://www.ccdc.cam.ac.uk/structures/.
