# Supplementary material for: Phosphonium fullerides: isolable zwitterionic adducts of a phosphine with C60
Source: Chem Sci. 2025 Jun 26;16(29):13189–95. doi: 10.1039/d5sc00367a (PMC12207991; doi:10.1039/d5sc00367a)
Supplement: SC-016-D5SC00367A-s001 [file SC-016-D5SC00367A-s001.pdf]

Supplementary Information (SI) for Chemical Science.
This journal is © The Royal Society of Chemistry 2025

Supplementary Material for

Phosphonium Fulleride: Isolable Zwitterionic Adducts of a Phosphine with C_{60}

Maike B. Röthel[a], Jonas H. Franzen[a], Daniel Leitner[a], Thomas S. Hofer[a], Michael Seidl[a], Fabian Dielmann[a]*

[a] Maike B. Röthel, Jonas H. Franzen, Daniel Leitner, Assoz.-Prof. Dr. Thomas Hofer, Dr. Michael Seidl, Prof. Dr. Fabian Dielmann
Institute of General, Inorganic and Theoretical Chemistry,
Universität Innsbruck, 6020 Innsbruck (Austria)
mail: Fabian.Dielmann@uibk.ac.at

CONTENT

1 Experimental Procedures 3

1.1 Synthetic Details 3

1.2 Reactivity of Tris(dialkylamino)phosphines towards C_{60} 4

1.3 Synthesis and Characterization of Tris(tetramethylguanidinyl) phosphonium Fulleride 1 6

1.4 Protonation of 1 15

1.5 Methylation of 1 19

1.6 Detailed Studies of the Reaction of C_{60} and $(tmg)_3P$ 23

1.6.1 Stoichiometry Investigations 23

1.6.2 NMR spectroscopic investigations of 4 24

1.6.3 Protonation of 4 26

1.6.4 Reversibility Study: Reaction of 1 and 4 with gray selenium 27

1.6.5 EPR studies 29

2 X-ray Crystallography 31

2.1 Crystal Structure Data of Compound 1 31

3 Computational Details 33

3.1 General 33

3.2 Orbitals of 1 34

3.3 Mechanistic Investigations 38

3.4 Free Energies and Chemical Shifts of 4 39

3.5 Cartesian Coordinates of the Optimized Geometries 47

4 References 75

1 Experimental Procedures

1.1 Synthetic Details

General Remarks: Unless otherwise noted, all manipulations were performed under an inert atmosphere of dry argon, using standard Schlenk and drybox techniques. Dry and oxygen-free solvents were employed. All glassware was oven-dried at 150 °C prior to use. ^{1}H, ^{13}C, ^{19}F and ^{31}P NMR spectra were recorded on 400 MHz Bruker AVANCE 4 Neo spectrometers and a 300 MHz Bruker AVANCE spectrometer. Chemical shifts (δ) are given in parts per million (ppm) relative to $SiMe_4$ (^{1}H, ^{13}C), 85% H_3PO_4 (^{31}P) and $CFCl_3$ (^{19}F) and they were referenced to the residual solvent signals (THF-d_8: δ_H = 1.73 , 3.58 ppm, δ_C = 67.57, 25.37 ppm; CD_2Cl_2: δ_H = 5.32 ppm, δ_C = 54.00 ppm) or internally by the instrument after locking and shimming to the deuterated solvent (^{31}P, ^{19}F). NMR multiplicities are abbreviated as follows: s = singlet, d = doublet, m = multiplet, br. = broad signal. High resolution mass spectrometry was performed using an Orbitrap LTQ XL (Thermo Scientific) spectrometer. Elemental analysis was performed using a 'vario MICRO cube' elemental analyzer. IR spectroscopy was performed using a Bruker ALPHA II FT-IR spectrometer. IR transmittance intensity is abbreviated as follows: w = weak, m = medium, s = strong, vs = very strong. Electrochemical measurements were performed using the following Potentiostat/Galvanostat/ZRA: Gamry Interface 1010B. UV/Vis and NIR absorption measurements were performed with a combination of Avantes AvaLight DM-S-BAL as light source and multichannel spectrometer AvaSpec NIR-1.7 as detector. EPR measurements were performed on a Brucker Magnettech MS5000 X-band spectrometer equipped with a temperature control unit in 3 mm teflon-sealed silica tubes. Spectra were processed using the EasySpin package for Matlab®.[1]

Reagents: Tris(tetramethylguanidinyl)phosphine[2], tetra(tetramethylguanidinyl)phosphonium chloride[3] and $[H(Et_2O)_2][B(C_6F_5)_4]$[4] were prepared following literature procedures.

1.2 Reactivity of Tris(dialkylamino)phosphines towards C_{60}

A stock solution of tris(dimethylamino)phosphine (0.325 M in 1,2-dichlorobenzene, 0.13 mL, 0.042 mmol) or tris(diethylamino)phosphine (0.213 M in 1,2-dichlorobenzene, 0.20 mL, 0.042 mmol) was added to a solution of C_{60} (30 mg, 0.044 mmol) in 1,2-dichlorobenzene (0.5 mL) in a PTFE-sealed NMR tube.

^{31}P NMR spectra were measured after:
20 minutes at room temperature
2 h at room temperature
19 h at room temperature
24 h at room temperature
+ 1 h irradiation with 365 nm
+ 16 h irradiation with 365 nm
+ 3 h irradiation with 6200 K cold white light
+ 1 h irradiation with 280 nm
Find the stacked spectra in Figure S1 and Figure S2.

In a previous investigation by Romanova and coworkers[5] on the reaction of buckminsterfullerene with either tris(dimethylamino)phosphine or tris(diethylamino)phosphine in 1,2-dichlorobenzene, the formation of the diradical ionpair $[C_{60}^{-\cdot}][P(NR_2)_3^{+\cdot}]$ was suggested based on ESR data. The authors report that after the addition of the phosphine to C_{60} a dark green solution is formed which changes its color to dark brown over time. In addition, the respective phosphine oxides were detected by ^{31}P NMR spectroscopy when the reaction mixture was exposed to air.

In our attempt to detect a potential phosphine-C_{60} adduct, a 1:1 mixture of C_{60} and a dialkylaminophosphine in 1,2-dichlorobenzene under inert atmosphere was monitored by ^{31}P NMR spectroscopy. In contrast to the abovementioned report, we did not observe a color change and the ^{31}P NMR spectra did not indicate any reaction between the phosphine and C_{60}.

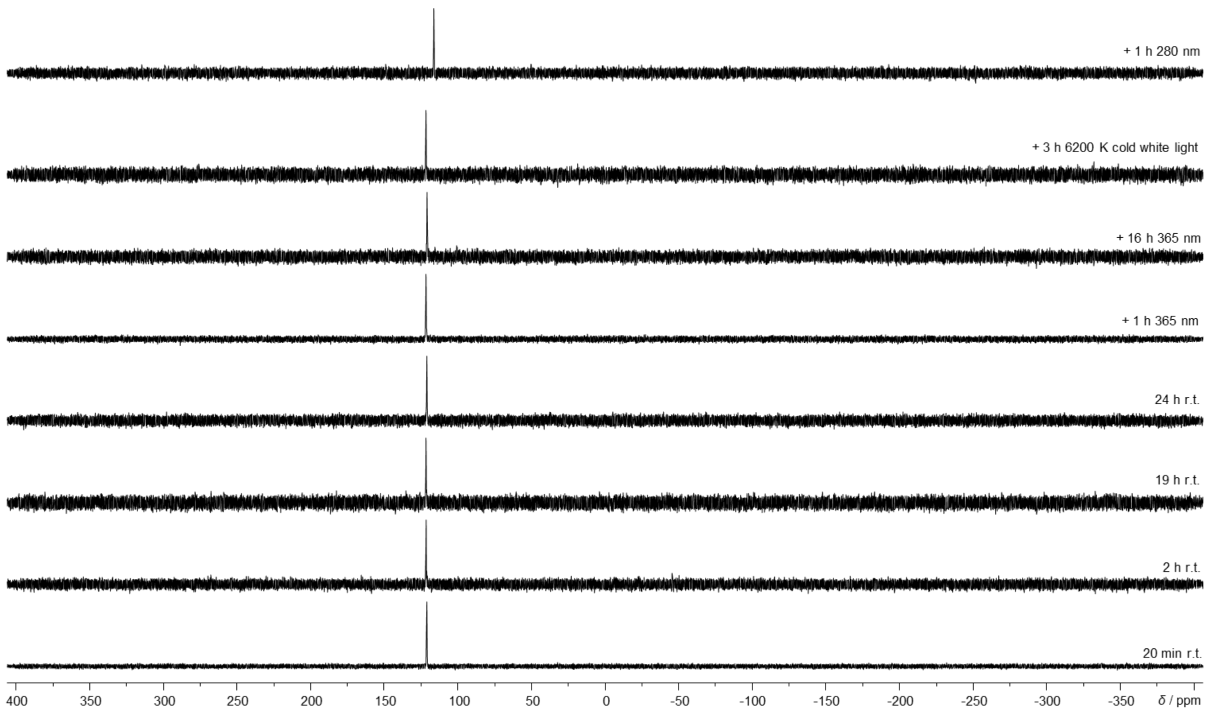

Figure S1: ^{31}P NMR spectra of $P(NMe_2)_3$ and C_{60} in 1,2-dichlorobenzene, signal to noise (S/N) ratio differs in this representation because of different line widths of the resonance.

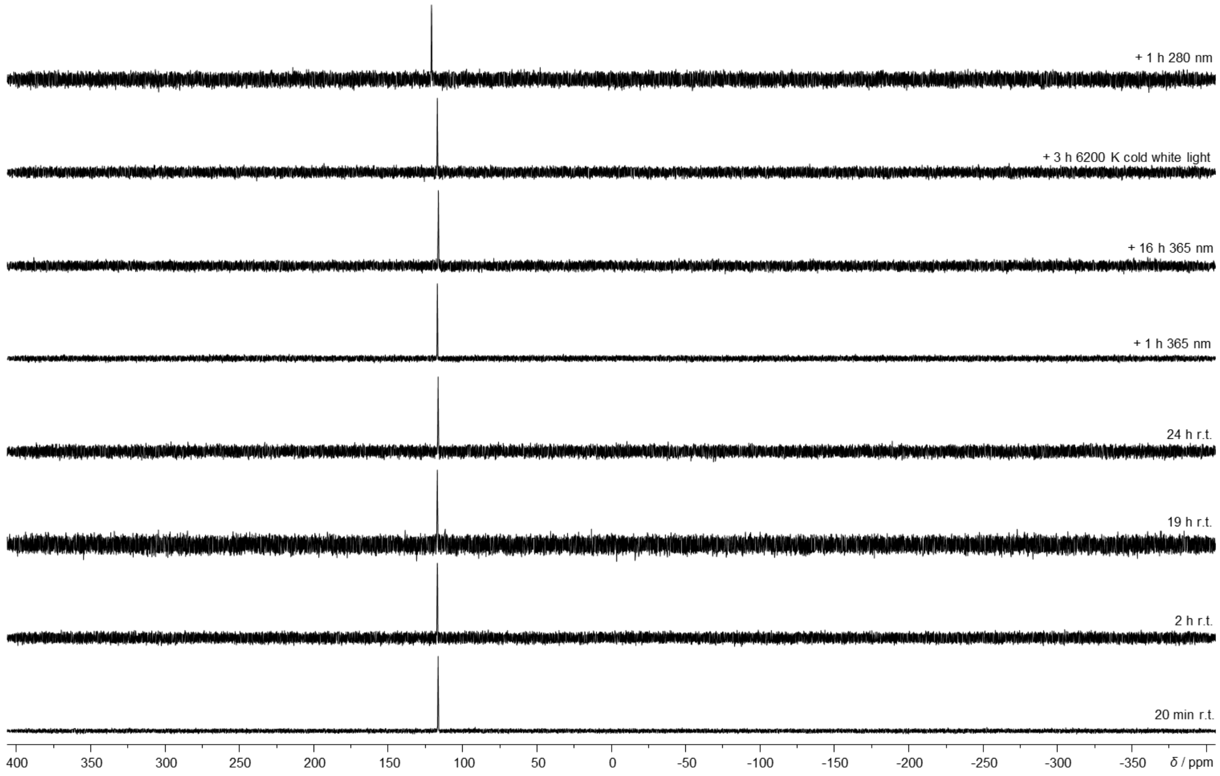

Figure S2: ^{31}P NMR spectra of $P(NEt_2)_3$ and C_{60} in 1,2-dichlorobenzene, signal to noise (S/N) ratio differs in this representation because of different line widths of the resonance.

1.3 Synthesis and Characterization of Tris(tetramethylguanidinyl) phosphonium Fulleride 1

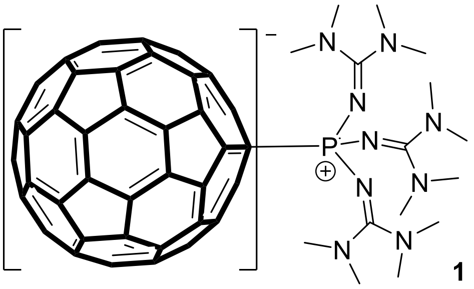

1

1,2-Dichlorobenzene (2 mL) was added to a Schlenk tube containing buckminsterfullerene C_{60} (100 mg, 0.139 mmol, 1 eq.) and tris(tetramethylguanidinyl)phosphine (51.8 mg, 0.139 mmol, 1 eq.). A dark green suspension formed upon addition of the solvent. The mixture was stirred for 16 hours and then evaporated to dryness *in vacuo*. Compound **1** was obtained as a dark green solid in quantitative yield (152 mg, 0.139 mmol). It exhibits moderate solubility in tetrahydrofuran, dichloromethane, 1,2-dichlorobenzene and 1,2-difluorobenzene, while being sparingly soluble in acetonitrile, α,α,α-trifluorotoluene and non-polar solvents.

The reaction can also be performed in 1,2-difluorobenzene or tetrahydrofuran. Using tetrahydrofuran requires heating to 100 °C for 16 hours in a sealed vessel (preferably PTFE-sealed). Although the reaction requires a higher temperature, the isolation of **1** is easier because these solvents are more volatile.

Reaction monitoring via ^{31}P NMR spectroscopy shows that **4** is initially the mayor species in the reaction mixture (see Chapter 1.6 for details and 1.6.1 for the ^{31}P NMR spectrum of **4**), owing to the low solubility of C_{60}. Over time, equilibration leads to complete conversion to compound **1**.

To evaluate the stability of compound **1** in the presence of air and moisture, a solid sample was exposed to air for a specific duration and subsequently analyzed using NMR spectroscopy. The fullerene adduct **1** exhibited no significant signs of decomposition after 1 hour under ambient conditions. However, after 24 hours in air, substantial decomposition was observed, resulting in the formation of an insoluble material. These findings indicate that compound **1** can be briefly handled in air, but it should be stored under an inert atmosphere. To assess the hydrolytic stability of **1**, a tenfold excess of degassed water was added to a THF solution of **1**. The analysis revealed no evidence of decomposition, highlighting its resistance to hydrolysis. This behavior contrasts with the rapid hydrolysis observed for the free phosphine in the presence of water. However, consistent with the reversible nature of the adduct formation, compound **1** decomposes in wet THF when heated to 100 °C.

^{1}H NMR (400 MHz, 298 K, THF-d_8): δ (ppm) = 3.19 (s, 36 H, CH_3).

^{13}C{^{1}H} NMR (100 MHz, 298 K, THF-d_8): δ (ppm) = 41.5 (CH_3), 79.0 (d, $^{1}J_{PC}$ = 147 Hz, C_{60}, C–PR$_3$), 120.1 (d, $^{2}J_{PC}$ = 9 Hz, C_{60}, next to C–PR$_3$), 129.1 (C_{60}), 129.8 (C_{60}), 133.3 (C_{60}), 136.5 (d, J_{PC} = 2.6 Hz, C_{60}), 138.6 (C_{60}), 139.9 (C_{60}), 140.4 (d, J_{PC} = 3.4 Hz, C_{60}), 141.4 (C_{60}), 142.6 (C_{60}), 143.6 (d, J_{PC} = 1.9 Hz, C_{60}), 143.7 (d, J_{PC} = 2.9 Hz, C_{60}), 143.8 (C_{60}), 144.1 (C_{60}), 145.2 (C_{60}), 145.7 (C_{60}), 146.0 (C_{60}), 146.3 (C_{60}), 147.2 (C_{60}), 147.6 (C_{60}), 149.0 (C_{60}), 149.5 (d, J_{PC} = 3.7 Hz, C_{60}), 150.3 (d, J_{PC} = 1.3 Hz, C_{60}), 150.6 (C_{60}),151.3 (C_{60}), 151.6 (C_{60}), 152.3 (C_{60}), 152.7 (C_{60}), 153.4 (C_{60}), 155.4 (d, J_{PC} = 2.6 Hz, C_{60}), 161.3 (d, $^{2}J_{PC}$ = 1.8 Hz, N=C(NMe$_2$)$_2$), 161.6 (d, J_{PC} = 2.0 Hz, C_{60}) 177.9 (d, $^{2}J_{PC}$ = 9 Hz, C_{60}, next to C–PR$_3$).

^{31}P NMR (162 MHz, 298 K, THF-d_8): δ (ppm) = –18.7 (s).

HRMS (ESI): m/z calculated for $[C_{75}H_{37}N_9P]^+$ $[1+H]^+$ 1094.2894, found 1094.2904.

CHN analysis: calculated (found) for $[C_{75}H_{36}N_9P]$: C 82.33 (81.48) H 3.32 (3.54) N 11.52 (11.29).

IR (DCB): $\tilde{\nu}$ (cm^{-1}) = 3067 (w), 1573 (w) 1455 (s), 1434 (m), 1252 (w), 1169 (w), 1126 (m), 1034 (s), 941 (w), 743 (vs), 658 (m), 485 (w), 469 (w), 436 (m).

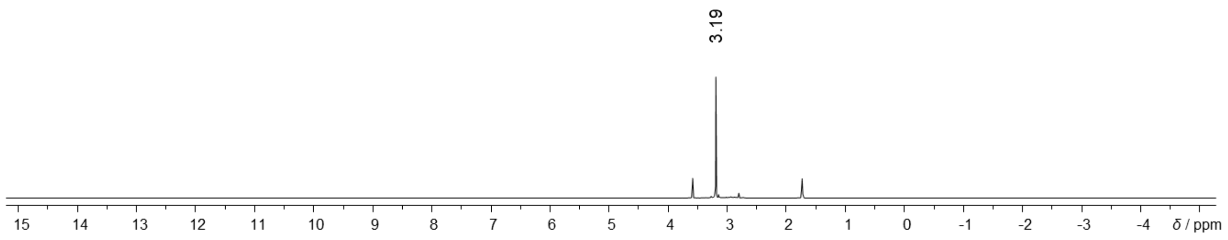

Figure S3: ^{1}H NMR spectrum (THF-d_8, 400 MHz) of **1**.

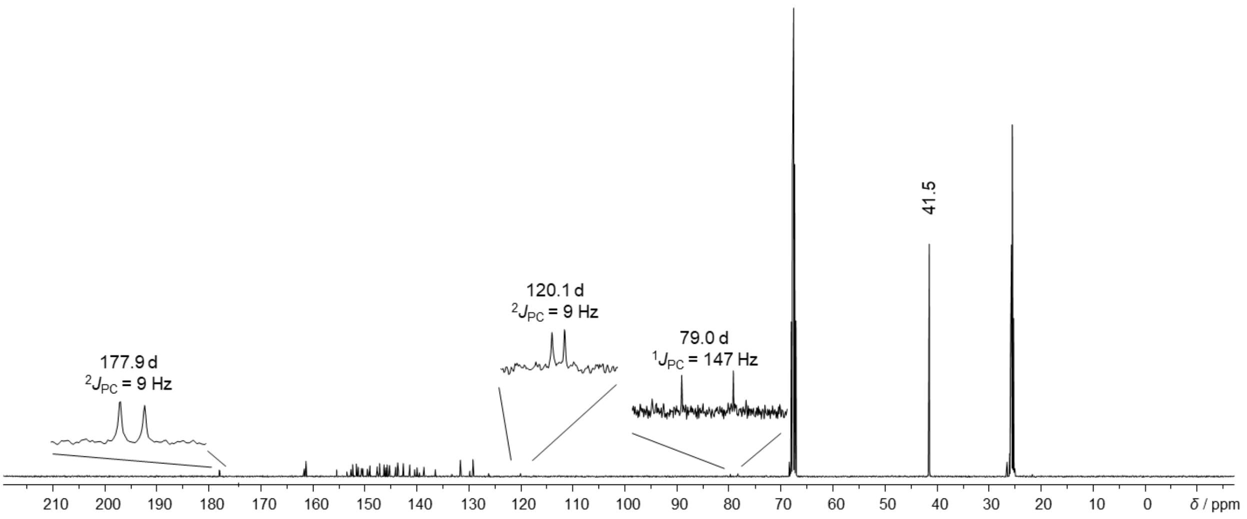

Figure S4: $^{13}C\{^1H\}$ NMR spectrum (THF-d_8, 100 MHz) of **1**, for detailed zoom on the region of δ = 121 to 165 ppm see Figure S5.

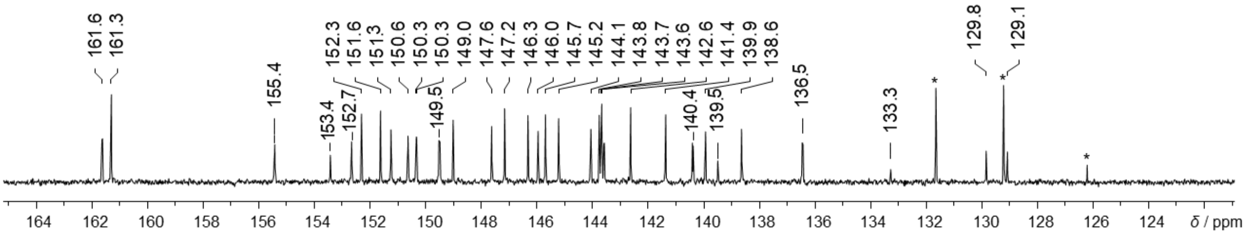

Figure S5: $^{13}C\{^1H\}$ NMR spectrum (THF-d_8, 100 MHz) of **1**, zoomed in from δ = 121 to 165 ppm, asterisks (*) mark residual 1,2-difluorobenzene.

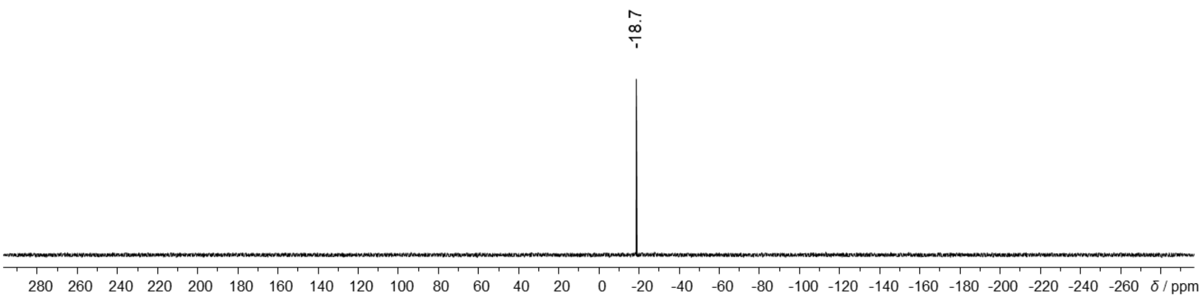

Figure S6: ^{31}P NMR spectrum (THF-d_8, 162 MHz) of **1**.

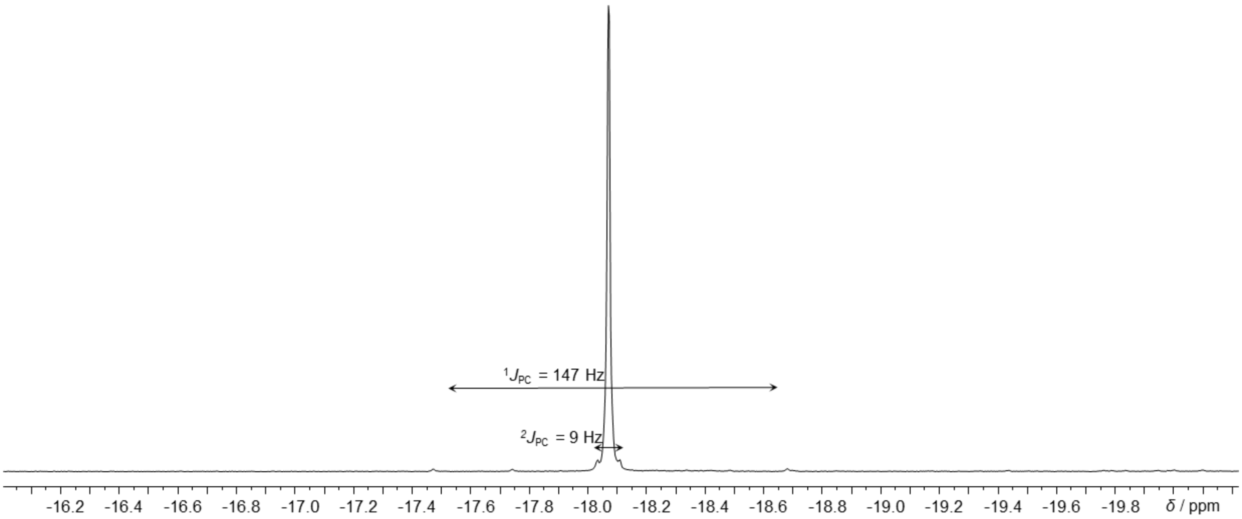

Figure S7: ^{31}P{^{1}H} NMR spectrum (THF-d_8, 121 MHz, 4480 Scans, 50° pulse sequence) of **1**, focusing on the P–C coupling constants.

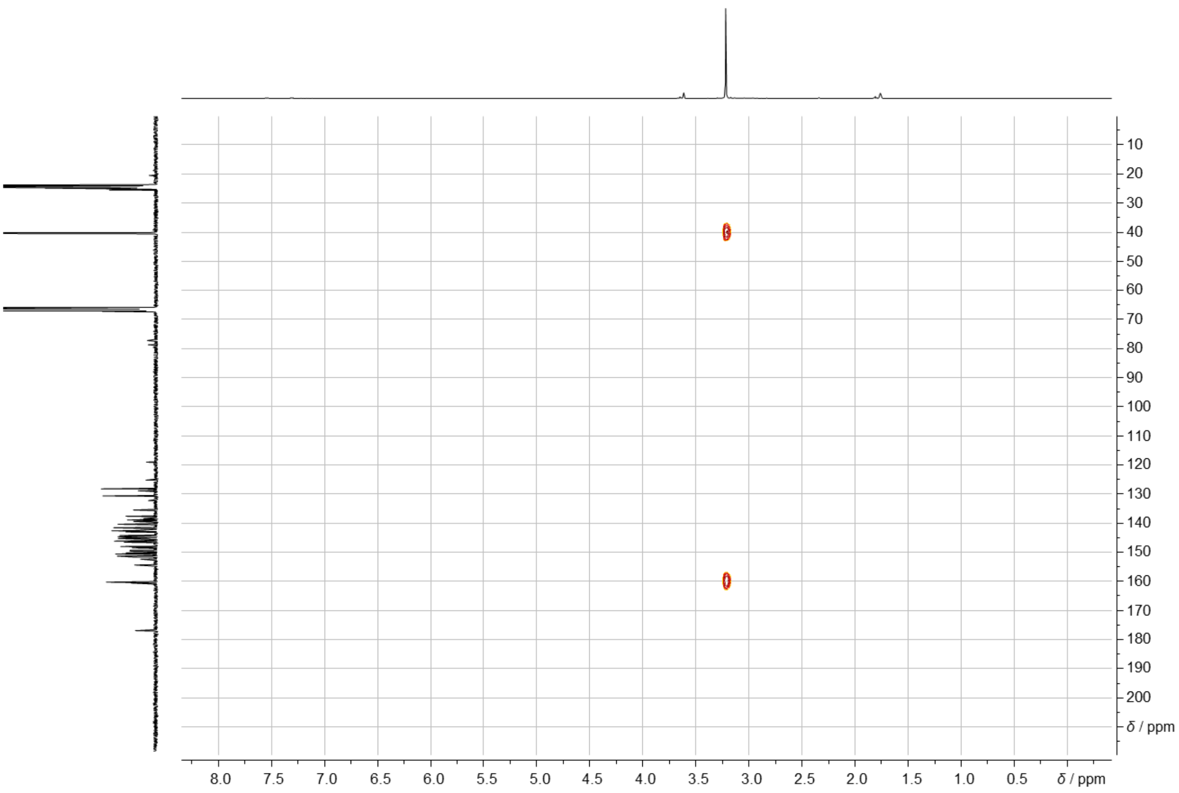

Figure S8: ^{1}H $^{13}C\{^{1}H\}$ HMBC NMR spectrum (THF-d_8, 400 MHz, 100 MHz) of **1**.

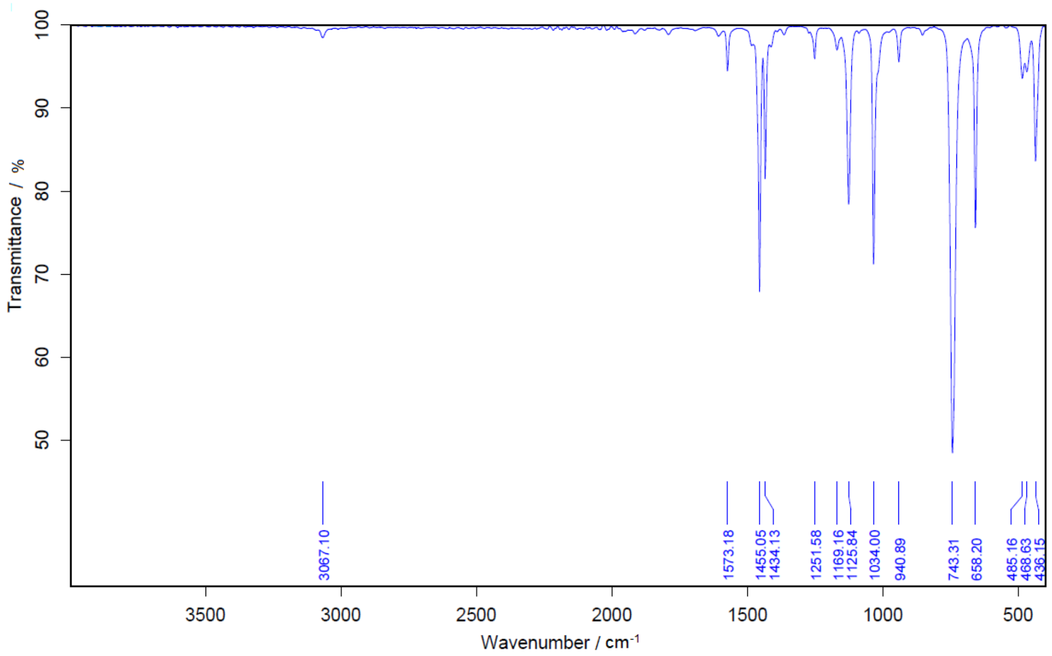

Figure S9: IR spectrum of **1** in 1,2-dichlorobenzene.

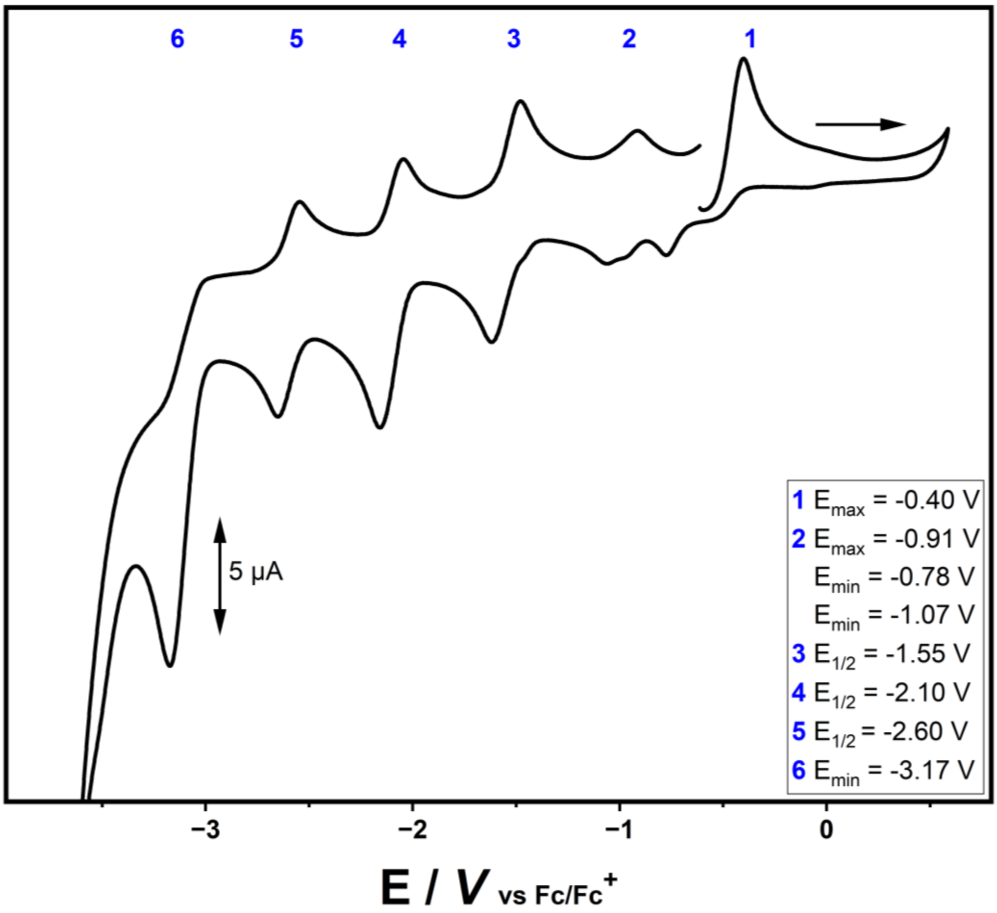

Figure S10: Cyclic voltammogram of **1** in THF at a scan rate of 100 mV/s (Bu_4NPF_6, 0.1 M as electrolyte; Pt working electrode, Pt counter electrode, Ag wire as pseudo-reference electrode, referenced internally against Fc/Fc$^+$), six processes observed (1-2: irreversible oxidations of the fulleride core, 3-6: (reversible) reduction of the fulleride moiety).

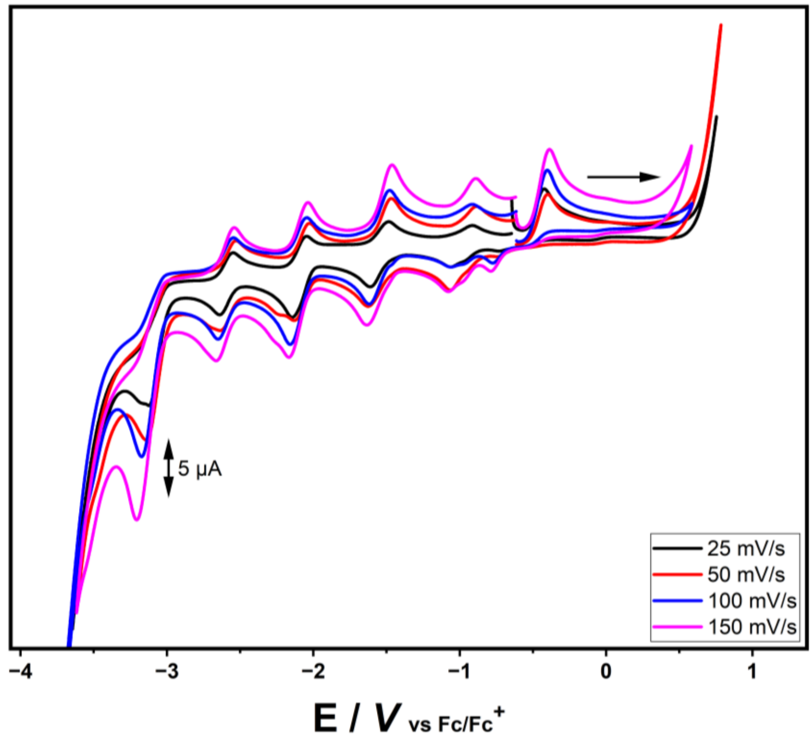

Figure S11: Cyclic voltammograms of **1** in THF at different lower scan rates (Bu_4NPF_6, 0.1 M as electrolyte; Pt working electrode, Pt counter electrode, Ag wire as pseudo-reference electrode, referenced internally against Fc/Fc^+).

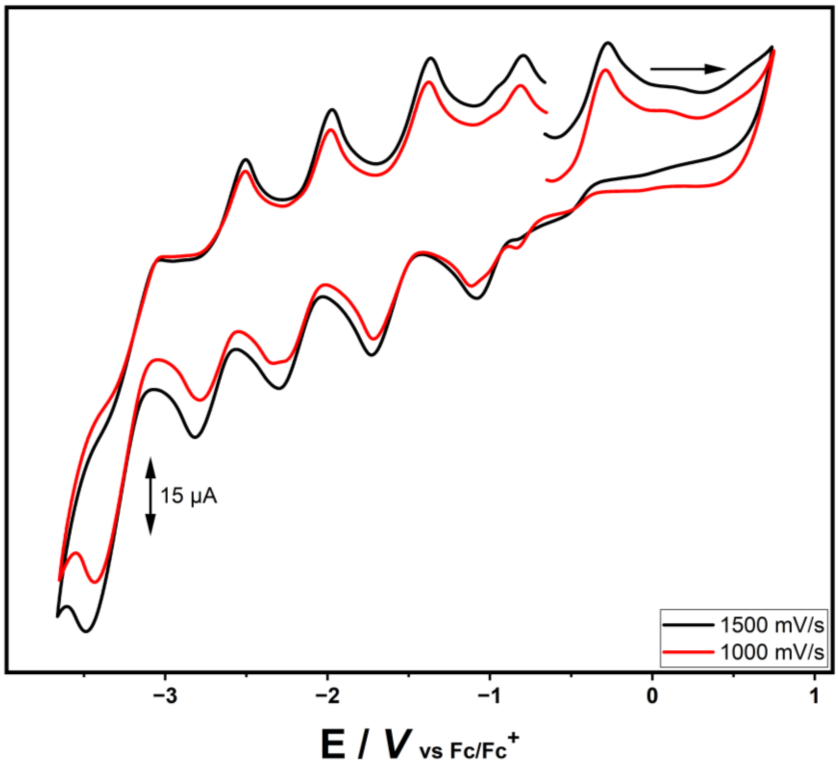

Figure S12: Cyclic voltammograms of **1** in THF at higher scan rates (Bu_4NPF_6, 0.1 M as electrolyte; Pt working electrode, Pt counter electrode, Ag wire as pseudo-reference electrode, referenced internally against Fc/Fc^+).

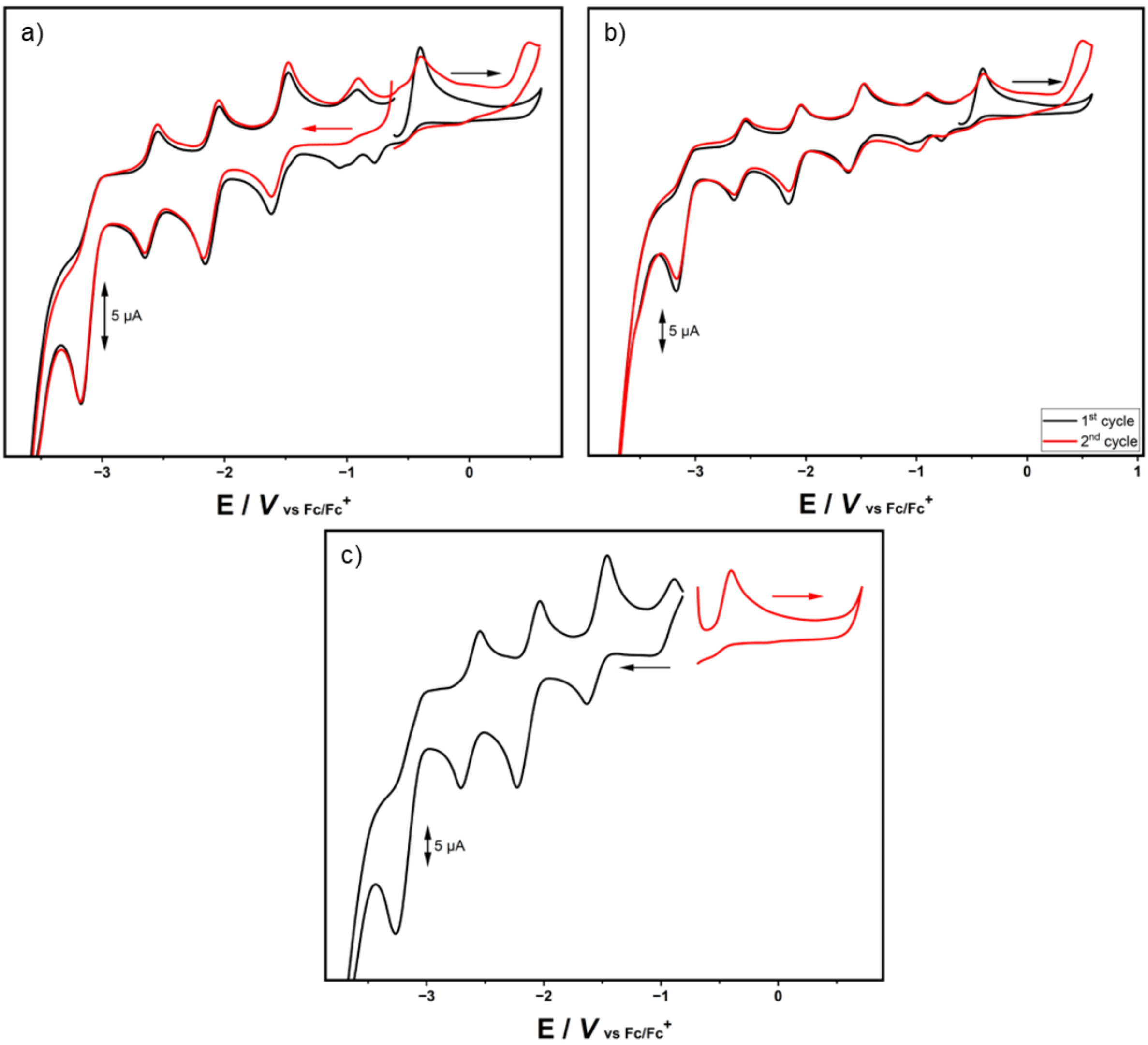

Figure S13: Cyclic voltammograms of **1** in THF at a scan rate of 100 mV: a) different scan directions, b) two cycles, c) positive and negative voltages* measured separately (Bu_4NPF_6, 0.1 M as electrolyte; Pt working electrode, Pt counter electrode, Ag wire as pseudo-reference electrode, referenced internally against Fc/Fc^+), *before referencing.

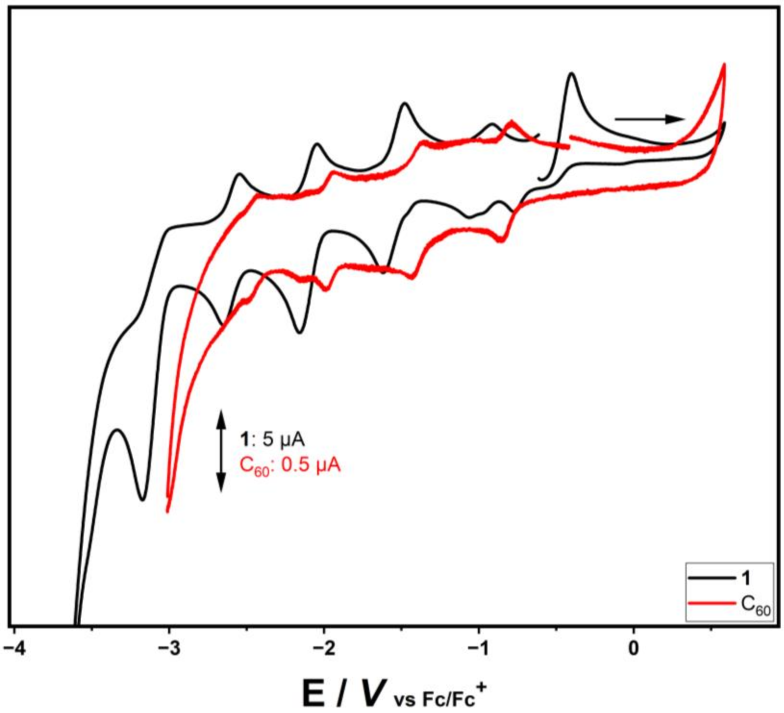

Figure S14: Cyclic voltammograms of **1** and buckminsterfullerene **C_{60}** (less intense signals due to bad solubility) in THF at a scan rate of 100 mV/s (Bu_4NPF_6, 0.1 M as electrolyte; Pt working electrode, Pt counter electrode, Ag wire as pseudo-reference electrode, referenced internally against Fc/Fc^+). For three reductions a difference of the half step potentials of $\Delta E^{red} = E_{1/2}^{red}[C_{60}] - E_{1/2}^{red}[\mathbf{1}]$ could be determined: $\Delta E^{red1} = 0.73$ V, $\Delta E^{red2} = 0.75$ V, $\Delta E^{red3} = 0.64$ V.

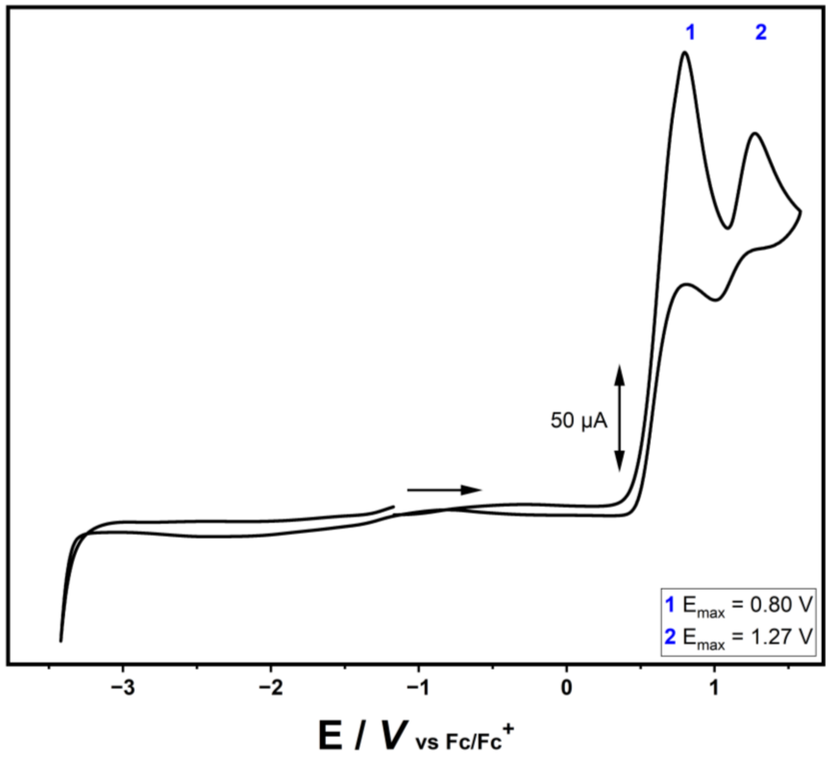

Figure S15: Cyclic voltammogram of $[(tmg)_4P][Cl]$ in ACN at a scan rate of 100 mV/s (Bu_4NPF_6, 0.1 M as electrolyte; Pt working electrode, Pt counter electrode, Ag wire as pseudo-reference electrode, referenced internally against Fc/Fc^{+*}), *decamethylferrocene used as experimental reference, conversion according to literature.[6]

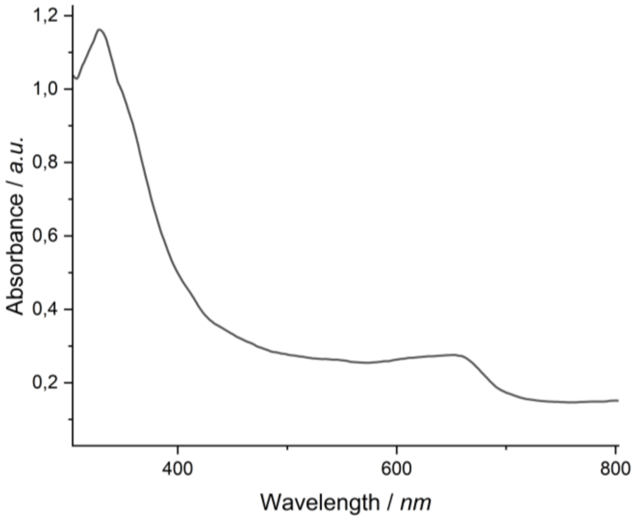

Figure S16: UV/Vis absorption spectrum of **1** (~1.3 x 10^{-2} M) in 1,2-dichlorobenzene showing absorption bands with maxima at *λ* = 328 nm and *λ* = 652 nm.

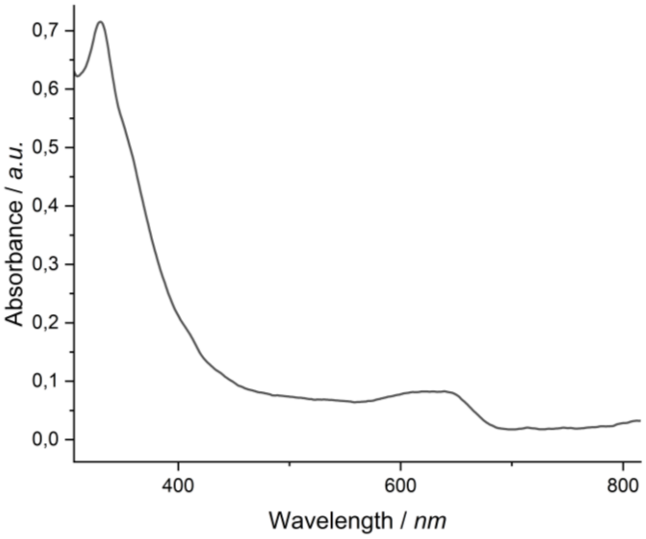

Figure S17: UV/Vis absorption spectrum of **1** in toluene (bad solubility, saturated solution) showing absorption bands with maxima at *λ* = 330 nm and *λ* = 640 nm.

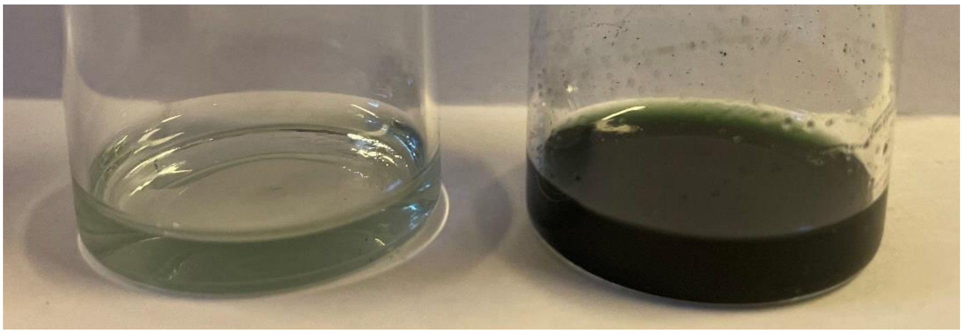

Figure S18: Solutions of **1** in THF (left: diluted, right: saturated).

1.4 Protonation of 1

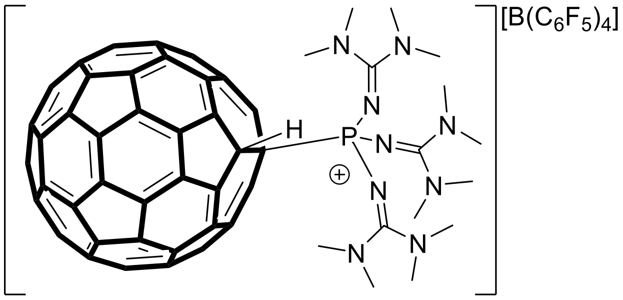

[**1**·H][B(C_6F_5)$_4$]
2

[H(Et_2O)$_2$][B(C_6F_5)$_4$] (27.6 mg, 0.037 mmol, 1 eq.) was added to a solution of **1** (40 mg, 0.037 mmol, 1 eq.) in dichloromethane (1 mL). The reaction mixture was stirred for 2 hours at room temperature leading to a color change to brown. The volatiles were removed *in vacuo* and **2** was obtained as a brown solid in quantitative yield.

^{1}H NMR (400 MHz, 298 K, CD_2Cl_2): δ (ppm) = 7.50 (d, 1 H, $^3J_{PH}$ = 32.4 Hz, *H*-C_{60}), 3.04 (s, 36 H, C*H*$_3$).
^{13}C{^{1}H} NMR (100 MHz, 298 K, CD_2Cl_2): δ (ppm) = 41.3 (*C*H$_3$), 59.3 (C_{60}, *C*–H, next to C–PR$_3$), 73.8 (d, $^1J_{PC}$ = 146 Hz, C_{60}, *C*–PR$_3$), 123.4-125.6 (br., B(C_6F_5)$_4$, *ipso*-C), 136.9 (d, br., $^1J_{CF}$ = 246 Hz, B(C_6F_5)$_4$, *C*–F), 136.5 (d, J_{PC} = 2.5 Hz, C_{60}), 136.6 (d, J_{PC} = 6 Hz, C_{60}), 138.8 (d, br., $^1J_{CF}$ = 245 Hz, B(C_6F_5)$_4$, *C*–F), 140.2 (d, J_{PC} = 1.5 Hz, C_{60}), 141.1 (C_{60}), 142.0 (C_{60}), 142.3 (C_{60}), 142.5 (d, J_{PC} = 3.0 Hz, C_{60}), 142.5 (C_{60}), 142.6 (C_{60}), 142.8 (C_{60}), 143.3 (d, J_{PC} = 5 Hz, C_{60}), 142.8 (C_{60}), 143.3 (C_{60}), 143.3 (C_{60}), 143.6 (C_{60}), 143.9 (C_{60}), 145.0 (d, J_{PC} = 1.2 Hz, C_{60}), 145.7 (d, J_{PC} = 2.5 Hz, C_{60}), 146.0 (C_{60}), 146.0 (C_{60}), 146.1 (C_{60}), 146.4 (C_{60}), 146.8 (C_{60}), 146.9 (C_{60}), 147.0 (C_{60}), 147.1 (C_{60}), 147.7 (C_{60}), 147.8 (C_{60}), 148.6 (d, J_{PC} = 2.1 Hz, C_{60}), 148.7 (d, br., $^1J_{CF}$ = 243 Hz, C–F, B(C_6F_5)$_4$), 152.5 (d, $^2J_{PC}$ = 10 Hz, C_{60}, next to C–PR$_3$), 154.3 (d, $^3J_{PC}$ = 6 Hz, C_{60}, next to *C*–H), 160.4 (d, $^2J_{PC}$ = 1.4 Hz, N=*C*(NMe$_2$)$_2$).
^{31}P NMR (162 MHz, 298 K, CD_2Cl_2): δ (ppm) = -20.7 (d, $^3J_{PH}$ = 32 Hz).
^{19}F NMR (376 MHz, 298 K, CD_2Cl_2): δ (ppm) = -132.9, -163.5, -167.4.

HRMS (ESI): m/z calculated for [$C_{75}H_{37}N_9P$]$^+$ [**2**]$^+$ 1094.2894, found 1094.2897.

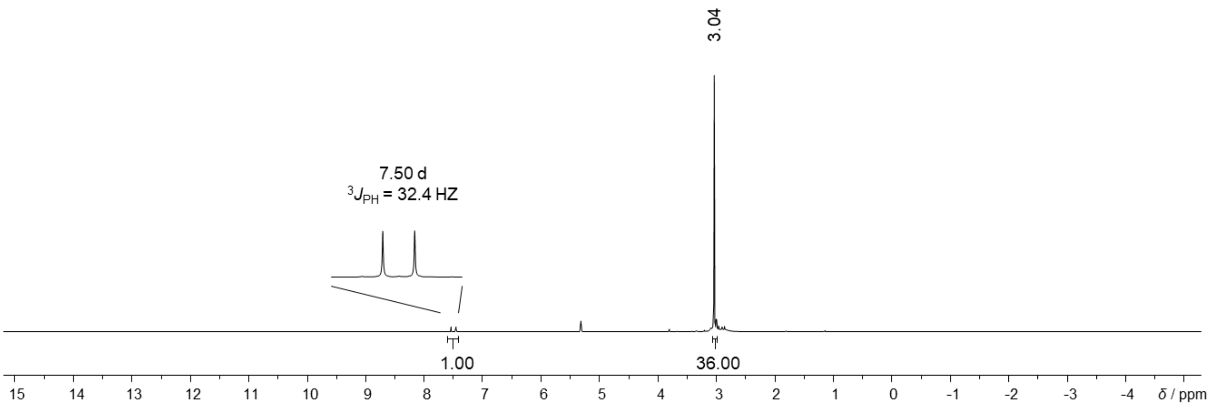

Figure S19: ^{1}H NMR spectrum (CD_2Cl_2, 400 MHz) of **2**.

.

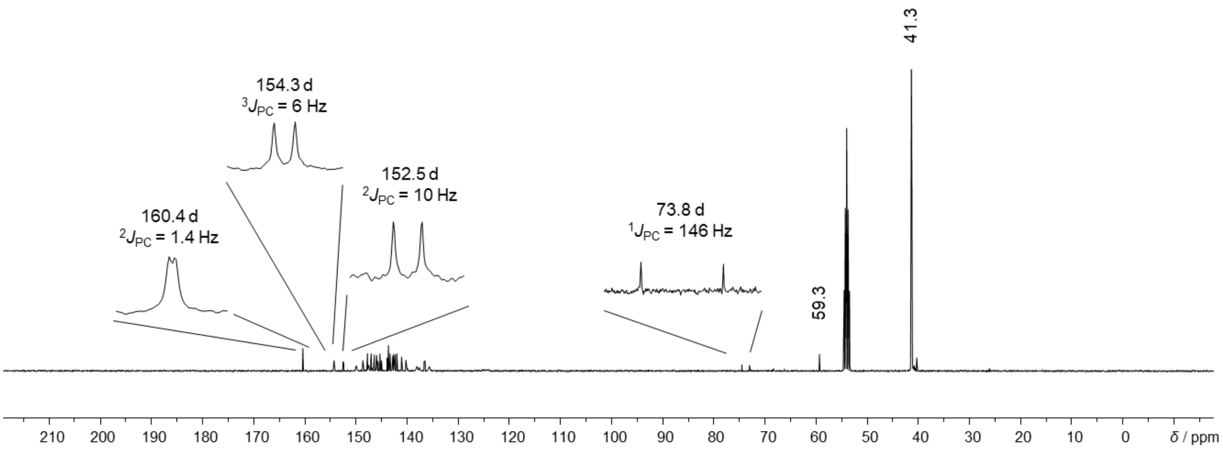

Figure S20: $^{13}C\{^{1}H\}$ NMR spectrum (CD_2Cl_2, 100 MHz) of **2**, for detailed zoom on the region of δ = 121 to 151 ppm see Figure S21: $^{13C\{1H\}}$ NMR spectrum (CD2Cl2, 100 MHz) of **2,** zoomed in from δ = 121 to 151 ppm, asterisks (*) mark resonances attributed to the [B(C6F5)$_4$] anion.Figure S21.

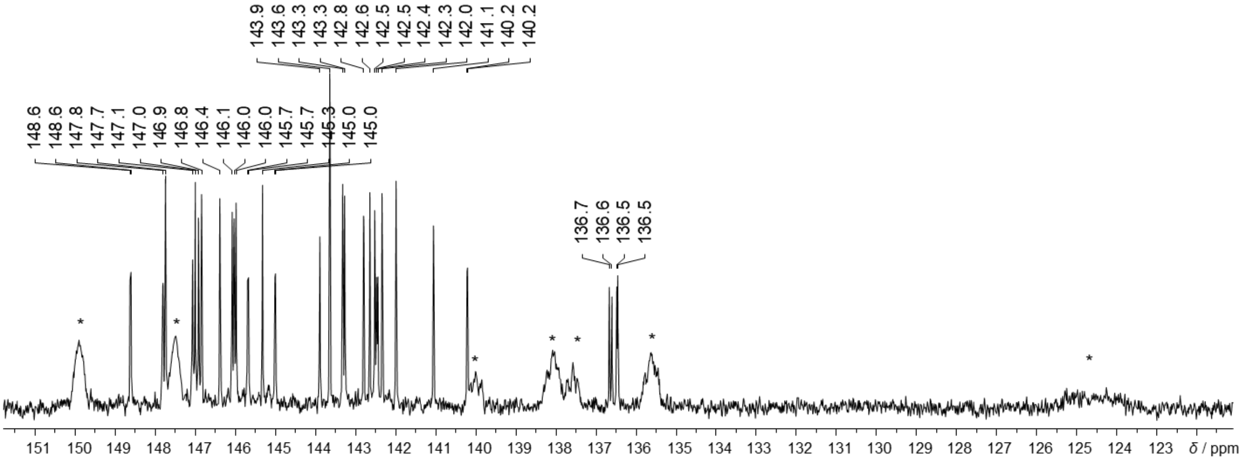

Figure S21: $^{13}C\{^{1}H\}$ NMR spectrum (CD_2Cl_2, 100 MHz) of **2**, zoomed in from δ = 121 to 151 ppm, asterisks (*) mark resonances attributed to the $[B(C_6F_5)_4]$ anion.

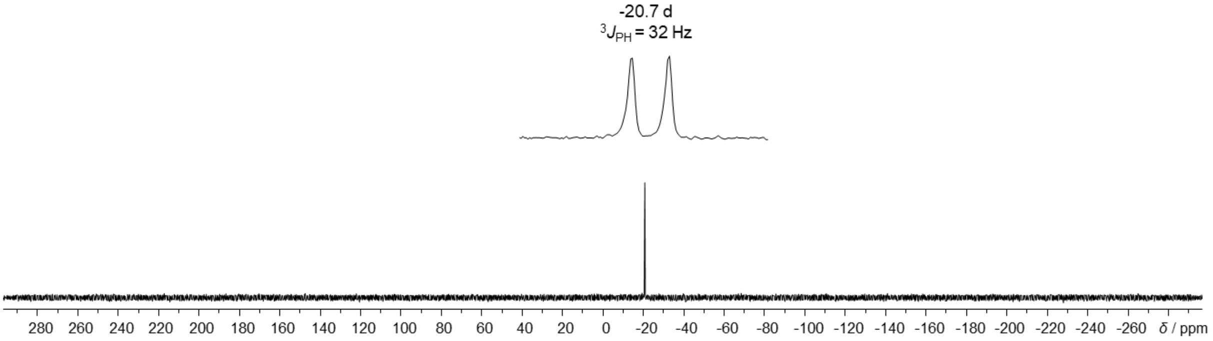

Figure S22: ^{31}P NMR spectrum (CD_2Cl_2, 162 MHz) of **2**.

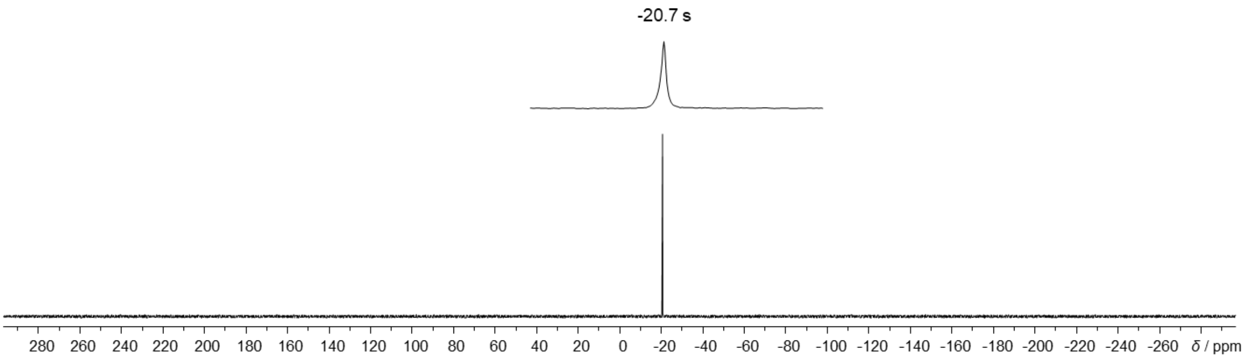

Figure S23: $^{31}P\{^{1}H\}$ NMR spectrum (CD_2Cl_2, 162 MHz) of **2**.

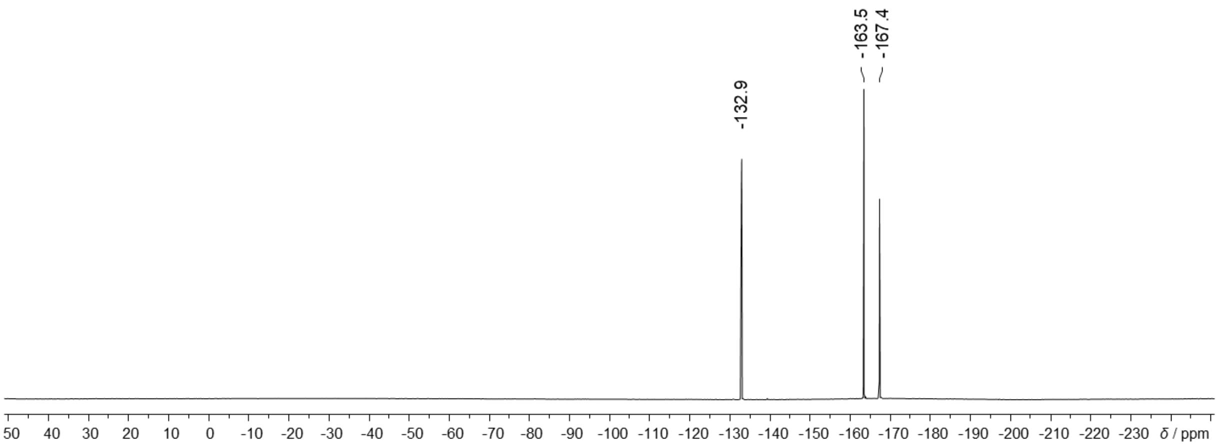

Figure S24: ^{19}F NMR spectrum (CD_2Cl_2, 376 MHz) of **2**.

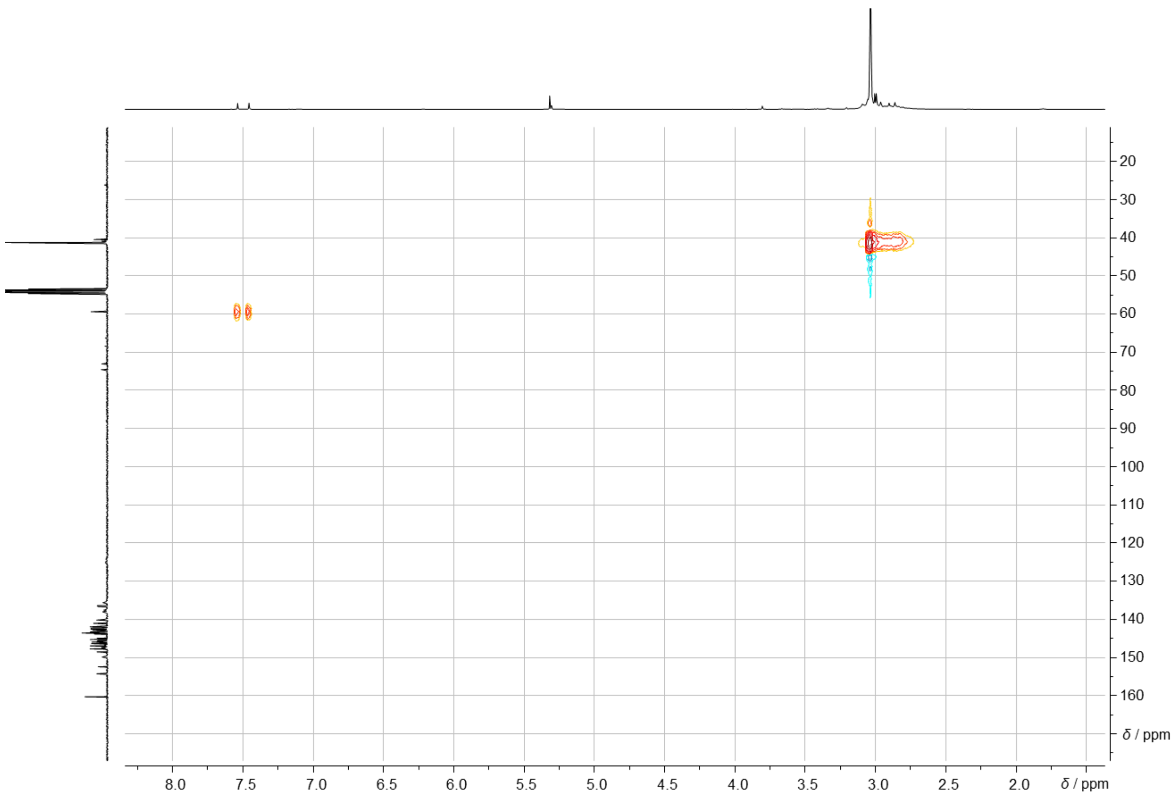

Figure S25: ^{1}H ^{13}C{^{1}H} HSQC NMR spectrum (CD_2Cl_2, 400 MHz, 100 MHz) of **2**.

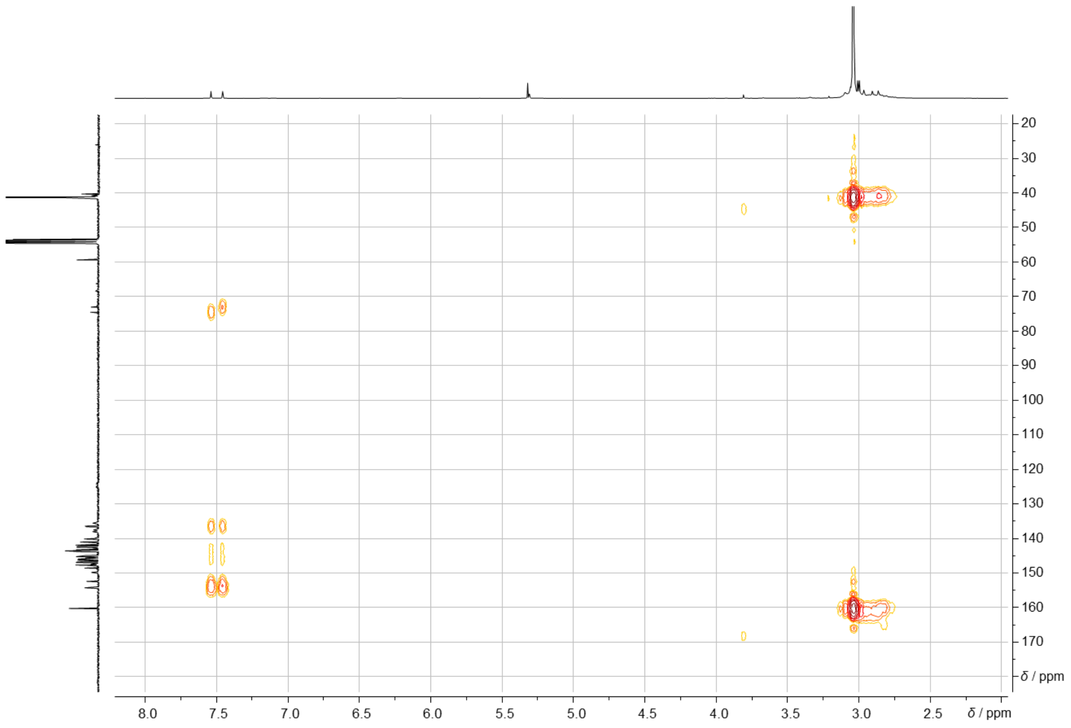

Figure S26: ^{1}H ^{13}C{^{1}H} HMBC NMR spectrum (CD_2Cl_2, 400 MHz, 100 MHz) of **2**.

1.5 Methylation of 1

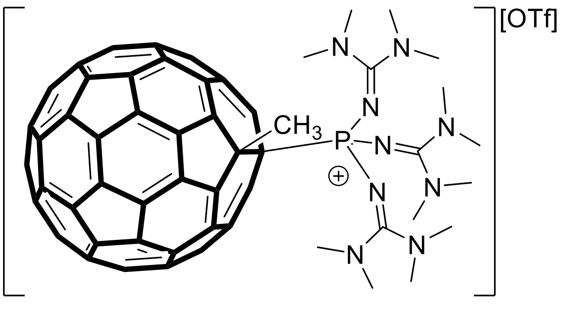

[**1**·CH_3][OTf]
3

A solution of methyl triflate (0.295 M in toluene, 0.1 mL, 0.030 mmol, 1 eq.) was added to a solution of **1** (32 mg, 0.030 mmol, 1 eq.) in dichloromethane (0.5 mL) in a PTFE-sealed NMR tube. After sonication of the NMR tube for 4 hours, complete conversion was indicated by ^{31}P NMR spectroscopy. The volatiles were removed *in vacuo*, which gave **3** as a brown solid.

^{1}H NMR (400 MHz, 298 K, CD_2Cl_2): δ (ppm) = 2.99 (s, 36 H, NC*H*$_3$), 2.83 (s, 3 H, C_{60}–C*H*$_3$).

^{13}C{^{1}H} NMR (100 MHz, 298 K, CD_2Cl_2): δ (ppm) = 29.0 (d, $^{3}J_{PC}$ = 12 Hz, C_{60}–*C*H$_3$), 41.3 (N*C*H$_3$), 56.0 (d, J_{PC} = 3.8 Hz, C_{60}, *C*–CH$_3$, long range coupling with C_{60}–C**H**$_3$), 69.5 (d, $^{1}J_{PC}$ = 144 Hz, C_{60}, *C*–PR$_3$), 120.0 (C_{60}), 123.2 (C_{60}), 137.6 (d, J_{PC} = 1.2 Hz, C_{60}), 141.5 (C_{60}), 142.4 (d, J_{PC} = 1.7 Hz, C_{60}), 142.5 (C_{60}), 142.8 (d, J_{PC} = 6 Hz, C_{60}), 142.9 (C_{60}), 143.0 (C_{60}), 143.1 (C_{60}), 143.2 (d, J_{PC} = 7 Hz, C_{60}), 143.5 (C_{60}), 143.6 (C_{60}), 143.8 (d, J_{PC} = 6 Hz, C_{60}), 143.9 (C_{60}), 144.1 (d, J_{PC} = 2.6 Hz, C_{60}), 144.4 (C_{60}), 144.5 (C_{60}), 144.5 (C_{60}), 144.6 (C_{60}), 144.7 (C_{60}), 145.0 (dd, J_{PC} = 4 Hz, J_{PC} = 10 Hz, C_{60}), 145.7-145.5 (m, C_{60}), 145.5-145.4 (m, C_{60}), 146.1 (d, J_{PC} = 10 Hz, C_{60}), 147.2 (d, J_{PC} = 12 Hz, C_{60}, long range coupling with C_{60}–C**H**$_3$), 147.3 (C_{60}), 147.4 (C_{60}), 147.5 (d, J_{PC} = 3.3 Hz, C_{60}), 147.6 (C_{60}), 147.8 (C_{60}), 148.0 (C_{60}), 149.2 (J_{PC} = 10 Hz, C_{60}), 149.3 (d, J_{PC} = 2.3 Hz, C_{60}), 149.4 (d, J_{PC} = 2.3 Hz, C_{60}), 149.6 (d, J_{PC} = 8 Hz, C_{60}), 150.2 (C_{60}), 153.2 (d, J_{PC} = 6 Hz, C_{60}, long range coupling with C_{60}–C**H**$_3$), 154.9 (d, J_{PC} = 10 Hz, C_{60}), 160.1 (d, $^{2}J_{PC}$ = 2.1 Hz, N=*C*(NMe$_2$)$_2$), 161.1 (d, J_{PC} = 3.2 Hz, C_{60}, long range coupling with C_{60}–C**H**$_3$).

^{31}P NMR (162 MHz, 298 K, CD_2Cl_2): δ (ppm) = -27.3.

^{19}F NMR (376 MHz, 298 K, CD_2Cl_2): δ (ppm) = -78.8.

HRMS (ESI): m/z calculated for [$C_{76}H_{39}N_9P$]$^+$ [**3**]$^+$ 1108.3061, found 1108.3048.

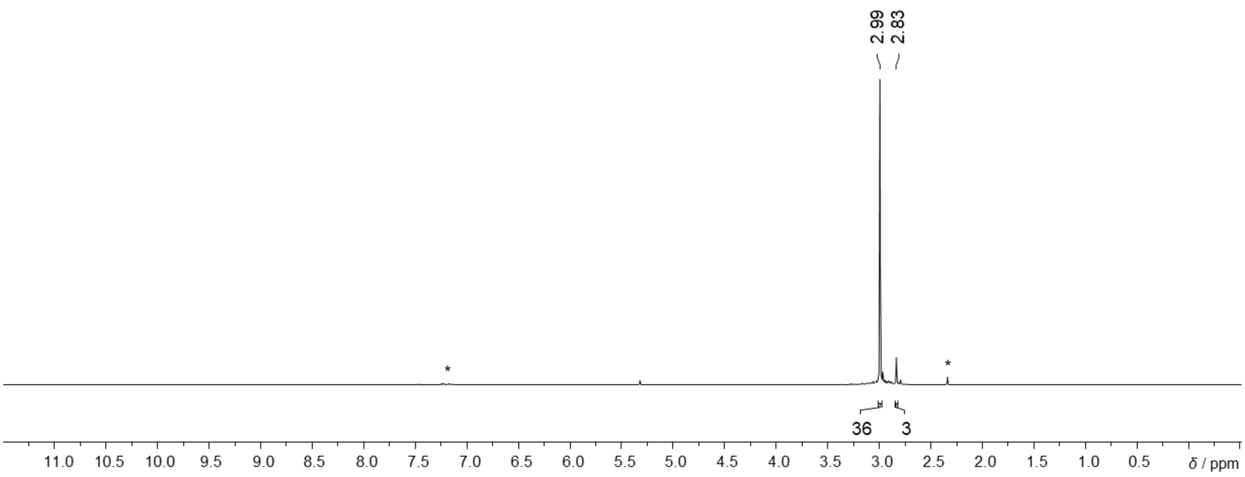

Figure S27: ^{1}H NMR spectrum (CD_2Cl_2, 400 MHz) of **3**, asterisks (*) mark residual toluene.

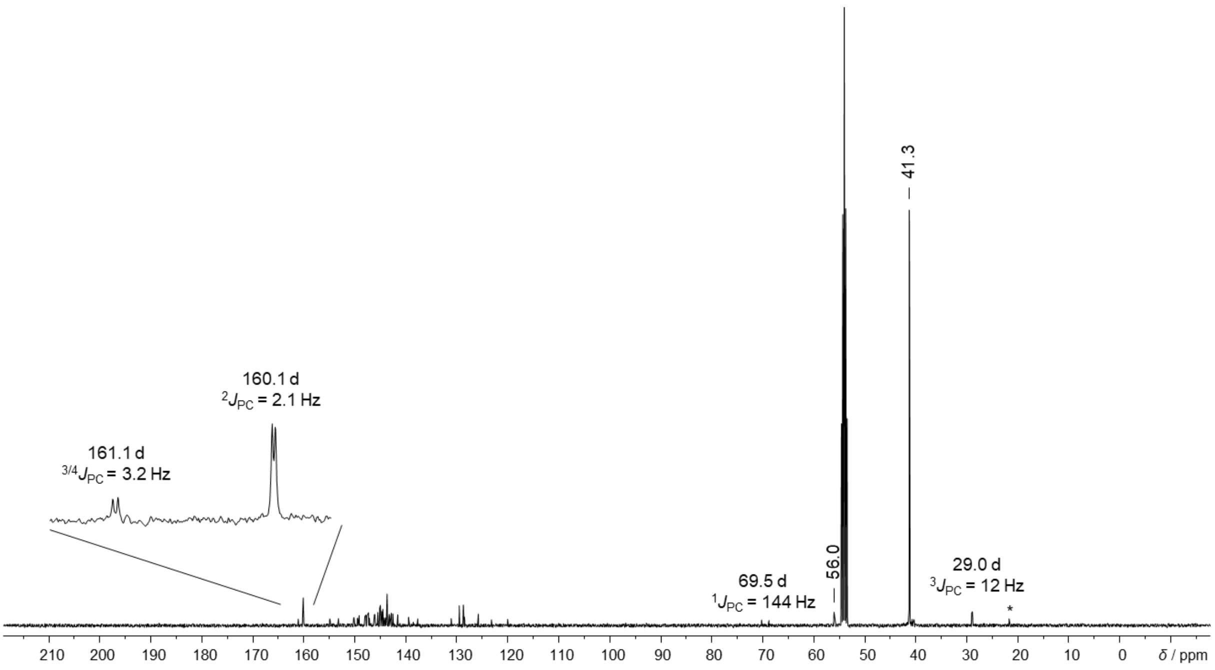

Figure S28: $^{13}C\{^{1}H\}$ NMR spectrum (CD_2Cl_2, 100 MHz) of **3**, for detailed zoom on the region of δ = 119 to 155 ppm see Figure S29, asterisk (*) marks residual toluene.

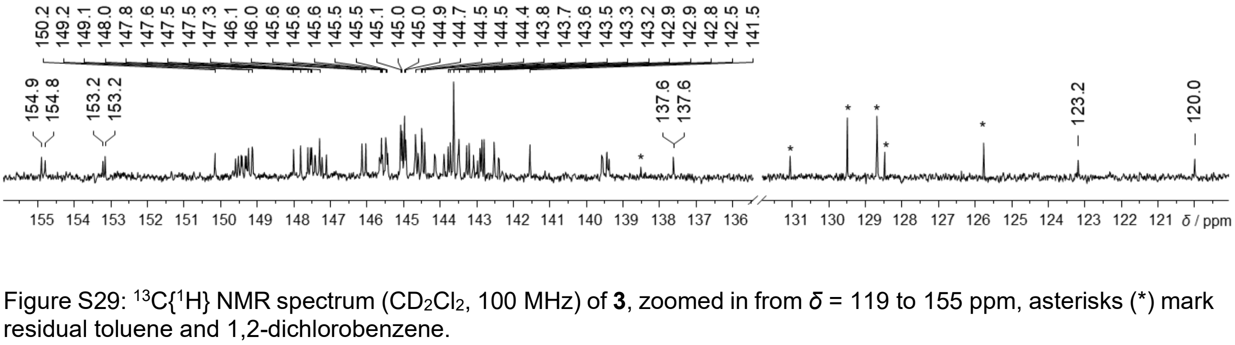

Figure S29: $^{13}C\{^{1}H\}$ NMR spectrum (CD_2Cl_2, 100 MHz) of **3**, zoomed in from δ = 119 to 155 ppm, asterisks (*) mark residual toluene and 1,2-dichlorobenzene.

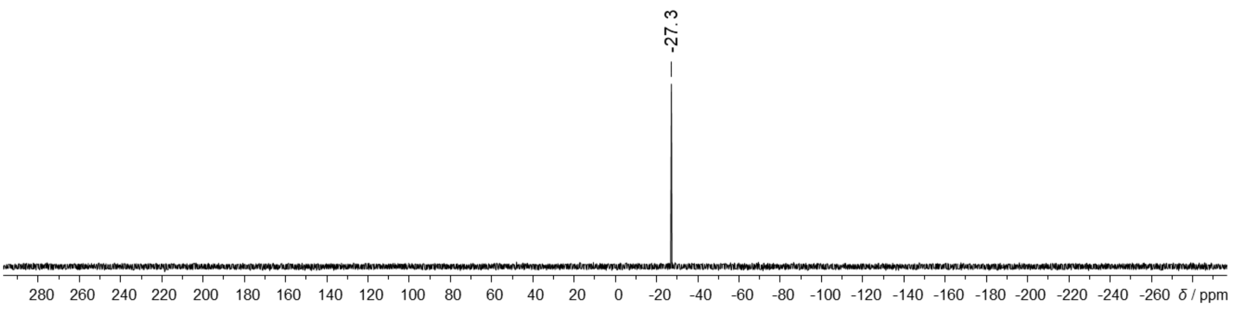

Figure S30: ^{31}P NMR spectrum (CD_2Cl_2, 162 MHz) of **3**.

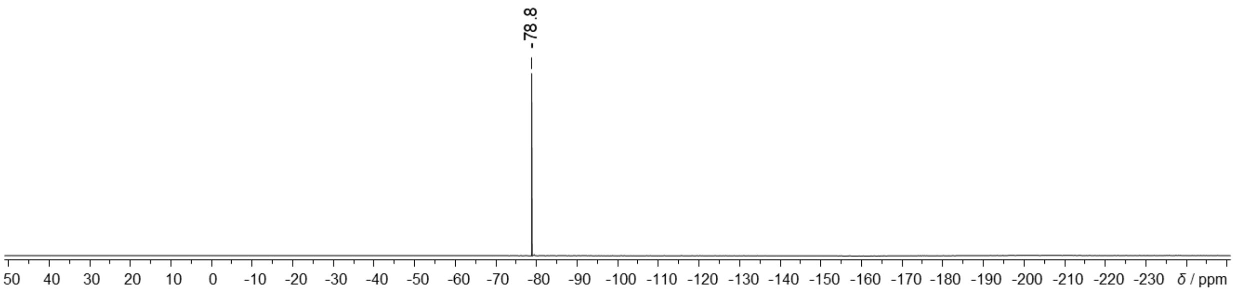

Figure S31: ^{19}F NMR spectrum (CD_2Cl_2, 376 MHz) of **3**.

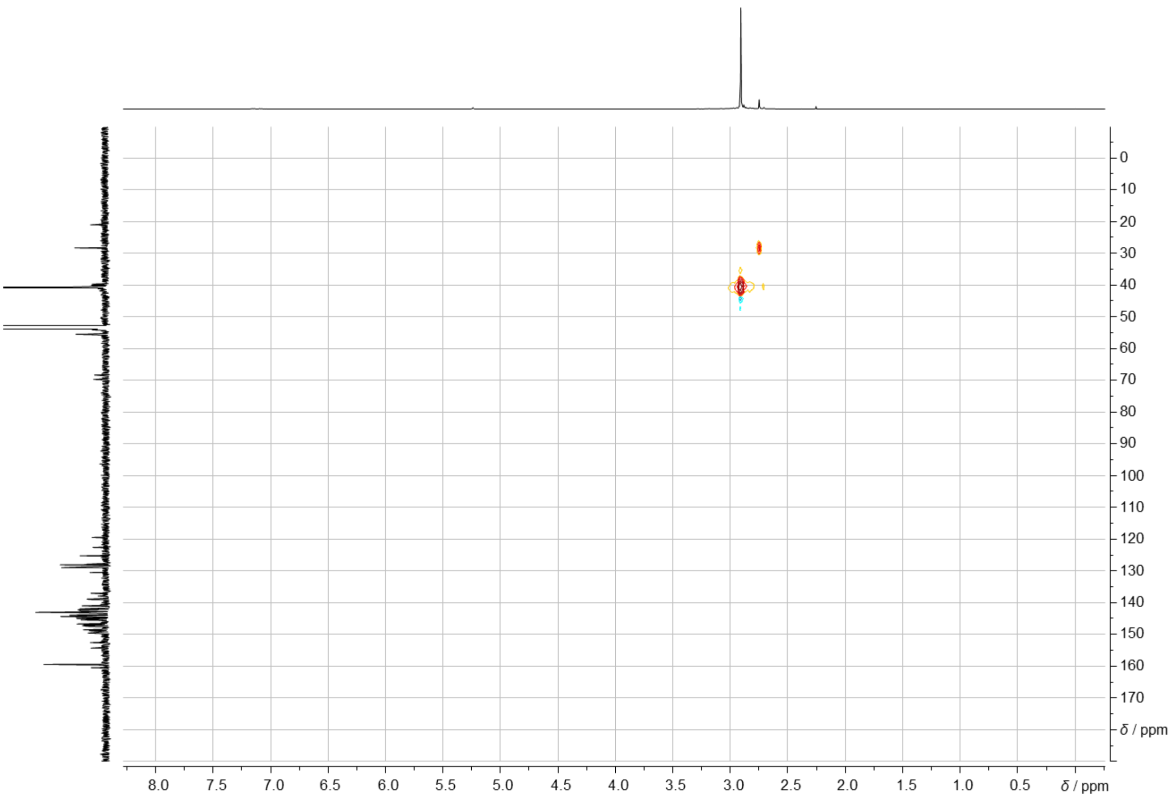

Figure S32: ^{1}H $^{13}C\{^{1}H\}$ HSQC NMR spectrum (CD_2Cl_2, 400 MHz, 100 MHz) of **3**.

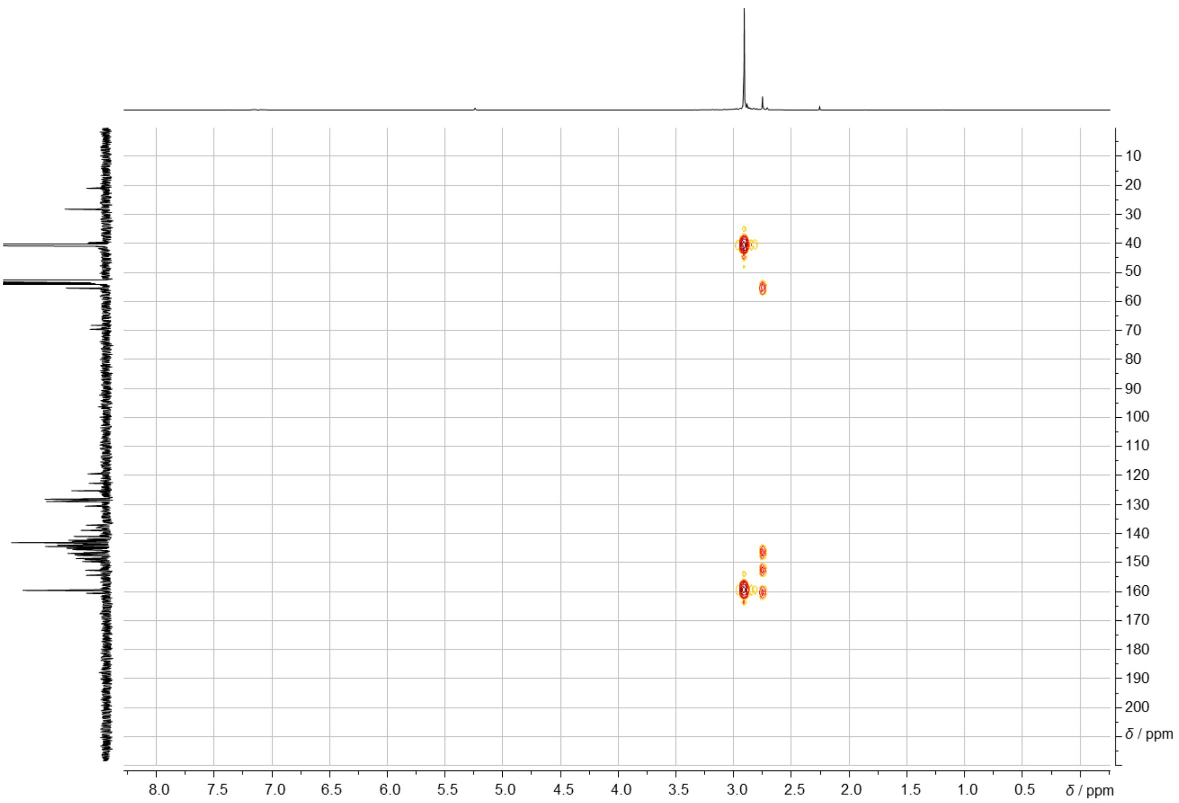

Figure S33: ^{1}H $^{13}C\{^{1}H\}$ HMBC NMR spectrum (CD_2Cl_2, 400 MHz, 100 MHz) of **3**.

1.6 Detailed Studies of the Reaction of C_{60} and $(tmg)_3P$

1.6.1 Stoichiometry Investigations

Phosphine $P(tmg)_3$ was reacted with C_{60} in different ratios to investigate whether more than one phosphine can bind to the fullerene. Details and outcome of the experiments are summarized in Table S1.

Table S1: Reactions of $P(tmg)_3$ with C_{60} in different ratios, solvents and under different conditions along with the resulting products identified by ^{31}P NMR spectroscopy; regioisomers $((tmg)_3P)_2C_{60}$ are abbreviated as **4** (see the explanation on the next page).

entry	starting materials	solvent	conditions	reaction products based on ^{31}P NMR spectroscopy (NMR yield)
1	1 eq. $P(tmg)_3$ + 1 eq. C_{60}	THF	r.t. hours	**4** + **1**
2			100 °C 16 h	**1** (>99 %)
3		DCB	r.t. minutes	**4** + **1**
4			r.t. 16 h	**1** (>99 %)
5	2 eq. $P(tmg)_3$ + 1 eq. C_{60}	THF	r.t. days or<br>100 °C 16 h	**4**
6		DCB	r.t. minutes	$P(tmg)_3$ + **4**
7			r.t. 16 h	**4** (>99 %)
8	3 eq. $P(tmg)_3$ + 1 eq. C_{60}	THF	r.t. days or<br>100 °C 16 h	$P(tmg)_3$ (50 %) + **4** (50 %)
9	6 eq. $P(tmg)_3$ + 1 eq. C_{60}	DCB	r.t. minutes	$P(tmg)_3$ (80 %) + **4** (20 %)
10	0.5 eq. $P(tmg)_3$<br>+ 1 eq. C_{60}	THF	r.t. minutes	**1** (>99 %)
11	0.2 eq. $P(tmg)_3$<br>+ 1 eq. C_{60}	DCB	r.t. minutes	**1** (>99 %)
12	1 eq. **1** + 1 eq. $P(tmg)_3$	THF	r.t. minutes or<br>100 °C 16 h or<br>365 nm hours	**4**
13	1 eq. **4**+ 1 eq. C_{60}	THF	100 °C 16 h	**1** (>99 %)

The stoichiometric experiments summarized in Table S1 reveal that **1** is formed when stoichiometric or substoichiometric amounts of $P(tmg)_3$ are reacted with C_{60}, provided that the reaction time is sufficient to achieve equilibration (see Entry 2, 4, 10-11). Notably, regardless of the stoichiometry, compound **4** is initially formed (see Entry 1+3). This is attributed to the low solubility of buckminsterfullerene C_{60}, which results in an excess of phosphine in solution.

When 2 equivalents of $P(tmg)_3$ are used, a mixture of regioisomers is obtained (see Entry 5-7, 12; find NMR data on **4** in 1.6.2). Adding C_{60} to **4** leads to the formation of **1** (see Entry 13), confirming the reversibility of the adduct formation.

If more than 2 equivalents of $P(tmg)_3$ are employed, no additional phosphine binds to **4**, as evidenced by the presence of free phosphine in solution (see Entry 8-9).

1.6.2 NMR spectroscopic investigations of 4

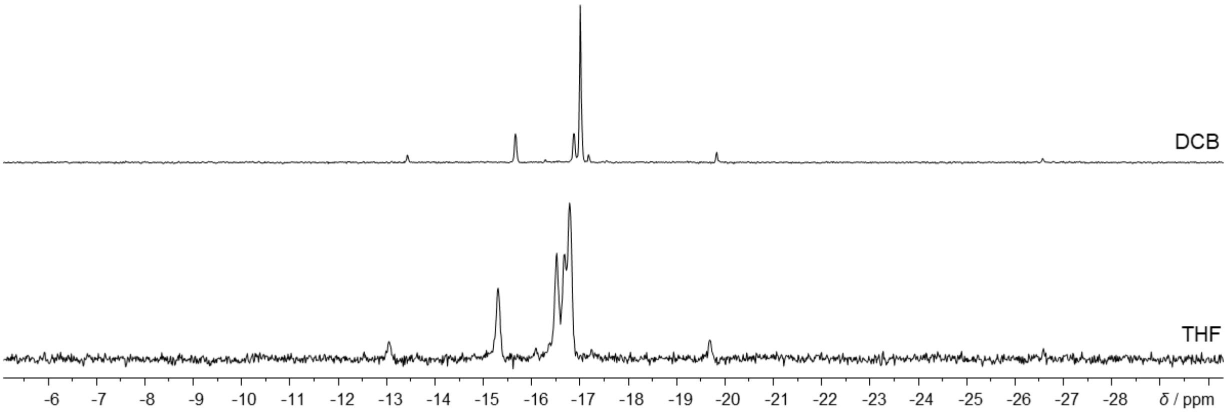

Figure S34: ^{31}P NMR spectra (162 MHz) of **4**; the main resonance in DCB results from isochrone nuclei.

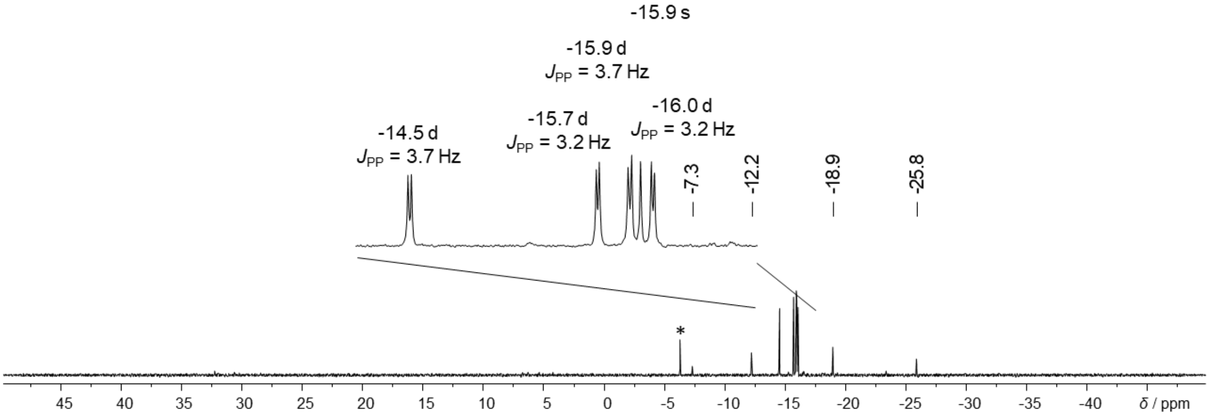

Figure S35: ^{31}P{^{1}H} NMR spectrum (THF, 162 MHz, 30 °C) of **4**, asterisk marks phosphine oxide ((tmg)$_3$P=O).

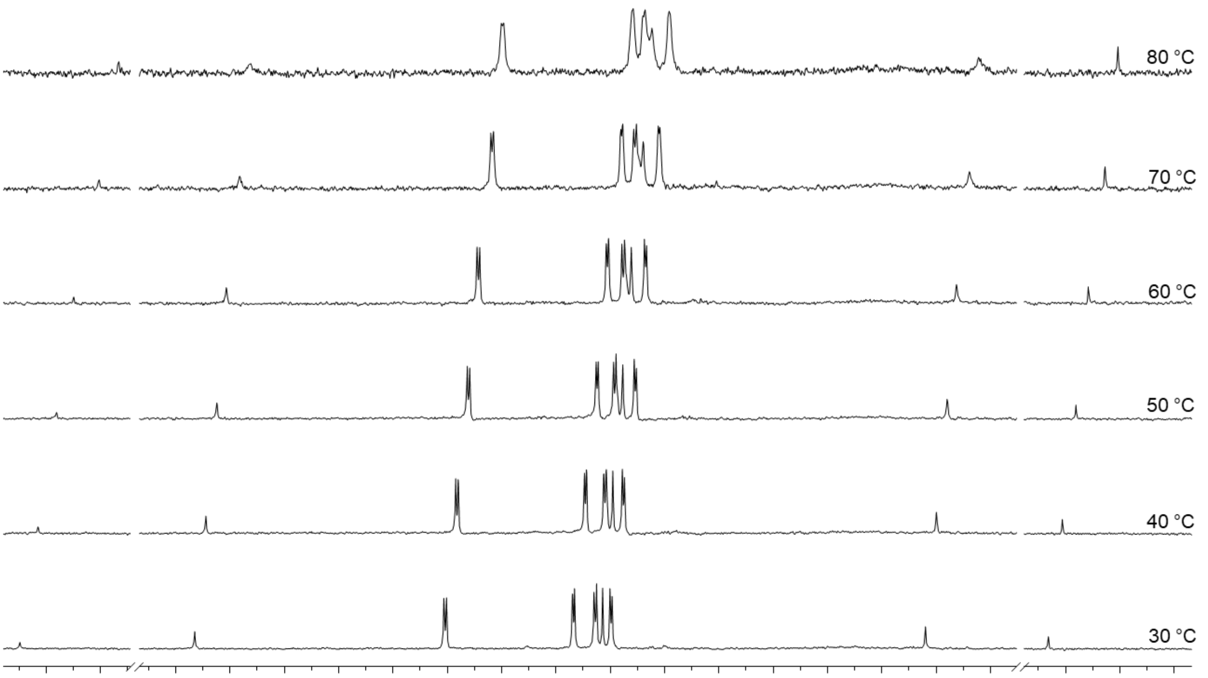

Figure S36: ^{31}P{^{1}H} NMR spectra (THF, 162 MHz) of **4** at temperatures from 30 to 80 °C.

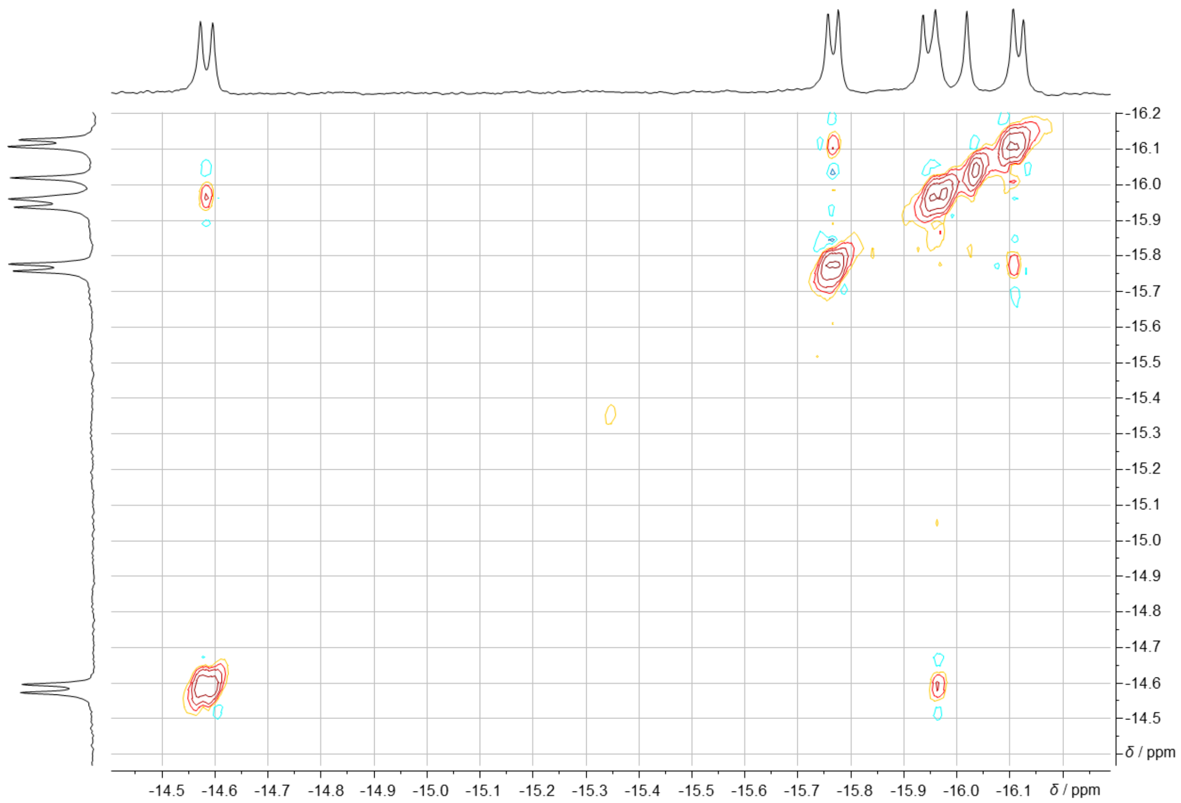

Figure S37: ^{31}P ^{31}P COSY NMR spectrum (THF, 162 MHz, 162 MHz, 30 °C) of **4**, zoomed in the area of doublets.

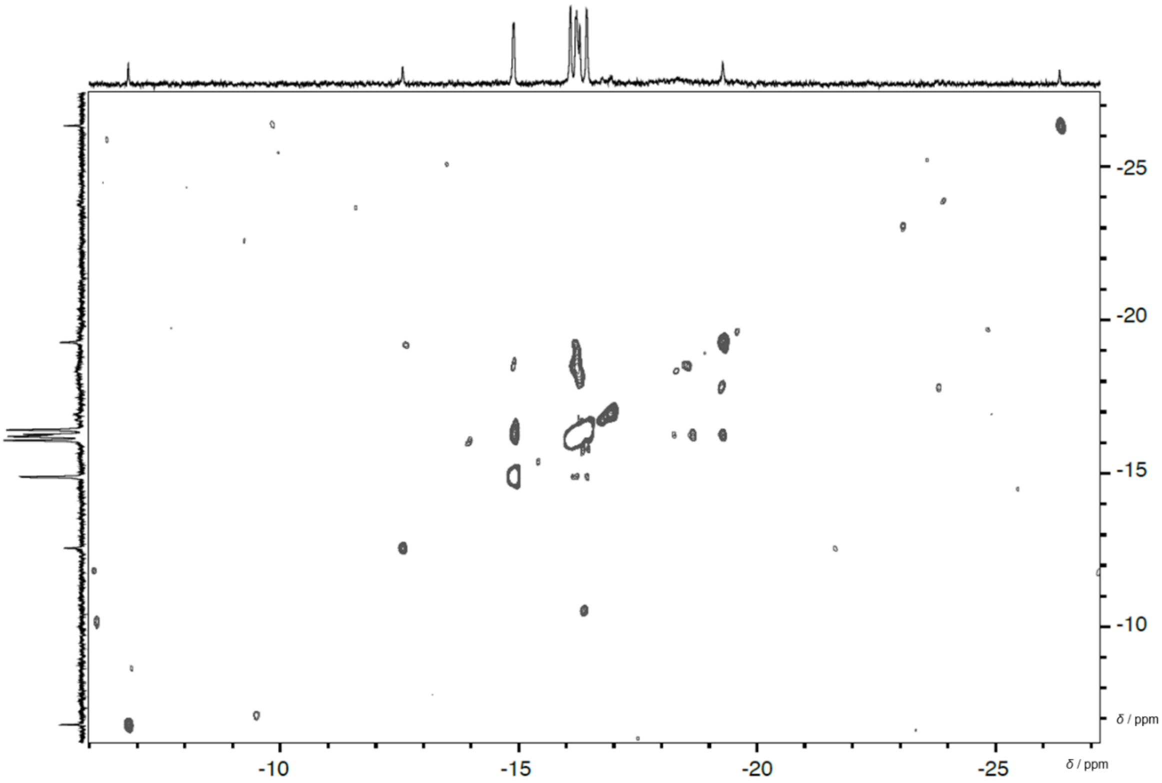

Figure S38: ^{31}P ^{31}P NOESY/EXSY NMR spectrum (THF, 162 MHz, 162 MHz, 70 °C, mixing time 150 msec) of **4**.

1.6.3 Protonation of 4

Lutidinium triflate (17.5 mg, 0.068 mmol, 2 eq.) was added to a suspension of **4** (50 mg, 0.034 mmol, 1 eq.) in DCB (0.5 mL). Upon addition, the green color of the reaction mixture changed to brown. The reaction mixture was analyzed by ^{31}P NMR and $^{31}P\{^1H\}$ NMR spectroscopy (Figure S39).

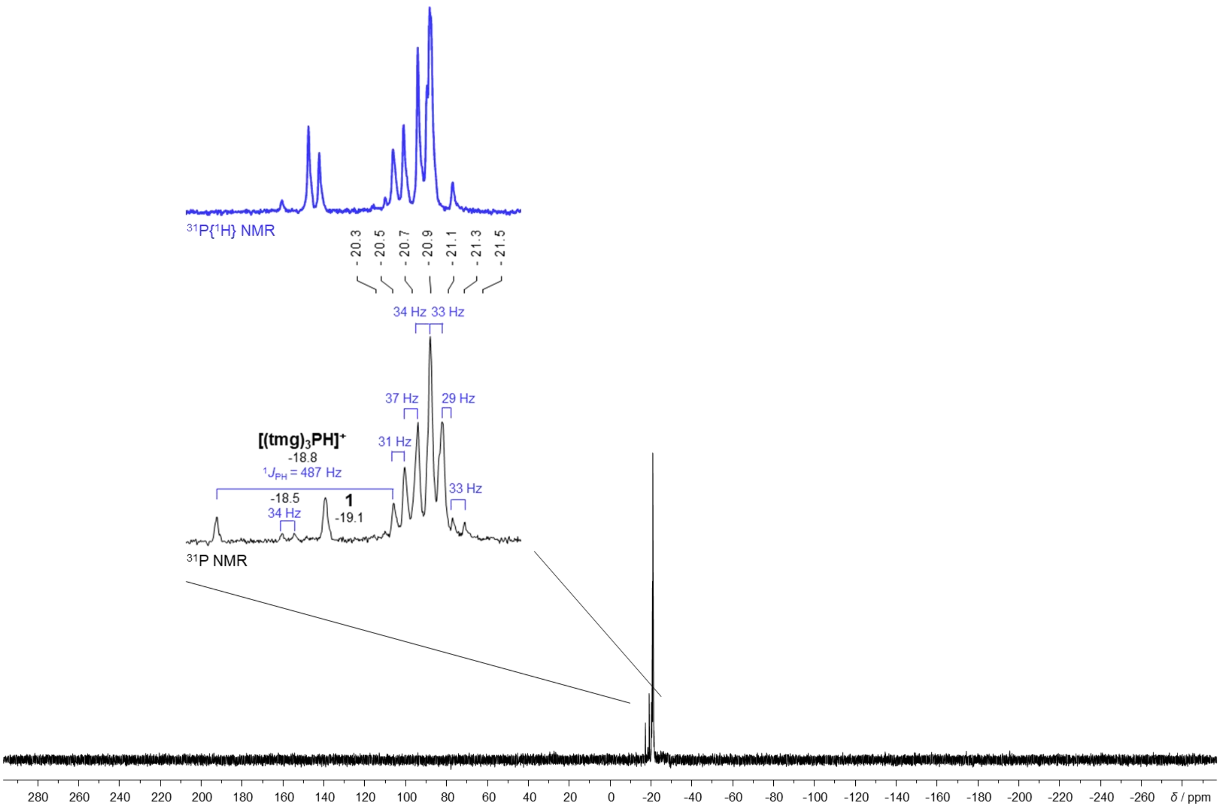

Figure S39: ^{31}P NMR (black) and $^{31}P\{^1H\}$ NMR (blue) spectra of the reaction mixture (DCB, 162 MHz).

The ^{31}P NMR resonances of the regioisomers $((tmg)_3P)_2C_{60}$ (**4**) disappeared upon addition of the proton source, and new resonances emerged as doublets with $^3J_{PH}$ coupling constants ranging from 29 to 37 Hz. Based on the similarity of the ^{31}P chemical shifts and the corresponding indicative $^3J_{PH}$ coupling constants, these resonances are assigned to a mixture of regioisomers $[((tmg)_3P)_2C_{60}H_2]^+$ (**5**). Notably, small amounts of the $(tmg)_3P–C_{60}$ monoadduct (**1**, s, δ = -19.1 ppm) and the protonated phosphine $[(tmg)_3PH]^+$ (d, δ = -18.8 ppm, $^1J_{PH}$ = 487 Hz)[2] were detected, resulting from $P–C_{60}$ bond cleavage and protonation of the free phosphine.

Attempts to detect the cationic fullerene derivatives using ESI-HRMS spectrometry were unsuccessful.

1.6.4 Reversibility Study: Reaction of 1 and 4 with gray selenium

A) Reaction of compound 4 with gray selenium at ambient temperature.

Gray selenium (4 mg, 0.05 mmol, >2 eq.) was added to a suspension of the regioisomers $((tmg)_3P)_2C_{60}$ (**4**) (20 mg, 0.014 mmol, 1 eq.) in DCB (0.5 mL) in a PTFE-sealed NMR tube. The green reaction mixture was analyzed by ^{31}P NMR spectroscopy after storing it for 30 minutes at ambient temperature and after additional sonication for 30 minutes at ambient temperature (Figure S40). After 30 minutes, the spectrum shows resonances for the known phosphine selenide $(tmg)_3PSe$ (δ = 10.8 ppm)[2], the regioisomers of disubstituted fullerenes **4**, and the monoadduct **1** (δ = -19.0 ppm). Following sonication, the spectrum indicates complete conversion to the phosphine selenide and **1**, demonstrating the reversibility of the P–C_{60} bond formation in $((tmg)_3P)_2C_{60}$ (**4**), which undergoes heterolytic cleavage at ambient temperature.

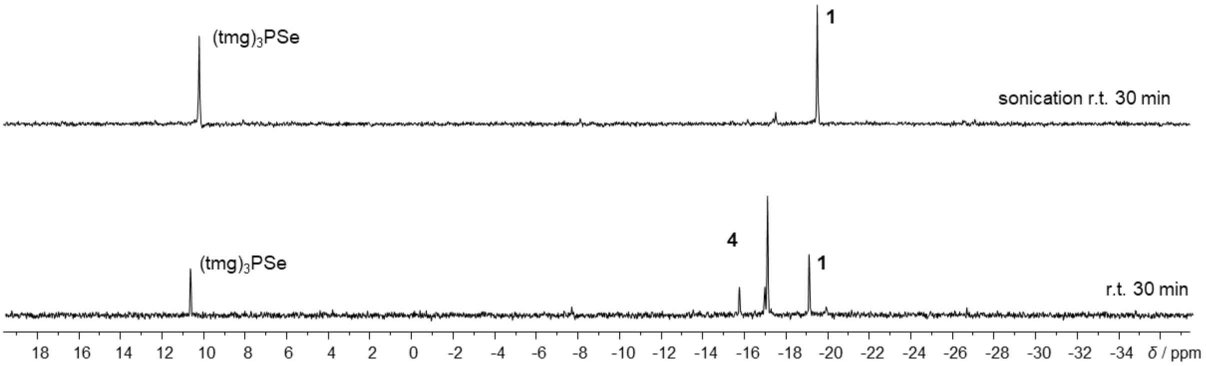

Figure S40: ^{31}P NMR spectrum (DCB, 162 MHz) of the reaction mixture (no further resonances present between +400 and -400 ppm).

A) Reaction of compound 1 with gray selenium at ambient temperature.

Gray selenium (4 mg, 0.05 mmol, >1 eq.) was added to a suspension of compound **1** (20 mg, 0.018 mmol, 1 eq.) in DCB (0.5 mL) in a PTFE-sealed NMR tube. The green reaction mixture was analyzed by ^{31}P NMR spectroscopy after storing it for 30 minutes at ambient temperature and after additional sonication for 30 minutes at ambient temperature, showing no reaction at ambient temperature (Figure S41).

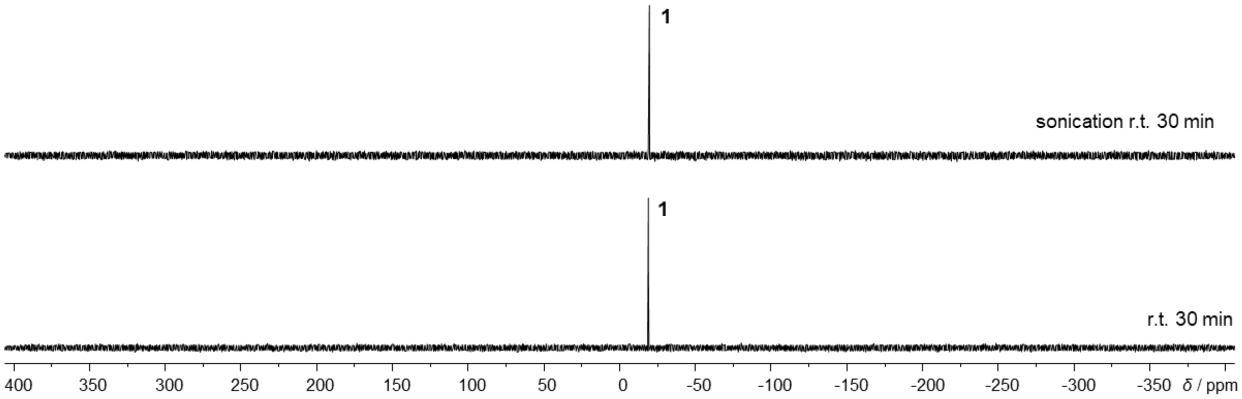

Figure S41: ^{31}P NMR spectrum (DCB, 162 MHz) of the mixture of **1** and Se.

C) Reaction of compound 1 with gray selenium at 100 °C.

Gray selenium (4 mg, 0.05 mmol, >1 eq.) was added to a suspension of **1** (20 mg, 0.018 mmol, 1 eq.) in THF (0.5 mL) in a PTFE-sealed NMR tube. The green reaction mixture was analyzed by ^{31}P NMR spectroscopy directly after the addition, and after heating the NMR tube at 100 °C for 16 hours (Figure S42). During heating, the reaction mixture turned brown and a black precipitate formed. The ^{31}P NMR spectrum shows the resonances for the known phosphine selenide $(tmg)_3PSe$[2] (δ = 12.9 ppm, $^{1}J_{PSe}$ = 709 Hz), demonstrating the reversibility of the adduct formation at elevated temperature.

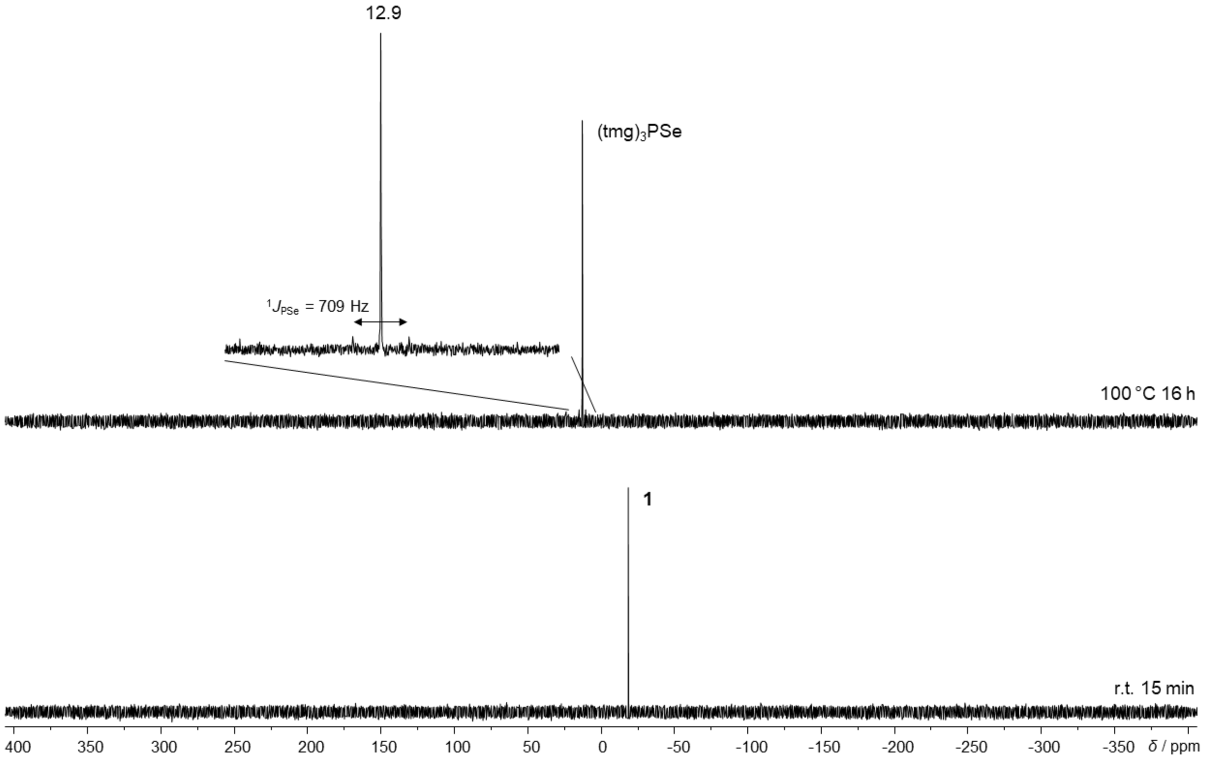

Figure S42: ^{31}P NMR spectrum (THF, 162 MHz) of the mixture of **1** and Se.

1.6.5 EPR studies

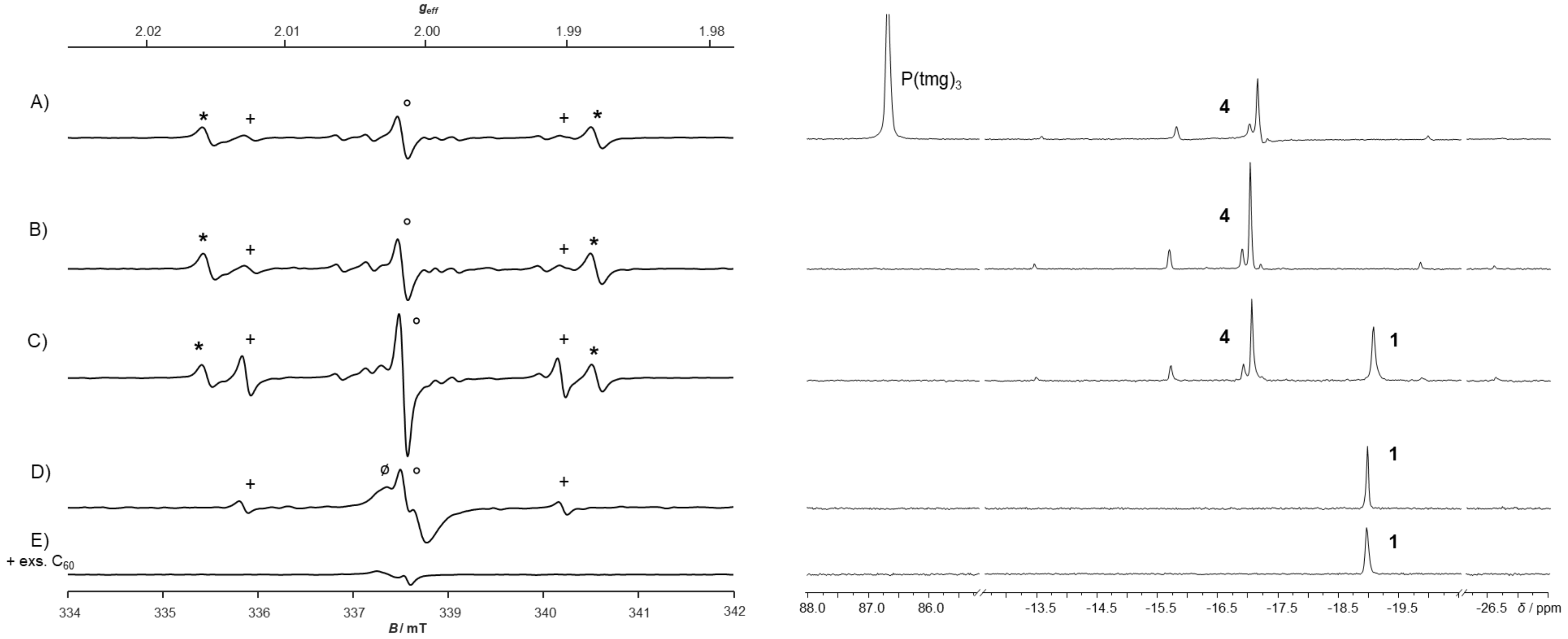

Figure S43: X-band EPR spectra (left, DCB) and ^{31}P NMR spectra (right, DCB, 162 MHz) of reaction mixtures containing C_{60} and $P(tmg)_3$ in different stoichiometric ratios. Concentrations of the solution range from 10 - 70·10^{-3} mol/L. Reaction conditions (time is the stirring time before samples were taken for spectroscopic analysis): A) 6 eq. $P(tmg)_3$ + 1 eq. C_{60}, stirring for 5 minutes t 21 °C; B) 2 eq. $P(tmg)_3$ + 1 eq. C_{60}, stirring for 16 h at 21 °C; C) 1 eq. $P(tmg)_3$ + 1 eq. C_{60}, stirring for 5 minutes at 21 °C; D) 1 eq. $P(tmg)_3$ + 1 eq. C_{60}, stirring for 16 h at 21 °C, E) **1** + excess C_{60}, stirring for 16 h at 21 °C.

X-band EPR measurements were performed on solutions containing C_{60} and $P(tmg)_3$ with varying stoichiometries (Figure S43, left). The solutions were prepared by adding DCB to a mixture of C_{60} and $P(tmp)_3$, stirring at ambient temperature for a set time. Due to the low solubility of C_{60}, the exact ratio of C_{60} to $P(tmg)_3$ in solution is unclear. Therefore, the solutions were also analyzed by ^{31}P NMR spectroscopy (Figure S43, right).

Control experiments with DCB solutions of either C_{60} or $P(tmg)_3$ alone show that both are EPR silent. Similarly, no radical species were detected in solutions containing an excess of C_{60} (Figure S43 E). However, in solutions with more than one equivalent of $P(tmg)_3$ (Figure S43 A-C), several paramagnetic species were observed. Two signals exhibit hyperfine coupling of a_{iso} = 131 MHz (*****, g_{iso} = 2.0015) and a_{iso} = 106 MHz (**+**, g_{iso} = 2.0014), which is in the expected range for phosphorus centered radicals[7]. Since their intensity ratio depends on the stoichiometry and cannot be independently correlated to any other signal, they are assigned to two distinct phosphorus-centered radical species. Additionally, a signal at g = 2.0015 (**o**, $a_{iso}(^{13}C)$ = 11.6 MHz) is assigned to the $C_{60}^{\bullet -}$ radical anion. A broader signal **ø** (Figure S43 D) suggests the presence of an additional carbon-centered radical.

These experiments indicate that paramagnetic species form when more than one equivalent of $P(tmg)_3$ is present, leading to the formation of bisphosphine-fullerene adducts **4**. This suggests that single-electron transfer (SET) processes or homolytic P–C bond dissociation occurs to some extent. Given the lower stability of the bisphosphine adducts (**4**) compared to the monoadduct (**1**), homolytic P–C bond dissociation is likely more prevalent for **4**. Furthermore, an electron transfer from the electron-rich fullerene core of regioisomers **4** to either **1** or C_{60} can occur, potentially leading to the formation of the $C_{60}^{\bullet -}$ radical anion and the corresponding radical cations $[(tmg)_3PC_{60}]^{\bullet +}$ and $[((tmg)_3P)_2C_{60}]^{\bullet +}$.

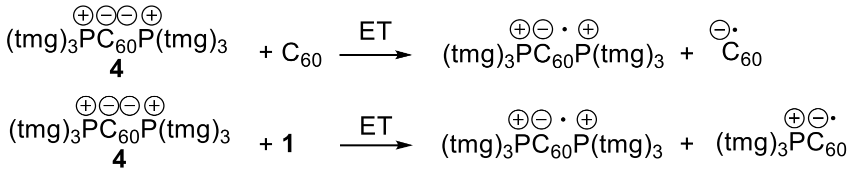

Figure S44: Suggested electron transfer (ET) reactions.

2 X-ray Crystallography

2.1 Crystal Structure Data of Compound 1

Single dark green block-shaped crystals were obtained from a concentrated THF solution.

A suitable crystal with dimensions 0.16 × 0.11 × 0.09 mm^3 was selected and mounted on a Bruker D8 QUEST PHOTON III diffractometer. The crystal was kept at a steady *T* = 153.00 K during data collection. The structure was solved with the ShelXT[8] solution program using dual methods and by using Olex2 1.5-alpha[9] as the graphical interface. The model was refined with ShelXL[10] 2018/3 using full matrix least squares minimisation on F^2.

A Single crystal X-ray structure analysis revealed that **1** crystallizes in the orthorhombic space group *Pnma*. The asymmetric unit contains 0.5 x $C_{79}H_{44}N_9OP$ (0.5 x (one molecule of **1** and a THF molecule)). The fullerene unit is disordered over four positions (occupancy 0.2:0.2:0.3:0.3), the THF molecule is disordered over two positions (occupancy 0.5:0.5) and additionally the CH_3 groups at the nitrogen atoms N3 and N2 are close to a special position (mirror plane through the two nitrogen atoms N2 and N3) and therefore disordered over two positions (occupancy of C65 and C62 0.5:0.5, occupancy of the H atoms at C63 and C64 0.5:0.5).

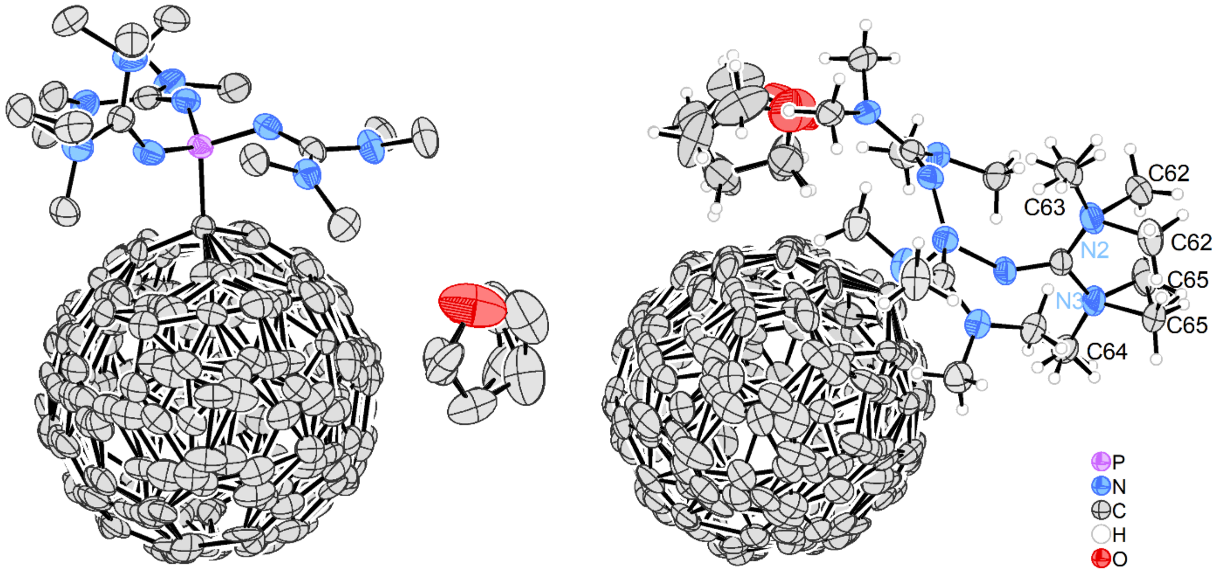

Figure S45: Two different views of the molecular structure of $C_{79}H_{44}N_9OP$ with thermal ellipsoid plots at the 50% level of probability.

Table S2: Crystal data and structure refinement for **1**.

CCDC number	2411442	Z	4
Formula	$C_{79}H_{44}N_9OP$	Z'	0.5
$D_{calc.}$/ g cm^{-3}	1.510	Wavelength/Å	0.71073
μ/mm^{-1}	0.121	Radiation type	MoK$_a$
Formula Weight	1166.20	θ_{min}/°	2.142
Colour	dark green	θ_{max}/°	28.319
Shape	block-shaped	Measured Refl's.	114077
Size/mm^3	0.16×0.11×0.09	Indep't Refl's	6641
T/K	153.00	Refl's I≥2 s(I)	5130
Crystal System	orthorhombic	R_{int}	0.0506
Space Group	*Pnma*	Parameters	1254
a/Å	15.6389(4)	Restraints	1885
b/Å	13.6916(5)	Largest Peak	0.638
c/Å	23.9526(9)	Deepest Hole	-0.478
α/°	90	GooF	1.034
β/°	90	wR_2 (all data)	0.1673
γ/°	90	wR_2	0.1530
V/Å^3	5128.8(3)	R_1 (all data)	0.0725
		R_1	0.0553

Crystallographic data have been deposited with the Cambridge Crystallographic Data Centre as supplementary publication no. CCDC - 2411442 (**1**). These data can be obtained free of charge via https://www.ccdc.cam.ac.uk/structures (or from the CCDC, 12 Union Road, Cambridge CB2 1EZ, UK; fax: (+44) 1223-336-033; or deposit@ccdc.cam.ac.uk).

3 Computational Details

3.1 General

All geometry optimizations as well as frequency, transition state (TS) calculations and the NBO analysis were performed using the Gaussian16 program package.[11] Cartesian coordinates were extracted from the crystallographic data of **1** and the structure was preliminarily refined using the xtb (v. 6.3.2) program.[12] The regioisomers of **4** were drawn using Avogadro[13] (v. 4.8.6) and preliminarily refined using the xtb (v. 6.3.2) program as well. Starting from the preliminarily refined structures, geometry optimizations were performed using the B3LYP[14,15] functional and 6-31G(d,p)[16–20] basis set. Frequency calculations of the optimized structure confirmed the stationary point as minimum through the absence of imaginary frequencies. The influence of solvent effects in tetrahydrofuran (relative permittivity $\varepsilon = 7.4257$) was accounted for using the implicit solvent model density (SMD) approach.[21] For **1**, subsequent NBO analysis (implemented as NBO 3.0 in the Gaussian16 suite) was performed using the optimized geometry. To account for dispersion effects, Grimme's dispersion correction with the Becke-Johnson damping (GD3BJ)[22] was applied in all calculations.

The Chemcraft software (v. 1.8)[23] was employed to render and depict molecules as well as molecular and natural bond orbitals.

^{31}P NMR chemical shifts were calculated following a similar approach as recently reported by Hersh.[24] All geometry optimizations and frequency calculations were performed using the Gaussian16 program package[11] using the B3LYP[14] functional and 6-31G(d,p)[16–20] basis set. Frequency calculations of the optimized structure confirmed the stationary point as minimum through the absence of imaginary frequencies. Starting from the optimized geometries, subsequent NMR calculations were performed using the

i) B3LYP functional and 6-31G(d,p) basis set,

ii) M06-2X[25] functional and 6-31G(d,p) basis set or the

iii) M06-2X functional and 6-311+G(2d,p)[17,26] basis set.

Calculations of the NMR shielding tensor were carried out following the Gauge-Independent Atomic Orbital (GIAO) method.[21,27–31]

The influence of solvent effects in acetonitrile (relative permittivity $\varepsilon = 35.688$), benzene (relative permittivity $\varepsilon = 2.2706$), carbon tetrachloride (relative permittivity $\varepsilon=2.228$), chloroform (relative permittivity $\varepsilon = 4.7113$), tetrahydrofuran (relative permittivity $\varepsilon = 7.4257$), and water (relative permittivity $\varepsilon = 78.3553$) was accounted for using the implicit solvent model density (SMD) approach.[21]

To account for dispersion effects, Grimme's dispersion correction with the Becke-Johnson damping (GD3BJ)[22] was used (not available for the M06-2X functional group reported by Truhlar and Zhao[25]).

3.2 Orbitals of 1

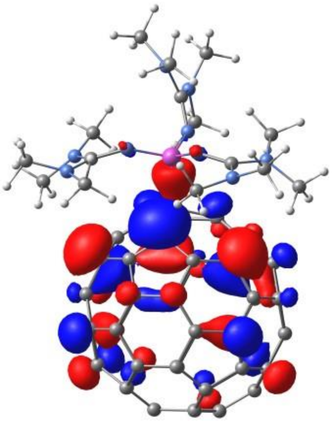

HOMO (–4.083 eV)

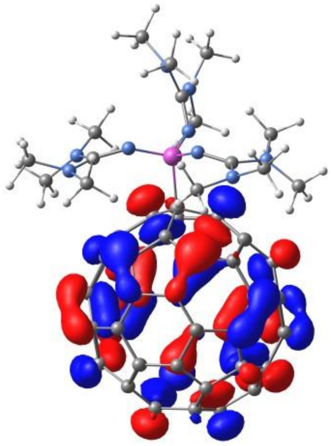

HOMO–1 (–5.195 eV)

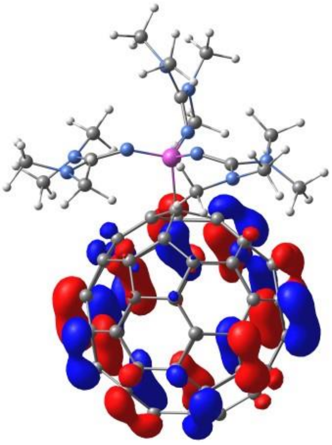

HOMO–2 (–5.269 eV)

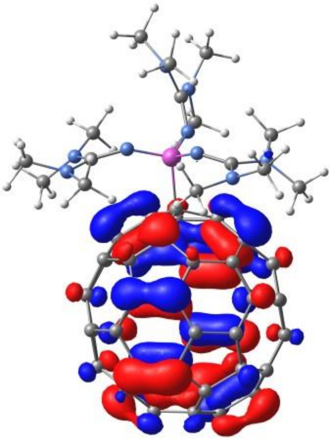

HOMO–3 (–5.289 eV)

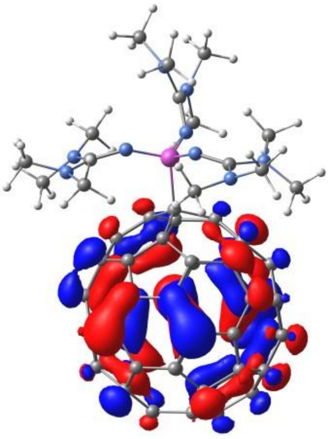

HOMO–4 (–5.325 eV)

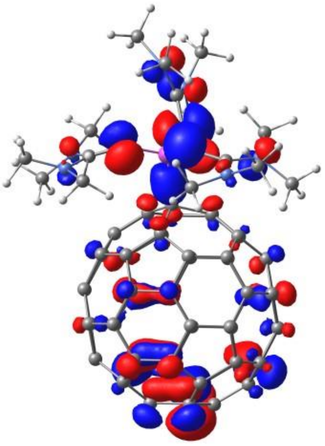

HOMO–5 (–5.727 eV)

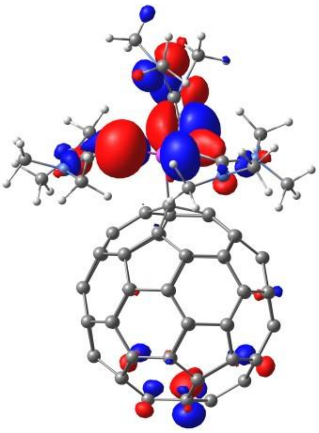

HOMO–6 (–5.824 eV)

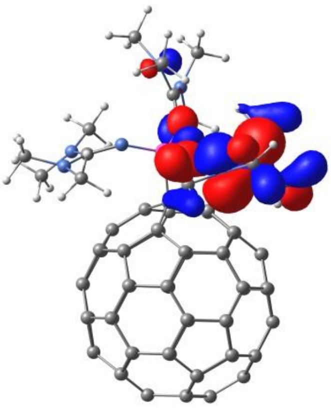

HOMO–7 (–6.146 eV)

Figure S46: **HOMO** to **HOMO–7** and respective energies of **1** obtained at the B3LYP(GD3BJ)/6-31G(d,p)/SMD(THF) level of theory. The contour value for the visualization of the isosurfaces is set to 0.03 and –0.03, respectively.

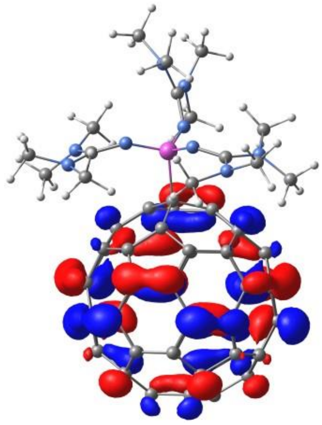

LUMO (–2.528 eV)

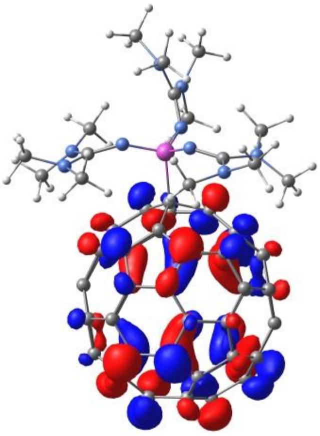

LUMO+1 (–2.497 eV)

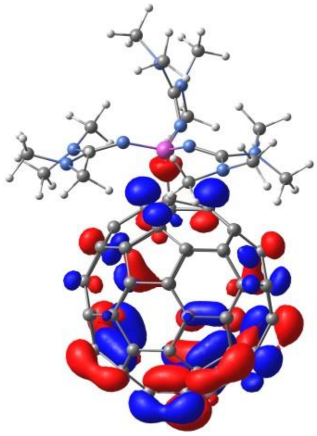

LUMO+2 (–2.130 eV)

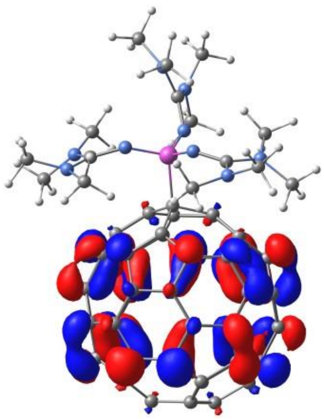

LUMO+3 (–1.448 eV)

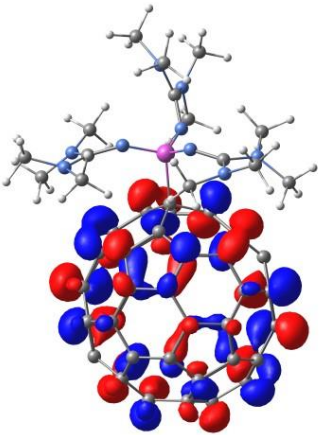

LUMO+4 (–1.275 eV)

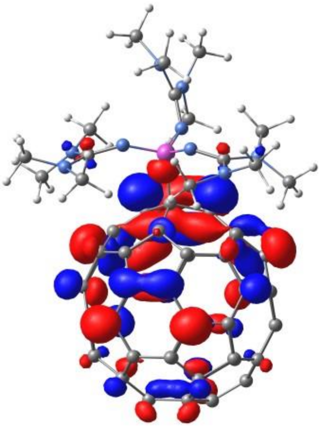

LUMO+5 (–0.999 eV)

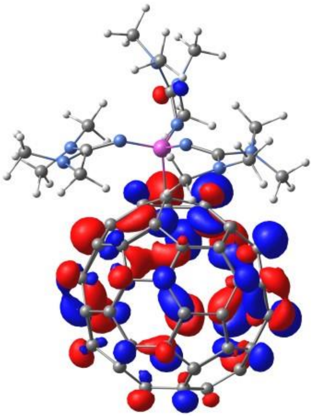

LUMO+6 (–0.344 eV)

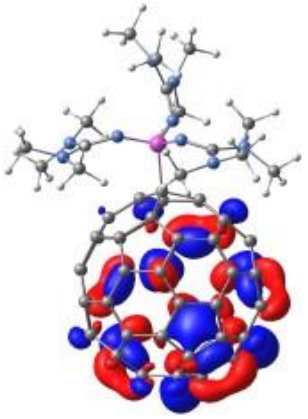

LUMO+7 (–0.329 eV)

Figure S47: **LUMO** to **LUMO+7** and respective energies of $P(tmg)_3$–C_{60} obtained at the B3LYP(GD3BJ)/6-31G(d,p)/SMD(THF) level of theory. The contour value for the visualization of the isosurfaces is set to 0.03 and –0.03, respectively.

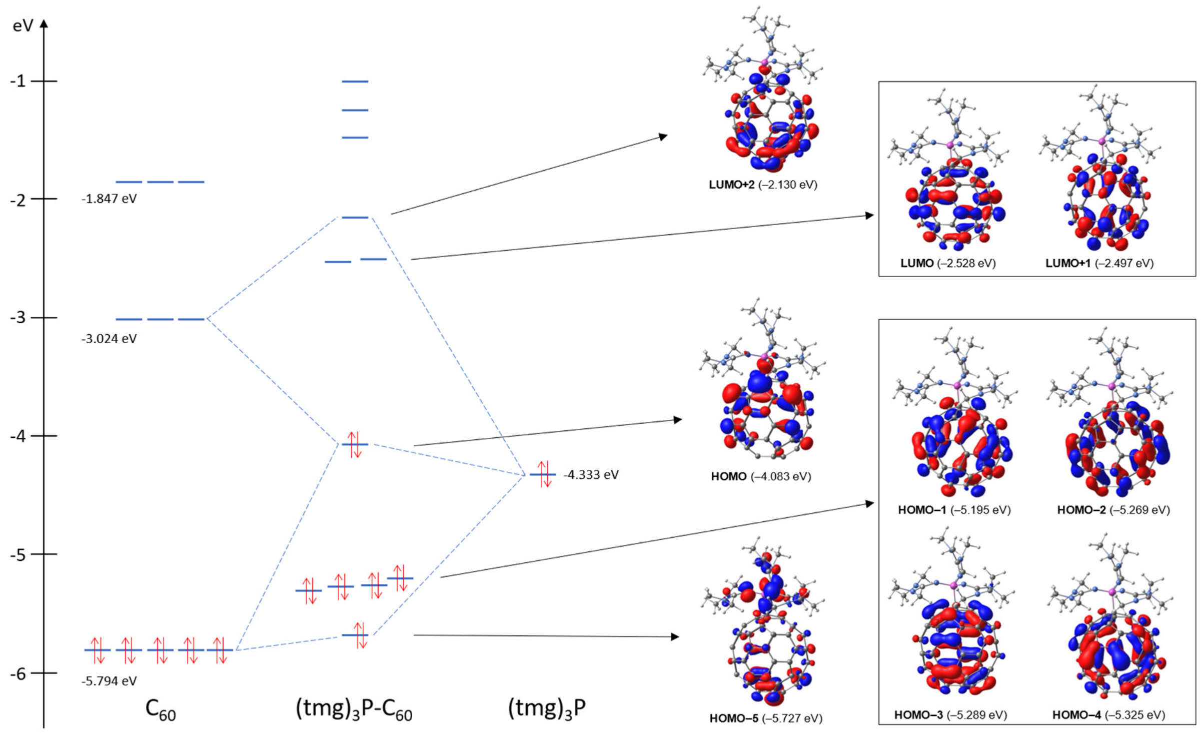

Figure S48: Frontier orbital interaction diagram of $(tmg)_3P–C_{60}$ (**1**) and the respective orbital energies obtained at the B3LYP(GD3BJ)/6-31G(d,p)/SMD(THF) level of theory.

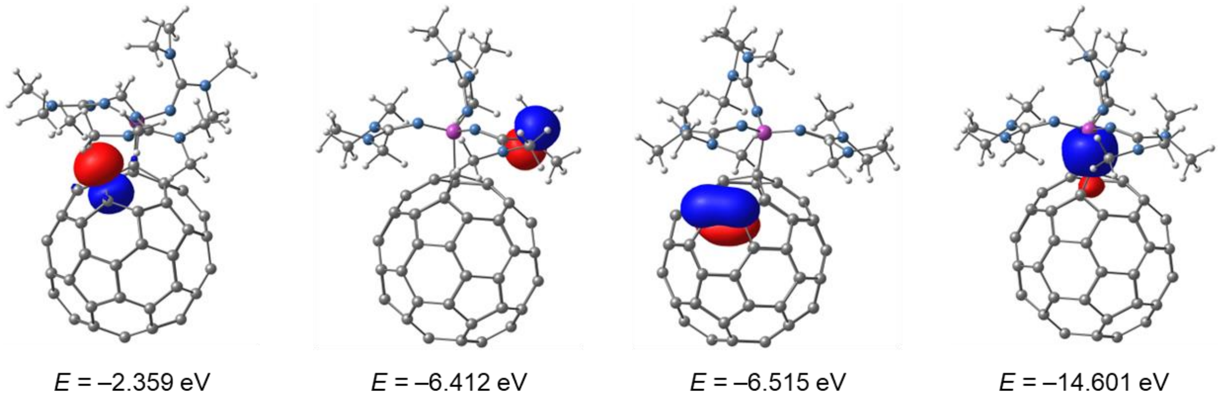

Figure S49: Natural bond orbital (NBO) of the lone pair at the carbon atom in vicinal position relative to the P–C_{60} bond (left), NBO of a lone pair at the guanidine N atoms (middle, left), NBO of a C_{60}–C_{60} carbon π bond (middle, right) and NBO of the P–C_{60} σ bond (left) of **1** obtained at the B3LYP(GD3BJ)/6-31G(d,p)/SMD(THF) level of theory. The contour value for the visualization of the isosurfaces is set to 0.05 and –0.05, respectively. (Note that for the NBO calculations, the keyword "resonance" was used to account for delocalized structures allowing occupancies below the default 1.90 threshold.)

The NBO analysis revealed that the NBO with the highest energy of E = –2.359 eV is of p-symmetry and located at one of the carbon atoms neighboring the quaternary C–PR_3 carbon atom (Figure S49). The occupancy is calculated with 1.17 electrons and 99.43 % p orbital character. The next NBOs lower in energy are depicted in Figure S49.

The nature of the P–C_{60} bond (Figure S49, right) was investigated. The σ bond (Wiberg Bond Index = 0.72, bond length: 1.886 Å) is calculated with an electron occupancy of 1.92 electrons with theoretical hybrid compositions of 0.589 P($sp^{2.79}$) + 0.808 C($sp^{3.89}$). This corresponds to a localization of 34.7 % on the P atom and a 65.3 % localization on the C atom. The P atom has 26.1 % s and 72.84 % p character while the C atom has 20.46% s and 79.52 % p character. The direction of the total dipole moment of μ_{total} = 25.27 D is aligned with the P–C_{60} bond.

3.3 Mechanistic Investigations

Transition state calculations of the reaction coordinate to form **1** resulted in the energy profile depicted in Figure S50. The activation barrier (ΔG) is 17.7 kJ mol^{-1} higher than the free energies of the separated C_{60} + $P(tmg)_3$ fragments and 80.9 kJ mol^{-1} higher than product **1**.

The formation of **1** *via* a charge separated state involving the formation of $C_{60}^{-\cdot}$ and $P(tmg)_3^{+\cdot}$ was calculated to be 104.9 kJ mol^{-1} higher than product **1** corresponding to a minimum energy barrier of 41.8 kJ/mol for the single electron transfer. This reaction pathway is therefore not regarded as a viable option.

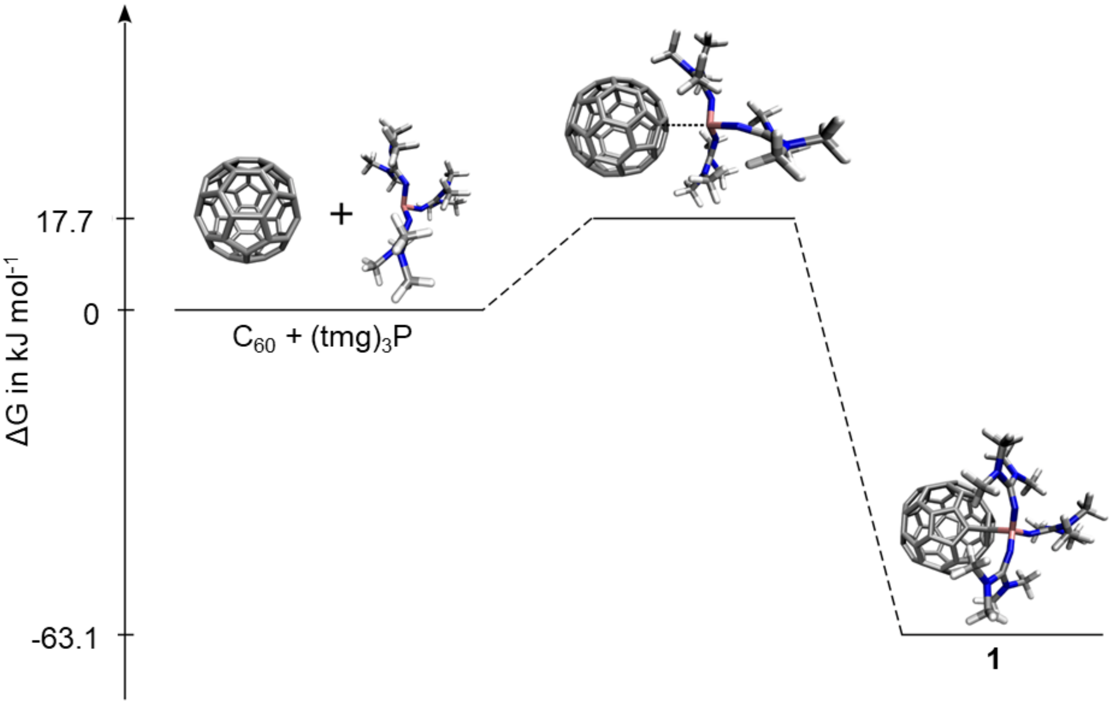

Figure S50: Reaction energy profile of the formation of **1** obtained at the B3LYP(GD3BJ)/6-31G(d,p)/SMD(THF) level of theory.

3.4 Free Energies and Chemical Shifts of 4

The NMR studies showed that addition of more than 1 equivalent of $P(tmg)_3$ to C_{60} leads to the formation of a mixture of regioisomers of $(tmg)_3PC_{60}P(tmg)_3$ (**4**) which could not be separated and further characterized. To gain insights into which potential isomers form during the reaction, we conducted DFT calculations of the different bisphoshine fullerene adducts and determined the difference in free energy (ΔG) following the reaction equation (1). If $\Delta G > 0$ kJ mol^{-1}, the reaction is considered endergonic and the isomer of **4** is disfavored, whereas it is favored for $\Delta G < 0$ kJ mol^{-1}.

$$\underset{\mathbf{1}}{(tmg)_3PC_{60}} + P(tmg)_3 \longrightarrow \underset{\mathbf{4}}{(tmg)_3PC_{60}P(tmg)_3} \qquad (1)$$

The Schlegel diagram for C_{60} and identification of positional isomers was taken from the literature.[32] Figure S51 depicts the diagram and a table of all different positional isomers of the doubly substituted C_{60} molecule. We reasoned that the substitution patterns adopted by the first five isomers are sterically significantly hindered and excluded them from further DFT studies.

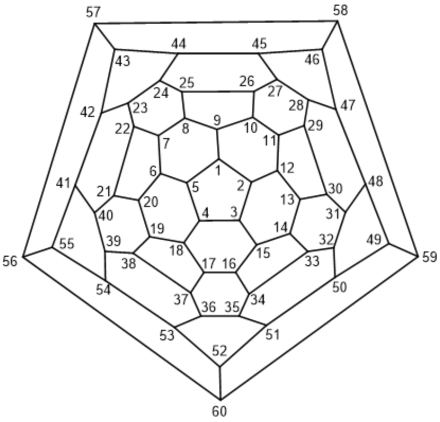

Isomer Label	Positions	$\Delta_R G^{298.15}$ (kJ mol-1)	Isomer Label	Positions	$\Delta_R G^{298.15}$ (kJ mol-1)
	C1 and C2	–	**4_I8**	C1 and C32	-17.56
	C1 and C3	–	**4_I9**	C1 and C33	-12.13
	C1 and C6	–	**4_I10**	C1 and C34	-20.06
	C1 and C7	–	**4_I11**	C1 and C35	-20.96
	C1 and C9	–	**4_I12**	C1 and C41	16.42
**4_I1**	C1 and C13	68.10	**4_I13**	C1 and C49	-22.16
**4_I2**	C1 and C14	16.71	**4_I14**	C1 and C50	-12.23
**4_I3**	C1 and C15	30.50	**4_I15**	C1 and C52	8.86
**4_I4**	C1 and C16	2.52	**4_I16**	C1 and C56	-25.03
**4_I5**	C1 and C23	-7.57	**4_I17**	C1 and C57	-17.59
**4_I6**	C1 and C24	13.40	**4_I18**	C1 and C60	-16.23
**4_I7**	C1 and C31	-23.57			

Figure S51: Schlegel diagram of C_{60} (left), table of the corresponding 23 different positional isomers for the doubly substituted C_{60} molecule and the free energy (ΔG) obtained at the B3LYP(GD3BJ)/6-31G(d,p)/(SMD)THF level of theory (right).

The ΔG values for the formation of isomers **4_I1** to **4_I4**, **4_I6**, **4_I12** and **4_I15** are positive, indicating reaction (1) to be endergonic and the formation of the bis-adduct to be disfavored. From a steric point of view, this result is not surprising for isomers **4_I1** to **4_I4** and **4_I6**, as the tetramethylguanidine groups of the two $P(tmg)_3$ moieties are in very close proximity to each other (Figure S52). The carbon atoms at which the phosphonium units are attached exhibit a distance of three or four bonds in these endergonic isomers. On the other hand, the comparably remote substitution positions in isomers **4_I12** and **4_I15** are energetically disfavored as well, which cannot be explained through sterics. Therefore, it is apparent that electronic effects play a role as well. Still, a general trend of lower ΔG values is observed if the two substituents are farther away from each other, more precise: ≥ 5 bonds between P*C*-*C*P, e.g. see isomers **4_I7** to **4_I11**, **4_I13**, **4_I14**, **4_I16, 4_I17** and **4_I18** These nine isomers are clearly exergonic

and thus energetically favored as products. The exergonic ΔG values obtained for isomer **4_I5** is less pronounced, which is likely due to the resulting electronic structure of the isomers. Overall, a total of eleven stable isomers were identified.

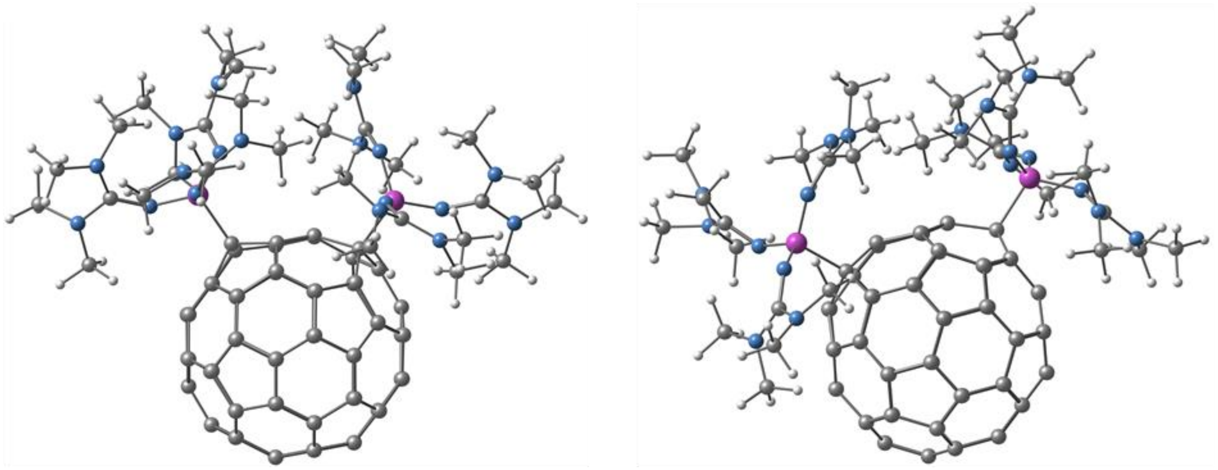

regioisomer of **4** (**4_I1**, C1 and C13)

regioisomer of **4** (**4_I6**, C1 and C24)

Figure S52: Selected optimized geometries of disfavored isomers (**4_I1** and **4_I6**) obtained by DFT calculations at the B3LYP(GD3BJ)/6-31G(d,p)/(SMD)THF level of theory.

The ΔG energy values give insight into which isomers can be expected to form according to reaction equation (1), and resonances of multiple isomers were observed in the ^{31}P NMR spectra (*vide supra*). In attempts to assign isomers to the experimental ^{31}P NMR chemical shifts obtained in the NMR study, these were calculated using the Gauge-Independent Atomic Orbital (GIAO) method.[21,27–31] For this purpose, a training set of selected phosphorus containing compounds with literature-known chemical shifts was collected. Calculations were performed employing the implicit solvent model density (SMD) approach[21] using the same solvent in which the chemical shifts of the compounds in training set are reported. The absolute magnetic shielding (σ_{calc}) which is obtained from the DFT calculations is converted to a value for the chemical shift ($\delta(^{31}P)_{calc}$) using a reference system according to equation (2).

$$\delta(^{31}P)_{calc} = \sigma(reference)_{calc} - \sigma(^{31}P)_{calc} + \delta(reference)_{exp} \quad (2)$$

In experimental ^{31}P NMR spectroscopy, an 85% H_3PO_4 solution in H_2O is used as external reference which is a roughly 1:1 molar solution.[33] Therefore, H_3PO_4 is used as well as reference system to convert the absolute magnetic shielding term to chemical shifts. Yet, calculations that well represent experimental conditions are difficult because the interaction with water, ionization and aggregation of the acid would need to be considered. A sufficiently accurate method was previously reported, where the absolute magnetic shielding term ($\sigma(reference)_{calc}$) for H_3PO_4 was calculated by simply using H_2O as implicit solvent. Additionally, subsequent scaling of the calculated ^{31}P NMR shifts helps to improve their accuracy.[24] Starting from the optimized geometries obtained at the B3LYP(GD3BJ)/6-31G(d,p)/(SMD)*solvent* level of theory, three different functional / basis set combinations (**A**, **B** and **C**) were used to calculate the absolute magnetic shielding terms (σ_{calc}), $\delta(^{31}\mathrm{P})_{\mathrm{exp}}$ of the training set and their calculated ^{31}P NMR shifts ($\delta(^{31}\mathrm{P})_{\mathrm{calc}}$) according to equation (2). All following $\delta(^{31}\mathrm{P})$ values are given in ppm.

Table S3 collects the experimental ^{31}P NMR shifts ($\delta(^{31}P)_{exp}$) of the training set and their calculated absolute magnetic shielding terms (σ_{calc}).

Table S4 contains the experimental ^{31}P NMR shifts ($\delta(^{31}P)_{exp}$) of the training set and their calculated ^{31}P NMR shifts ($\delta(^{31}P)_{calc}$) according to equation (2). All following $\delta(^{31}P)$ values are given in ppm.

Table S3: Training set of phosphorus compounds, their experimental ^{31}P NMR shift ($\delta(^{31}P)_{exp}$) and absolute magnetic shielding term (σ_{calc})calculated at different levels of theory.

Compound	Solvent	$\delta(^{31}P)_{exp}$	A σ_{calc}	B σ_{calc}	C σ_{calc}
H_3PO_4	H_2O	0.0	368.649	366.201	284.830
$POMe_3$[24]	$CHCl_3$	141.4	220.294	225.062	132.886
PMe_3[34]	THF	-61.5	432.354	455.873	367.291
PMe_2H[24,35]	THF#	-98.5	465.968	491.917	412.040
$PMeH_2$[24,35]	THF#	-163.5	522.103	547.805	485.652
PH_3[24,36]	CCl_4	-238.0	593.698	616.424	577.669
PMe_2Ph[37]	THF	-45.3	411.390	429.669	343.648
$PMePh_2$[37]	THF	-26.2	386.586	401.754	319.083
PPh_3[38]	THF	-5.6	366.506	380.973	304.630
PEt_3[34]	THF	-18.8	390.722	409.484	318.903
$PnBu_3$[34]	THF	-31.7	407.303	427.516	341.899
$PiPr_3$[34]	THF	20.7	352.446	365.600	271.635
$PtBu_3$[34]	THF	63.4	306.738	316.988	222.626
$P(tmg)_3$	THF	83.5	257.509	267.304	186.465
$P(tmg)_3$[2]	C_6H_6	85.3	257.008	271.169	188.830
**1**	THF	-18.7	382.940	377.188	312.610
P*i*Pr$_2$NI*i*Pr[39]	C_6H_6	63.2	307.269	317.076	227.466
$P(tmg)_3–CO_2$[2]	ACN	-14.2	373.819	369.990	303.108
$[(NIDipp)_2(CH_3)(C_2)P]$[40]	C_6H_6‡	-33.0	412.580	409.307	337.037

A, B, C: geometry optimization at B3LYP(GD3BJ)/6-31G(d,p)/(SMD)*solvent* level of theory.

A: magnetic shielding calculated at B3LYP(GD3BJ)/6-31G(d,p)/(SMD)*solvent* level of theory.

B: magnetic shielding calculated at M06-2X/6-31G(d,p)/(SMD)*solvent* level of theory.

C: magnetic shielding calculated at M06-2X/6-311+G(2d,p)/(SMD)*solvent* level of theory.

Dipp = 2,6-diisopropylphenyl; #solvent was not specified in publication; ‡optimization in vacuum, magnetic shielding calculation with implicit solvent.

Table S4: Training set of phosphorus compounds, their experimental, **unscaled** ^{31}P NMR shift ($\delta(^{31}P)_{exp}$) and their calculated ^{31}P NMR shifts ($\delta(^{31}P)_{calc}$) using equation (2).

Compound	Solvent	$\delta(^{31}P)_{exp}$	**A** $\delta(^{31}P)_{calc}$	**B** $\delta(^{31}P)_{calc}$	**C** $\delta(^{31}P)_{calc}$
H_3PO_4	H_2O	0	0.00	0.00	0.00
$POMe_3$[24]	$CHCl_3$	141.4	148.36	141.14	151.94
PMe_3[34]	THF	-61.5	-63.71	-89.67	-82.46
PMe_2H[24,35]	THF#	-98.5	-97.32	-125.72	-127.21
$PMeH_2$[24,35]	THF#	-163.5	-153.45	-181.60	-200.82
PH_3[24,36]	CCl_4	-238	-225.05	-250.22	-292.84
PMe_2Ph[37]	THF	-45.3	-42.74	-63.47	-58.82
$PMePh_2$[37]	THF	-26.2	-17.94	-35.55	-34.25
PPh_3[38]	THF	-5.6	2.14	-14.77	-19.80
PEt_3[34]	THF	-18.8	-22.07	-43.28	-34.07
$PnBu_3$[34]	THF	-31.7	-38.65	-61.31	-57.07
$PiPr_3$[34]	THF	20.7	16.20	0.60	13.20
$PtBu_3$[34]	THF	63.4	61.91	49.21	62.20
$P(tmg)_3$	THF	83.5	111.14	98.90	98.37
$P(tmg)_3$[2]	C_6H_6	85.3	111.64	95.03	96.00
**1**	THF	-18.7	-14.29	-10.99	-27.78
P*i*Pr_2NI*i*Pr[39]	C_6H_6	63.22	61.38	49.13	57.36
$P(tmg)_3$–CO_2[2]	ACN	-14.2	-5.17	-3.79	-18.28
$[(NIDipp)_2(CH_3)(C_2)P]$[40]	C_6H_6‡	-33.0	-43.93	-43.11	-52.21

A, B, C: geometry optimization at B3LYP(GD3BJ)/6-31G(d,p)/(SMD)*solvent* level of theory.

A: magnetic shielding calculated at B3LYP(GD3BJ)/6-31G(d,p)/(SMD)*solvent* level of theory.

B: magnetic shielding calculated at M06-2X/6-31G(d,p)/(SMD)*solvent* level of theory.

C: magnetic shielding calculated at M06-2X/6-311+G(2d,p)/(SMD)*solvent* level of theory.

Dipp = 2,6-diisopropylphenyl; #solvent was not specified in publication; ‡optimization in vacuum, magnetic shielding calculation with implicit solvent.

To give an empirical scaling relationship,[41] the calculated chemical shifts are plotted against the experimental chemical shifts obtained from equation (2). The three plots belonging to the calculation method **A**, **B** and **C** are depicted in Figure S53. The linear regression nicely fits the plots even for the smallest functional/basis set combination **A**. The slope and intercept obtained from the linear fit were then used to convert the absolute magnetic shielding terms (σ_{calc}) of the training set to scaled ^{31}P NMR shifts $\delta(\,^{31}P\,)_{calc,scaled}$. These values are collected in Table S5

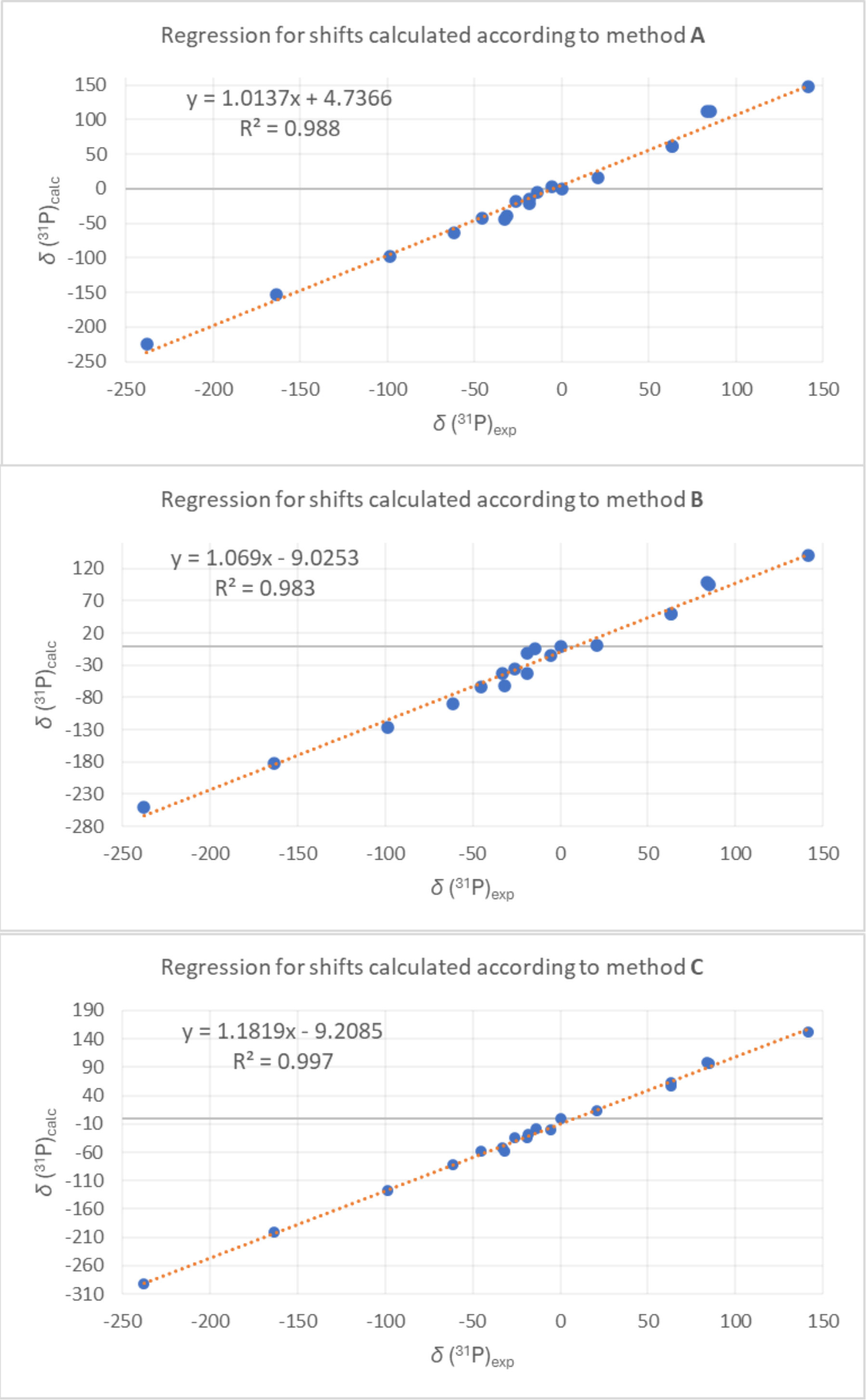

Figure S53: Plots of calculated, unscaled ^{31}P NMR shifts obtained using different functional/basis set combinations **A**, **B** and **C** against experimentally obtained ^{31}P NMR shifts and their respective linear regression.

Table S5: Training set of phosphorus compounds, their experimental ^{31}P NMR shift ($\delta(^{31}P)_{exp}$) and their calculated and **scaled** ^{31}P NMR shifts ($\delta(^{31}P)_{calc}$) using equation (2).

**Compound**	**Solvent**	$\delta(^{31}P)_{exp}$	**A**	**B**	**C**		**A**	**B**	**C**
			$\delta(^{31}P)_{calc,scaled}$				Deviation (absolute)		
H_3PO_4	H_2O	0	-4.7	-8.4	-7.8		4.7	3.8	0.7
$POMe_3$[24]	$CHCl_3$	141.4	141.7	123.6	120.8		0.3	18.1	2.8
PMe_3[34]	THF	-61.5	-67.5	-92.3	-77.6		6.0	24.8	14.8
PMe_2H[24,35]	THF#	-98.5	-100.7	-126.0	-115.4		2.2	25.4	10.6
$PMeH_2$[24,35]	THF#	-163.5	-156.1	-178.3	-177.7		7.4	22.3	0.6
PH_3[24,36]	CCl_4	-238.0	-226.7	-242.5	-255.6		11.3	15.8	13.0
PMe_2Ph[37]	THF	-45.3	-46.8	-67.8	-57.6		1.5	21.0	10.3
$PMePh_2$[37]	THF	-26.2	-22.4	-41.7	-36.8		3.8	19.3	4.9
PPh_3[38]	THF	-5.6	-2.6	-22.3	-24.5		3.0	19.7	2.3
PEt_3[34]	THF	-18.8	-26.4	-48.9	-36.6		7.6	22.5	12.3
$PnBu_3$[34]	THF	-31.7	-42.8	-65.8	-56.1		11.1	23.0	9.7
$PiPr_3$[34]	THF	20.7	11.3	-7.9	3.4		9.4	19.2	11.3
$PtBu_3$[34]	THF	63.4	56.4	37.6	44.8		7.0	18.8	7.2
$Ptmg_3$	THF	83.5	105.0	84.1	75.4		21.5	20.9	8.6
$Ptmg_3$[2]	C_6H_6	85.3	105.5	80.5	73.4		20.2	25.0	7.0
**1**	THF	-18.7	-18.8	-18.7	-31.3		0.1	0.1	12.6
P*i*Pr_2NI*i*Pr[39]	C_6H_6	63.22	55.9	37.5	40.7		7.3	18.4	3.2
$P(tmg)_3$–CO_2[2]	ACN	-14.2	-9.8	-12.0	-23.3		4.4	2.2	11.3
$[(NIDipp)_2(CH_3)(C_2)P]$[40]	C_6H_6‡	-33.0	-48.0	-48.8	-52.0		15.0	0.8	3.2
**Regression:**		**slope (m)**	1.014	1.069	1.182	**MAD**	4.5	6.5	3.9
		**intercept (b)**	4.737	9.025	9.209	**RMSD**	9.6	18.8	8.9

Even though the functional/basis set combination used for method **C** shows the best R^2 value in the linear regression and also the smallest mean absolute deviation (MAD) and smallest root mean square deviation (RMSD), we chose to use method **A** to assess the theoretical ^{31}P NMR shift ($\delta(^{31}P)_{calc,scaled}$) of the 18 isomers of **4** considered in this study. The linear regression for **A** nicely fits the plot with a good R^2 value and the MAD and RMSD are similar to that of **C**. Thus, Table S6 collects the absolute magnetic shielding term (σ_{calc}), the unscaled ^{31}P NMR shifts ($\delta(^{31}P)_{calc}$) and scaled ^{31}P NMR shifts ($\delta(^{31}P)_{calc,scaled}$) of the isomers 6–18.

Table S6: Absolute magnetic shielding term (σ_{calc}), **unscaled** ^{31}P NMR shifts ($\delta(^{31}P)_{calc}$) and **scaled** ^{31}P NMR shifts ($\delta(^{31}P)_{calc,scaled}$) of the bis-(P(tmg$_3$)–C$_{60}$ isomers 6–18 using DFT method **A**. Equation (2) was used to calculate the unscaled shifts and the slope (m = 1.014) and intercept (b = 4.737) from the linear regression were used for scaling.

Isomere No.	Solvent	$\sigma_{calc,1}$	$\sigma_{calc,2}$	$\delta(^{31}P)_{calc,1}$	$\delta(^{31}P)_{calc,2}$	$\delta(^{31}P)_{calc,scaled,1})$	$\delta(^{31}P)_{calc,scaled,2})$
**4_I1** (C1-C13)	THF	363.961	362.279	4.69	6.37	**0.0**	**1.6**
**4_I2** (C1-C14)	THF	380.176	372.732	-11.53	-4.08	**-16.0**	**-8.7**
**4_I3** (C1- C15	THF	375.874	372.261	-7.23	-3.61	**-11.8**	**-8.2**
**4_I4** (C1-C16)	THF	382.340	377.354	-13.69	-8.71	**-18.2**	**-13.3**
**4_I5** (C1-C23)	THF	379.729	379.694	-11.08	-11.05	**-15.6**	**-15.6**
**4_I6** (C1-C24)	THF	373.916	373.723	-5.27	-5.07	**-9.9**	**-9.7**
**4_I7** (C1-C31)	THF	374.101	379.439	-5.45	-10.79	**-10.1**	**-15.3**
**4_I8** (C1-C32)	THF	378.153	379.505	-9.50	-10.86	**-14.0**	**-15.4**
**4_I9** (C1-C33)	THF	383.852	381.447	-15.20	-12.80	**-19.7**	**-17.3**
**4_I10** (C1-C34)	THF	375.468	378.446	-6.82	-9.80	**-11.4**	**-14.3**
**4_I11** (C1-C35)	THF	380.870	378.035	-12.22	-9.39	**-16.7**	**-13.9**
**4_I12** (C1-C41)	THF	377.839	375.428	-9.19	-6.78	**-13.7**	**-11.4**
**4_I13** (C1-C49)	THF	377.840	381.366	-9.19	-12.72	**-13.7**	**-17.2**
**4_I14** (C1-C50)	THF	380.755	378.797	-12.11	-10.15	**-16.6**	**-14.7**
**4_I15** (C1-C52)	THF	379.222	379.785	-10.57	-11.14	**-15.1**	**-15.7**
**4_I16** (C1-C56)	THF	382.195	381.794	-13.55	-13.15	**-18.0**	**-17.6**
**4_I17** (C1-C57)	THF	380.509	377.186	-11.86	-8.54	**-16.4**	**-13.1**
**4_I18** (C1-C60)	THF	387.601	383.653	-18.95	-15.00	**-23.4**	**-19.5**

The obtained values range from +1.6 ppm to –23.4 ppm. Apart from the values obtained for **4_I1**, all computationally determined ^{31}P NMR shifts lie in a reasonable range corresponding to the observed ^{31}P NMR shift range observed in the NMR study (Figure S34, *vide supra*). Considering the deviation of the computationally determined ^{31}P NMR shifts, assigning the observed shifts to a specific isomer is not possible in this case. Yet, regarding the energetically favored isomers ($\Delta G < 0$ kJ mol^{-1}), some calculated ^{31}P NMR shifts are very similar, e.g. of isomers **4_I5** and **4_I8**, while other isomers show two distinctly different shifts, e.g. isomer **4_I7** to **4_I11**, **4_I17** and **4_I18**. Therefore, it is reasonable to assume that the resonance of the two phosphorus atoms of one isomer can appear simply as one singlet or two separate signals in the experimental ^{31}P NMR spectrum. In case of the latter and due to the conjugated nature of the C$_{60}$ fragment, the – in some cases – observed long-range P–P coupling in the experimental ^{31}P NMR spectrum is reasonable.

3.5 Cartesian Coordinates of the Optimized Geometries

Table S7: Cartesian coordinates of the optimized geometry of **1** at the B3LYP(GD3BJ)/6-31G(d,p)/SMD(THF) level of theory.

Atom	X	Y	Z	Atom	X	Y	Z
P	3.40772	0.24638	-0.21362	H	2.55587	2.01943	-3.01151
N	3.94547	-0.82895	-1.30018	H	2.58904	3.56716	-3.88598
N	3.78974	1.68969	-0.85780	H	4.10158	2.66733	-3.60431
N	3.88878	0.03916	1.33964	H	4.43089	5.11739	-2.86821
C	1.52657	0.14297	-0.12699	H	2.84307	5.70254	-2.32651
C	3.66943	-2.02788	-1.76068	H	4.14403	5.41047	-1.14901
C	3.43288	2.95051	-0.76152	H	2.88491	-1.55462	3.04188
C	4.96354	-0.44211	1.91190	H	3.08246	-0.15748	4.11304
C	0.88928	0.41746	-1.48338	H	3.63877	-1.76576	4.63776
C	1.05157	-1.21707	0.42858	H	6.24340	-1.54092	4.68556
C	0.95635	1.09806	0.94444	H	5.52158	0.05951	4.96369
N	3.50848	-3.14746	-0.99411	H	6.76799	-0.13940	3.72551
N	3.57560	-2.19466	-3.11637	H	6.00175	1.12181	0.10872
N	3.24801	3.64344	0.40261	H	6.55813	-0.32467	-0.74385
N	3.26555	3.65882	-1.92237	H	7.66059	0.54524	0.35315
N	4.86578	-0.88767	3.20894	H	6.62147	-2.38155	2.32234
N	6.20161	-0.57471	1.33195	H	8.02316	-1.29605	2.16363
C	0.26898	-0.61736	-2.23956	H	7.41073	-2.17466	0.74790
C	0.17392	1.62313	-1.73986	C	-1.94412	-0.74443	-3.36520
C	0.47042	-2.18992	-0.36474	C	-1.11756	-2.63711	-2.02711
C	0.60697	-1.06649	1.78743	C	-2.06068	2.04047	-2.74535
C	0.28644	2.26585	0.62859	C	-1.36358	3.24555	-0.71652
C	0.55020	0.35170	2.10431	C	-1.49581	-3.37609	-0.82272
C	3.97124	-3.16975	0.38212	C	-0.89059	-2.99191	1.54201
C	2.67314	-4.27848	-1.38569	C	-1.35580	-1.44252	3.24750
C	3.40778	-1.01407	-3.95329	C	-1.10131	2.07335	2.67036
C	4.10334	-3.36752	-3.81017	C	-1.77704	3.37255	0.68054
C	3.80829	3.17088	1.65745	C	-1.41204	-0.08463	3.54952
C	2.31461	4.75785	0.53178	C	-3.28328	1.30490	-3.03192
C	3.12557	2.93117	-3.17610	C	-3.22629	-0.05626	-3.33490
C	3.68458	5.05071	-2.06805	C	-4.31869	1.81078	-2.14542
C	3.54192	-1.09654	3.77823	C	-2.34279	3.01025	-1.70258
C	5.91140	-0.61950	4.19533	C	-4.20210	-0.97044	-2.76436
C	6.62239	0.23271	0.19697	C	-3.72919	2.85841	-1.31940
C	7.11502	-1.66205	1.67059	C	-5.25712	0.93475	-1.60207
C	-0.77762	-0.03330	-3.08762	C	-2.13001	-2.08439	-2.83732
C	0.11129	-1.93634	-1.76862	C	-5.19763	-0.48465	-1.91810
C	-0.83657	1.37325	-2.77427	C	-3.51677	-2.21897	-2.44993
C	-0.07970	2.59955	-0.75738	C	-4.12665	2.99461	0.01752
C	-0.50751	-3.10306	0.19831	C	-5.65947	1.06762	-0.20900
C	-0.31449	-1.95349	2.36143	C	-5.56369	-1.22672	-0.72001
C	-0.75179	2.77298	1.50729	C	-3.87931	-2.94023	-1.30423
C	-0.42990	0.83459	2.98193	C	-3.12256	3.25082	1.03467
H	4.81600	-2.49595	0.49019	C	-5.09803	2.07248	0.58045
H	4.29494	-4.18540	0.62753	C	-5.84421	-0.26722	0.33447
H	3.18219	-2.87314	1.08216	C	-4.91055	-2.42333	-0.42099
H	2.08747	-4.02719	-2.26757	C	-2.84032	-3.52248	-0.47397
H	1.97862	-4.49797	-0.56941	C	-3.48936	2.50969	2.23562
H	3.26915	-5.17647	-1.58486	C	-4.71378	1.78343	1.95657
H	2.75282	-0.30023	-3.45815	C	-5.48271	-0.54645	1.65649
H	2.95123	-1.32412	-4.89736	C	-4.52617	-2.71481	0.95463
H	4.36752	-0.52552	-4.16616	C	-3.24336	-3.39120	0.92129
H	4.87390	-3.04543	-4.52015	C	-2.49943	1.93785	3.03859
H	3.32090	-3.89542	-4.36566	C	-4.89925	0.50012	2.47837
H	4.56395	-4.05570	-3.10352	C	-4.80366	-1.79288	1.96780
H	4.67908	2.54962	1.46387	C	-2.28830	-3.13716	1.90855
H	4.11453	4.03926	2.24967	C	-2.69318	0.60060	3.58277
H	3.08393	2.58565	2.23348	C	-3.86889	-0.10156	3.30953
H	1.69942	4.84050	-0.36169	C	-3.80994	-1.52047	2.99382
H	1.65310	4.56169	1.38130	C	-2.57775	-2.17680	2.96485
H	2.83364	5.70695	0.70900				

Table S8: Cartesian coordinates of the transition state (TS) calculations of C_{60} + P(tmg)$_3$ at the B3LYP(GD3BJ)/6-31G(d,p)/SMD(THF) level of theory.

Atom	X	Y	Z	Atom	X	Y	Z
P	4.05234	0.29128	0.42643	H	2.51308	1.80635	-2.74372
N	4.12215	-0.98397	-0.72330	H	2.42696	3.34775	-3.62895
N	3.98151	1.59252	-0.69737	H	3.99718	2.54882	-3.36253
N	5.63478	0.44436	1.08220	H	4.54881	4.82452	-2.49988
C	0.84651	0.26410	0.43306	H	2.90153	5.48373	-2.37649
C	3.75317	-2.21855	-0.59661	H	3.90831	5.31449	-0.92634
C	3.49447	2.78178	-0.53270	H	6.35664	-0.65030	3.22651
C	6.77940	-0.05644	0.72057	H	7.28366	0.84140	3.45718
C	0.69261	0.32899	-0.95310	H	8.07133	-0.73500	3.70487
C	0.58074	-0.98600	1.13153	H	9.80871	-0.72384	1.81646
C	0.35132	1.35139	1.26575	H	9.39884	0.99322	2.02357
N	3.57969	-2.94643	0.56821	H	9.32120	0.25499	0.41662
N	3.45516	-2.94676	-1.76034	H	6.08308	1.07876	-1.48386
N	3.25887	3.44854	0.65640	H	6.08462	-0.44612	-2.35494
N	3.11321	3.50412	-1.67303	H	7.56524	0.55354	-2.33862
N	7.74288	-0.27531	1.70242	H	8.18890	-2.14041	0.15196
N	7.17267	-0.44451	-0.54547	H	8.95969	-1.35961	-1.24750
C	0.28384	-0.85149	-1.69714	H	7.47038	-2.30028	-1.46430
C	0.05299	1.48298	-1.56264	C	-1.69272	-1.21744	-3.13073
C	0.18732	-2.11465	0.41666	C	-1.09556	-2.87475	-1.40310
C	-0.06225	-0.67073	2.39737	C	-1.97276	1.61592	-2.96762
C	-0.26297	2.45652	0.67898	C	-1.69143	3.15129	-1.05691
C	-0.20497	0.77513	2.48002	C	-1.65167	-3.45042	-0.18681
C	4.18734	-2.57023	1.83182	C	-1.47054	-2.68219	2.15391
C	2.70127	-4.10258	0.66826	C	-2.25572	-0.92017	3.50227
C	3.28246	-2.17940	-2.98377	C	-1.98105	2.48742	2.44958
C	4.14144	-4.21682	-1.99606	C	-2.33516	3.46623	0.21021
C	3.96397	3.13831	1.88899	C	-2.39240	0.46554	3.58152
C	2.27001	4.50605	0.79873	C	-3.11244	0.79150	-3.34041
C	3.01314	2.75697	-2.91731	C	-2.97552	-0.59442	-3.42007
C	3.64311	4.85237	-1.87525	C	-4.30179	1.37148	-2.73616
C	7.33814	-0.19600	3.09564	C	-2.45773	2.70583	-2.13573
C	9.14117	0.07579	1.47525	C	-4.02213	-1.45979	-2.89880
C	6.70066	0.22190	-1.74543	C	-3.89676	2.55379	-1.99094
C	7.99730	-1.61713	-0.78440	C	-5.30565	0.54168	-2.23695
C	-0.60064	-0.42682	-2.77159	C	-1.94640	-2.46775	-2.43278
C	0.04464	-2.05354	-1.03137	C	-5.16277	-0.90367	-2.31980
C	-0.74342	1.01840	-2.68863	C	-3.38579	-2.61682	-2.28755
C	-0.40826	2.53225	-0.76803	C	-4.51384	2.85741	-0.77683
C	-0.85570	-2.98194	0.93716	C	-5.94807	0.85788	-0.97065
C	-1.06552	-1.50118	2.89907	C	-5.71698	-1.48050	-1.10473
C	-1.44992	3.03876	1.28266	C	-3.91819	-3.17063	-1.12270
C	-1.34510	1.33181	3.06134	C	-3.71588	3.32284	0.34692
H	4.95900	-1.82036	1.68094	C	-5.55945	1.99096	-0.25599
H	4.64814	-3.45518	2.29026	C	-6.20210	-0.39185	-0.27095
H	3.44618	-2.16894	2.53415	C	-5.10702	-2.58969	-0.51869
H	2.09797	-4.19875	-0.23166	C	-3.03219	-3.59565	-0.04998
H	2.03105	-3.97296	1.52529	C	-4.27002	2.74593	1.56249
H	3.26616	-5.03204	0.82275	C	-5.40960	1.92295	1.18977
H	2.69750	-1.28423	-2.78303	C	-6.05856	-0.45724	1.11518
H	2.75121	-2.80266	-3.71082	C	-4.95680	-2.65777	0.92705
H	4.24137	-1.86944	-3.42498	C	-3.67460	-3.27950	1.21693
H	5.05629	-4.06118	-2.58856	C	-3.42071	2.33709	2.59113
H	3.49149	-4.90494	-2.54703	C	-5.65357	0.72448	1.86058
H	4.42865	-4.68686	-1.05745	C	-5.42289	-1.61388	1.72665
H	4.80642	2.47310	1.70154	C	-2.91006	-2.83242	2.29524
H	4.34342	4.06966	2.33115	C	-3.67519	1.08724	3.29079
H	3.30076	2.66150	2.62151	C	-4.76831	0.29807	2.93308
H	1.64097	4.55770	-0.08707	C	-4.62583	-1.14722	2.85053
H	1.63486	4.28990	1.66510	C	-3.39577	-1.74326	3.12873
H	2.73917	5.48571	0.96432				

Table S9: Cartesian coordinates of the optimized geometry of C_{60} at the B3LYP(GD3BJ)/6-31G(d,p)/SMD(THF) level of theory.

Atom	X	Y	Z
C	-2.41985	-2.58793	0.24826
C	-2.88831	-1.85241	-0.91583
C	-2.15486	-1.88364	-2.10230
C	-0.92189	-2.65151	-2.17528
C	-0.47236	-3.35694	-1.05864
C	-1.23687	-3.32439	0.17830
C	-2.71226	-1.79208	1.43007
C	-3.36149	-0.56492	0.99632
C	-3.47056	-0.60220	-0.45354
C	-3.29580	0.56578	-1.19647
C	-1.97235	-0.66604	-2.87655
C	0.02253	-1.90843	-2.99466
C	1.37802	-1.90120	-2.66407
C	1.84675	-2.63650	-1.50000
C	0.94076	-3.34901	-0.71391
C	1.04973	-3.31194	0.73591
C	-0.29617	-3.29636	1.28722
C	-0.57677	-2.53333	2.42102
C	-1.80975	-1.76520	2.49360
C	-3.08236	0.63878	1.64429
C	-2.14173	0.66659	2.75334
C	-1.51867	-0.51050	3.16909
C	-0.10563	-0.50286	3.51360
C	0.47651	-1.75310	3.05151
C	1.76750	-1.76789	2.52253
C	2.06001	-2.56339	1.34058
C	3.00482	-1.82066	0.52113
C	2.90001	-1.85633	-0.86972
C	-0.62687	-0.68149	-3.42841
C	2.71226	1.79208	-1.43007
C	1.80975	1.76520	-2.49360
C	0.57677	2.53333	-2.42102
C	0.29617	3.29636	-1.28722
C	1.23687	3.32439	-0.17830
C	2.88831	1.85241	0.91583
C	3.47056	0.60220	0.45354
C	3.36149	0.56491	-0.99632
C	3.08236	-0.63878	-1.64429
C	2.14173	-0.66659	-2.75334
C	1.51867	0.51050	-3.16909
C	-0.47651	1.75310	-3.05151
C	-1.76749	1.76790	-2.52253
C	-2.06001	2.56339	-1.34058
C	-1.04973	3.31195	-0.73591
C	-0.94076	3.34901	0.71391
C	0.47236	3.35694	1.05864
C	0.92190	2.65152	2.17528
C	2.15486	1.88364	2.10230
C	3.29580	-0.56578	1.19647
C	2.53081	-0.53310	2.43311
C	1.97235	0.66604	2.87655
C	0.62687	0.68149	3.42840
C	-0.02253	1.90843	2.99465
C	-1.37802	1.90120	2.66406
C	-1.84675	2.63649	1.49999
C	-2.90001	1.85633	0.86972
C	-3.00482	1.82067	-0.52113
C	0.10563	0.50286	-3.51360
C	-2.53081	0.53310	-2.43310
C	2.41984	2.58792	-0.24826

Table S10: Cartesian coordinates of the optimized geometry of $P(tmg)_3$ at the B3LYP(GD3BJ)/6-31G(d,p)/SMD(THF) level of theory.

Atom	X	Y	Z
P	0.30914	-0.11497	-0.68644
N	-0.82572	-1.02130	0.25564
N	1.65551	-0.28110	0.38973
N	-0.11342	1.54078	-0.49994
C	-1.75783	-1.76078	-0.26169
C	2.89421	-0.34452	0.01086
C	-0.94577	2.12711	0.30777
N	-2.35329	-1.61862	-1.50836
N	-2.24400	-2.84393	0.46775
N	3.44150	0.13338	-1.17049
N	3.81925	-0.97103	0.84725
N	-1.56961	3.29711	-0.11852
N	-1.33584	1.71695	1.56953
C	-2.49914	-0.33131	-2.16304
C	-2.75987	-2.75610	-2.31860
C	-1.49859	-3.26068	1.64327
C	-3.67981	-3.08324	0.57739
C	2.90776	1.28595	-1.87536
C	4.54918	-0.52372	-1.84571
C	3.29608	-1.75791	1.95211
C	5.08895	-0.31794	1.15600
C	-1.46620	3.66560	-1.51965
C	-1.74662	4.43245	0.78013
C	-0.44471	0.99794	2.46055
C	-2.71352	1.80118	2.02335
H	-2.30344	0.47464	-1.45953
H	-3.52866	-0.22768	-2.53282
H	-1.81703	-0.21904	-3.01491
H	-2.47561	-3.68648	-1.82862
H	-2.25866	-2.70927	-3.29459
H	-3.84379	-2.76764	-2.49798
H	-0.42913	-3.18504	1.45149
H	-1.75604	-4.30011	1.87284
H	-1.73063	-2.64451	2.52494
H	-4.05853	-2.71628	1.54303
H	-3.90256	-4.15397	0.50913
H	-4.21700	-2.56320	-0.21373
H	2.13893	1.78016	-1.28204
H	3.71992	2.00103	-2.07042
H	2.46615	1.00353	-2.83926
H	4.79350	-1.45927	-1.34420
H	4.26364	-0.74688	-2.88220
H	5.44853	0.10716	-1.87502
H	2.44716	-2.35353	1.61768
H	4.08709	-2.42442	2.31116
H	2.96080	-1.13103	2.79162
H	5.03464	0.19401	2.12837
H	5.90110	-1.05189	1.20167
H	5.33247	0.42579	0.39940
H	-1.53968	2.77676	-2.14567
H	-0.51446	4.16619	-1.75132
H	-2.28424	4.35133	-1.76445
H	-2.76157	4.83994	0.70521
H	-1.03785	5.23452	0.52865
H	-1.56469	4.13196	1.81068
H	0.55940	0.96154	2.04122
H	-0.77068	-0.03731	2.59981
H	-0.42284	1.50582	3.43553
H	-3.33962	2.24177	1.24789
H	-2.80884	2.40374	2.93688
H	-3.09034	0.79267	2.24389

Table S11: Cartesian coordinates of the optimized geometry of isomer **4_I1** at the B3LYP(GD3BJ)/6-31G(d,p)/SMD(THF) level of theory.

Atom	X	Y	Z	Atom	X	Y	Z
C	0.96904	1.27159	2.22239	H	7.03940	-0.30038	-2.88382
C	0.21348	0.13913	1.76698	N	3.48145	-1.00959	1.69918
C	-1.15280	0.05126	2.08669	H	3.52038	0.61773	-2.66634
C	-1.75391	1.05850	2.96431	N	3.46295	-1.53113	-1.01644
C	-0.99469	2.12971	3.44489	H	4.27784	-3.84513	-0.50430
C	0.39907	2.24295	3.08023	C	4.82494	-1.10138	4.05336
C	0.62707	-0.18264	0.39352	P	3.48301	-0.40546	0.18652
C	-2.13442	-0.41395	1.15885	H	4.21275	-0.31662	4.51739
C	-3.10303	1.26439	2.50607	H	5.53916	-1.47291	4.79464
C	-1.57526	3.46592	3.45614	H	5.84648	-1.69991	-0.08818
C	0.68383	3.64389	2.85188	H	5.35679	-0.66494	3.21316
C	1.90018	1.67276	1.18919	N	4.69841	0.68523	0.02048
C	-2.88201	3.66839	3.00514	C	6.55866	-1.57146	0.71965
C	-3.67162	2.54257	2.52032	H	5.37130	3.34463	-0.80029
C	-3.19727	4.81751	2.17392	H	4.73074	3.13739	0.83684
C	-4.49028	2.99486	1.41759	H	7.53613	-1.93410	0.37675
C	-0.28702	-0.57683	-0.54122	C	5.89864	0.81756	0.51952
C	-3.29761	0.38274	1.35235	C	5.64538	3.22532	0.25542
C	-4.18353	4.39889	1.18858	H	6.24482	-2.18554	1.57033
C	-4.13411	0.80068	0.30003	N	6.49104	2.06091	0.47890
C	-1.75056	-0.96419	-0.20027	N	6.66469	-0.17223	1.09357
C	-0.52715	4.40869	3.09599	H	7.86250	2.74368	-0.95710
C	1.54681	4.02244	1.81859	C	7.86650	2.22358	0.00921
C	-0.24775	-0.01773	-1.89932	H	8.34326	1.25551	-0.13539
C	2.15337	3.00934	0.97194	H	6.19344	4.11466	0.57995
C	1.91319	0.62164	0.05496	C	7.70309	0.10397	2.07973
C	1.23199	5.16537	0.98726	H	8.71261	0.00636	1.66128
C	2.25918	3.56800	-0.39472	H	8.46325	2.81203	0.71444
C	1.78347	1.29393	-1.29966	H	7.60834	-0.61589	2.89966
C	-4.67116	2.13170	0.31579	H	7.57977	1.10842	2.48204
C	-0.83182	5.50998	2.28715	H	-2.56907	-5.51635	-0.66770
C	-2.51500	-0.21230	-1.30820	H	-6.21065	-6.47401	-0.35698
C	1.66761	4.87308	-0.37927	H	0.64449	-6.62640	-3.46332
C	2.00041	2.69604	-1.46530	H	0.38165	-5.07401	-4.26085
C	0.75263	0.92327	-2.21129	C	-3.42978	-5.89710	-0.12367
C	0.42522	2.06854	-3.06577	C	0.92857	-5.56820	-3.46130
C	-1.58993	0.22455	-2.33423	C	-5.82966	-5.61604	0.20991
C	-3.65749	0.52408	-1.06923	H	-6.15154	-5.34838	-2.33873
C	-3.98513	1.67721	-1.89303	H	-3.63739	-6.92120	-0.45630
C	1.20771	3.18189	-2.59743	H	-5.61364	-5.95307	1.22920
C	0.66672	4.47397	-2.58442	H	2.00239	-5.50143	-3.66789
C	-0.69859	4.67986	-3.05299	N	-4.60433	-5.07619	-0.36874
C	0.90698	5.35698	-1.46717	H	-3.18428	-5.92714	0.94347
C	-2.19033	5.72483	1.82598	H	0.93794	-1.54839	-3.87923
C	-3.12601	2.06509	-2.92547	H	-6.60135	-4.84910	0.25204
C	0.07248	5.90722	1.21837	H	-1.69594	-3.25914	-4.64168
C	-0.72808	6.37662	0.09442	C	-6.54996	-4.33568	-2.36858
C	-1.91412	1.31676	-3.17638	H	-6.53786	-3.99854	-3.41235
C	-4.59599	2.67493	-1.04101	N	0.59494	-4.98674	-2.16183
C	-4.34164	4.03261	-1.24810	H	-7.58959	-4.35178	-2.02396
C	-0.87896	2.25938	-3.53141	C	0.93584	-2.63769	-3.77547
C	-1.45232	3.59884	-3.51600	H	-1.47661	-1.59560	-4.05209
C	-0.32195	6.09276	-1.21229	H	0.04172	-6.75852	-1.14963
C	-1.31217	5.68189	-2.19779	C	-1.52841	-2.64119	-3.74620
C	-2.85386	3.47626	-3.14624	N	-0.28789	-3.02455	-3.08742
C	-2.12592	6.25208	0.46909	C	-4.55551	-3.84884	-0.97881
C	-4.12301	4.91467	-0.10907	H	1.02771	-3.08182	-4.77566
C	-3.44062	4.43806	-2.31629	C	-0.28904	-3.93471	-2.05034
C	-3.07612	5.85346	-0.47392	C	0.74993	-5.92377	-1.05702
C	-2.65918	5.56457	-1.83976	N	-5.73293	-3.43404	-1.55839
H	1.04897	-4.05275	1.26892	H	1.76547	-6.33108	-1.07138
H	2.70144	-5.19296	2.83504	H	1.79927	-2.92056	-3.17603
H	3.86274	-4.1353	-4.10938	N	-3.53592	-3.02545	-1.02200
H	4.53124	-2.53593	-4.46122	H	-2.37642	-2.75735	-3.07243
C	1.75019	-3.27866	0.96112	N	-1.02521	-3.88188	-0.97507
C	4.64078	-3.41606	-3.82949	H	0.56752	-5.42275	-0.11324
C	3.39421	-4.63177	2.1954	C	-5.89244	-2.02305	-1.88403
H	2.49096	-3.49447	4.31049	P	-2.09918	-2.87701	-0.24087
H	2.03904	-3.4322	-0.07691	H	-5.38722	-1.75383	-2.82036

H	3.53171	-5.20069	1.26869	H	-6.96114	-1.81579	-1.99036
H	5.61758	-3.87506	-4.02078	H	-4.09883	-5.00235	3.93937
N	2.88919	-3.30709	1.86501	H	-5.48655	-1.40365	-1.08702
H	1.24361	-2.32175	1.03182	N	-1.96265	-3.29369	1.32810
H	6.41537	-1.19627	-4.28075	C	-4.73145	-4.19657	3.56963
H	4.35745	-4.55689	2.69942	H	-0.31562	-4.80087	4.35713
H	4.58051	0.64848	-4.10051	H	-0.48994	-4.95063	2.59346
C	3.37777	-2.97632	4.67089	H	-5.05030	-3.57675	4.41722
H	3.06531	-2.28814	5.46511	C	-2.65819	-3.47714	2.41265
N	4.47563	-3.05554	-2.42553	C	-0.59577	-4.27327	3.44027
H	4.07051	-3.70798	5.10099	H	-5.62979	-4.62822	3.11664
C	6.37503	-1.11799	-3.18721	N	-1.98080	-3.84559	3.56464
H	5.15128	1.27429	-2.53065	N	-4.02661	-3.42898	2.54964
H	3.12881	-4.51148	-1.68246	H	-2.31146	-3.81366	5.65808
C	4.54476	0.50317	-3.01274	C	-2.24397	-3.12201	4.81198
N	5.03297	-0.82914	-2.69922	H	-3.16832	-2.55266	4.74359
C	3.44686	-2.14833	2.33683	H	0.08296	-3.42235	3.29921
H	6.7454	-2.04535	-2.7513	C	-4.87288	-2.47687	1.84978
C	4.29216	-1.75939	-2.01448	H	-5.28161	-1.74948	2.56238
C	4.15658	-4.16094	-1.5382	H	-1.43073	-2.40995	5.00309
N	4.00499	-2.21312	3.59246	H	-5.70917	-2.98981	1.36654
H	4.84195	-4.98925	-1.74616	H	-4.31087	-1.93322	1.10312

Table S12: Cartesian coordinates of the optimized geometry of isomer **4_I2** at the B3LYP(GD3BJ)/6-31G(d,p)/SMD(THF) level of theory.

Atom	X	Y	Z	Atom	X	Y	Z
N	-4.86928	-0.85724	-0.73072	C	-2.73188	-5.57630	-1.14694
C	-2.13450	0.49019	-1.40271	N	-6.94077	-0.00902	-1.26236
C	-0.91649	0.16084	-2.07600	H	-2.04743	-6.42785	-1.20631
C	-0.37358	1.01641	-3.04794	H	-0.53986	-1.80443	-4.00190
C	-1.00582	2.28847	-3.30977	H	-3.91982	-1.18169	-2.92747
C	-2.15949	2.63697	-2.59508	N	-3.27931	-2.94768	-0.99187
C	-2.73260	1.72019	-1.63163	H	-2.59964	-5.05809	-0.20166
C	-0.06024	-0.59886	-1.18013	C	-6.32876	1.28278	-1.53670
C	1.06044	1.18796	-3.13189	P	-3.55995	-1.61272	-0.08432
C	1.32681	-0.47297	-1.29345	H	-5.77636	1.28533	-2.48536
C	0.03846	3.27388	-3.53281	H	-7.12169	2.03453	-1.59159
C	-2.31987	3.98542	-2.08482	H	-6.00002	-1.21436	1.84386
C	-3.28991	2.51554	-0.52550	H	-5.63654	1.54924	-0.74150
C	-2.21929	-0.26651	-0.06473	N	-3.62087	-2.22288	1.42239
C	-1.31776	4.93552	-2.29462	C	-5.60448	-0.40675	2.45214
C	-0.11258	4.57442	-3.03082	H	-1.94111	-4.25296	2.84873
C	-0.94160	5.85172	-1.22988	H	-1.30651	-2.76928	2.10325
C	1.00784	5.25073	-2.41319	H	-6.37819	-0.07943	3.15261
C	1.31900	2.59695	-3.41298	C	-3.62302	-1.81832	2.67239
C	0.49900	6.02520	-1.28913	C	-1.65627	-3.20804	3.03308
C	2.39798	3.24681	-2.81548	H	-5.33387	0.43329	1.80175
C	1.85704	0.50205	-2.21188	N	-2.77057	-2.43286	3.55390
C	-2.99958	3.89729	-0.79444	N	-4.46303	-0.88174	3.20997
C	-3.11431	2.00648	0.77599	H	-3.07525	-3.83459	5.07728
C	2.26012	-0.59147	-0.06971	C	-3.14394	-2.75010	4.92892
C	-2.56171	0.71913	1.04748	H	-4.17075	-2.44670	5.12657
C	-0.76270	-0.81865	0.07431	H	-0.84512	-3.19047	3.76679
C	-2.74133	2.93238	1.85334	C	-4.06287	-0.00822	4.31085
C	2.55388	-4.65996	0.12767	P	3.39351	-2.10339	-0.08812
C	-1.73357	0.85441	2.19687	H	-4.69220	-0.16090	5.19513
C	0.01058	-0.58925	1.21538	H	-2.48173	-2.26179	5.65234
C	2.24301	4.59331	-2.30191	H	-4.16090	1.03263	3.98934
C	-2.64212	4.79492	0.23570	H	-3.02145	-0.17870	4.57340
C	2.98739	1.17506	-1.59140	H	5.56929	-5.82794	-0.37764
C	-1.87990	2.20749	2.74749	H	4.07642	-7.29291	1.13036
C	-0.51460	0.17654	2.36508	H	7.40739	-1.17562	-3.88389
C	1.44101	-0.44993	1.20292	H	5.92331	-1.64443	-4.72032
C	1.79896	0.49472	2.20075	C	5.00347	-4.92262	-0.13199
C	3.15897	0.65118	-0.27006	C	6.37997	-0.86601	-4.11150
C	3.26984	2.51338	-1.89745	C	3.81498	-6.29768	1.50900
C	3.64484	3.40596	-0.82976	H	2.26903	-7.18794	-0.33906
C	0.59285	0.86779	2.95632	H	5.58887	-4.31444	0.56625
C	0.47098	2.15859	3.49343	H	4.59642	-5.98354	2.21139
C	1.52722	3.13874	3.27552	H	6.42069	0.06216	-4.69191
C	-0.79817	2.84030	3.36464	N	3.72240	-5.31494	0.43448

C	-1.57875	5.75405	0.01394	H	4.85447	-4.34427	-1.04107
C	3.74461	2.91582	0.47996	H	3.84380	-1.30853	-5.61552
C	-2.50272	4.28349	1.58307	H	2.87141	-6.35996	2.04902
C	-1.37150	4.94876	2.21412	H	3.13200	-3.62669	-2.76750
C	3.48919	1.51474	0.76326	C	1.29670	-6.79146	-0.05217
C	3.01916	4.69689	-1.07578	H	0.62021	-6.89660	-0.91034
C	2.52138	5.43854	-0.00015	N	5.64206	-0.68900	-2.86301
C	2.86679	1.41049	2.08597	H	0.89791	-7.38923	0.77458
C	2.68299	2.76593	2.56832	C	3.47207	-0.87662	-4.67890
C	-0.53746	4.24485	3.08362	H	2.53409	-3.26152	-4.40155
C	0.90745	4.42170	3.02131	H	7.13834	-0.76063	-1.35371
C	3.24272	3.69191	1.59434	C	2.73150	-2.84548	-3.40896
C	-0.79954	5.86774	1.23989	N	3.69594	-1.76680	-3.54323
C	1.24817	6.12800	-0.11334	H	3.95665	0.08288	-4.50706
C	2.64785	4.94147	1.36339	C	4.56401	-1.47453	-2.52564
C	0.58266	6.03965	1.17941	C	6.40770	-0.06756	-1.79208
C	1.45902	5.32098	2.09501	N	1.40115	-5.38235	0.31526
H	-7.09113	-4.12657	-0.36863	H	6.94169	0.79273	-2.20562
H	-8.98982	-2.36019	-0.13597	H	2.39852	-0.69697	-4.77973
H	-3.23590	-6.11921	-3.55194	H	1.79045	-2.49349	-2.97302
H	-2.78178	-4.60819	-4.34064	N	4.47309	-1.89939	-1.29221
C	-6.33943	-3.40284	-0.03244	H	5.73701	0.27455	-1.00695
C	-2.49223	-5.31483	-3.56581	C	0.13507	-4.66635	0.30690
C	-8.18919	-1.87349	0.43354	H	-0.25485	-4.55469	-0.71121
H	-8.31438	-1.37302	-2.09812	H	-0.58728	-5.23049	0.90146
H	-6.09186	-3.62050	1.01302	H	2.80556	-3.96872	2.44484
H	-8.16924	-2.31590	1.43645	H	0.26332	-3.67284	0.72709
H	-1.52049	-5.75023	-3.82396	N	4.29700	-2.09748	1.26227
N	-6.88148	-2.06193	-0.18780	C	2.31872	-3.20801	3.04702
H	-5.43851	-3.52619	-0.62847	H	6.98063	-1.05570	1.77756
H	-1.29417	-3.12972	-4.90839	H	5.64151	0.02281	1.32950
H	-8.41451	-0.81245	0.52929	H	1.93605	-3.67794	3.95717
H	-3.72404	-1.89570	-4.55100	C	4.25366	-1.80310	2.53808
C	-8.11625	-0.30273	-2.07955	C	6.19902	-0.38947	2.16712
H	-7.93377	0.02606	-3.11016	H	1.48351	-2.77711	2.48396
N	-2.46404	-4.66890	-2.25539	N	5.29564	-1.08622	3.07112
N	2.44273	-3.42525	-0.29446	N	3.28350	-2.19164	3.42317
H	-9.00465	0.21753	-1.70502	H	6.90060	-1.65788	4.28957
C	-1.08482	-2.74794	-3.90019	C	5.84946	-1.36274	4.39377
H	-2.62395	-0.61800	-3.98921	H	5.31452	-2.18388	4.86826
H	-3.76214	-5.95502	-1.19254	H	6.67400	0.42838	2.71630
C	-3.19280	-1.49462	-3.67473	C	2.88859	-1.37259	4.56703
N	-2.28919	-2.49533	-3.12260	H	3.08730	-1.88320	5.51646
C	-6.16858	-1.01284	-0.72271	H	5.80223	-0.48278	5.04445
H	-0.44569	-3.45880	-3.37728	H	1.81657	-1.16512	4.49968
C	-2.70167	-3.32256	-2.09629	H	3.41325	-0.41990	4.54993

Table S13: Cartesian coordinates of the optimized geometry of isomer **4_I3** at the B3LYP(GD3BJ)/6-31G(d,p)/SMD(THF) level of theory.

Atom	X	Y	Z	Atom	X	Y	Z
C	3.46325	-5.53713	-0.23859	H	5.49500	3.00422	2.81580
C	2.86330	-5.57807	1.08707	C	-1.16335	3.03964	-3.66828
C	3.21864	-4.63065	2.04885	N	0.03457	3.37204	-2.91175
C	4.18881	-3.59420	1.72707	H	4.46526	3.80182	1.61386
C	4.76415	-3.55552	0.45509	N	3.25691	3.32503	-0.87486
C	4.38888	-4.53652	-0.54630	H	2.76441	6.22866	1.15012
C	1.47324	-5.97740	0.93263	C	3.01154	6.20132	0.08359
C	2.19703	-4.02076	2.89028	H	-1.15949	1.96148	-3.86199
C	3.77151	-2.36702	2.39034	H	4.70017	2.04303	1.53779
C	4.94435	-2.27880	-0.22965	H	-1.21483	3.56368	-4.63258
C	4.34421	-3.86076	-1.83866	H	3.17964	7.22965	-0.26067
C	2.44463	-5.90435	-1.20653	C	1.28825	3.05960	-3.58074
C	4.53047	-1.10421	0.40416	C	4.21391	4.21988	-0.84599
C	3.93256	-1.14141	1.71378	N	4.21104	5.41785	-0.16466
C	3.83121	-0.06283	-0.36093	H	1.26152	2.03915	-3.96624
C	2.87580	-0.13805	1.72352	H	2.12605	3.14575	-2.89214
C	0.49530	-5.39771	1.74873	H	5.30879	1.87912	-1.18136
C	2.55236	-2.62836	3.10578	H	1.44249	3.74588	-4.42754
C	2.87947	0.56602	0.48135	C	5.65067	2.57778	-1.93989
C	1.52770	-1.64394	3.11397	N	5.38047	3.94381	-1.51764
C	0.86205	-4.41224	2.75230	H	5.21849	6.24411	1.46455

C	4.71486	-2.47478	-1.64413	C	5.42240	5.98308	0.41938
C	3.36963	-4.22676	-2.77660	H	5.14902	2.32982	-2.88412
C	-0.78497	-5.00358	1.17837	H	5.74725	6.89309	-0.09973
C	2.40209	-5.25453	-2.44889	H	6.73027	2.46591	-2.07752
C	1.21672	-6.17443	-0.48305	H	6.23052	5.25343	0.39606
C	2.68551	-3.19111	-3.54488	C	6.08642	4.96662	-2.28531
C	1.11847	-4.85480	-3.01171	H	5.60650	5.93661	-2.16657
C	-0.02213	-5.80274	-1.02945	H	6.06106	4.70340	-3.34988
C	1.69183	-0.37048	2.45250	H	7.13432	5.04742	-1.97597
C	4.03359	-1.46251	-2.36738	H	-6.59569	2.21622	-0.53055
C	-0.18728	-3.40003	2.78142	H	-7.70645	1.22128	0.42724
C	1.29710	-3.57943	-3.68414	H	-7.59588	1.32904	-2.48867
C	-0.06581	-5.12361	-2.31901	C	-6.71873	1.24088	-0.05150
C	-1.03013	-5.20233	-0.18187	H	-8.62684	0.07086	-1.78398
C	-1.71031	-4.16499	-0.95834	C	-7.60722	0.29826	-2.11885
C	-1.20103	-3.77144	1.82841	H	-5.96223	1.12831	0.72187
C	0.14895	-2.04765	2.94948	H	-5.71157	2.70737	-3.53230
C	-0.53538	-1.01342	2.18603	N	-6.63134	0.18086	-1.04176
C	-1.11829	-4.12375	-2.27254	H	-4.97537	1.36292	-2.62314
C	-0.94074	-2.88754	-2.91485	C	-4.80743	2.35743	-3.02426
C	-1.36676	-1.65352	-2.26694	H	-3.99540	2.30586	-3.75934
C	0.27646	-2.61279	-3.63693	H	-7.34475	-0.36921	-2.93824
C	3.64422	-0.23491	-1.74834	H	-4.22944	-2.55702	-2.26284
C	-1.53548	-1.37752	1.29276	C	-5.72660	-0.85416	-0.97059
C	2.99868	-1.84219	-3.32944	H	-3.91797	5.26783	-1.59441
C	1.95894	-0.84208	-3.29210	N	-4.49080	3.27732	-1.94111
C	-1.82292	-2.75793	1.07320	N	-4.55464	-0.82725	-0.40166
C	0.40630	0.06794	1.91224	H	-3.99419	4.78521	-3.29582
C	0.34320	0.68291	0.64522	C	-5.13567	-3.03357	-1.89599
C	-2.07129	-2.96254	-0.34115	C	-4.48007	4.68473	-2.31981
C	-1.94855	-1.70111	-1.01946	H	-5.54350	-3.68427	-2.67525
C	0.60921	-1.20904	-3.43172	N	-6.13514	-2.02462	-1.57390
C	-0.41250	-0.59162	-2.62574	N	-3.43691	1.60562	-0.75403
C	-1.82952	-0.56335	0.01769	C	-3.97304	2.80358	-0.76569
C	2.32542	0.13989	-2.28919	H	-4.87335	-3.64901	-1.02668
C	1.62421	1.20987	-0.07357	H	-5.92560	4.41009	-0.17993
C	-0.64508	0.35327	-0.30937	P	-3.51661	0.29008	0.19484
C	1.35597	0.72032	-1.50086	H	-5.49504	5.09080	-2.40056
C	-0.02269	0.30512	-1.60862	C	-7.47419	-2.56341	-1.34077
H	-0.65821	3.57800	4.31484	H	-1.79760	1.37055	2.99394
H	-0.16691	4.79757	3.11437	H	-7.98577	-2.78858	-2.28299
H	1.48537	3.28570	5.88750	C	-5.14861	4.53851	0.57123
C	-0.04087	3.75336	3.43147	N	-4.01961	3.65554	0.30648
H	1.29325	4.93654	5.25733	H	-8.07864	-1.86083	-0.76953
C	1.76849	3.96507	5.07632	N	-3.78532	0.62398	1.78110
H	-0.36773	3.10265	2.62429	H	-2.63049	2.28137	4.27778
H	-2.18137	6.35020	-0.58464	C	-2.22603	1.30408	3.98726
N	1.34239	3.45784	3.77356	H	-4.84405	5.59155	0.59086
H	-0.44343	6.65624	-0.36401	H	-7.39807	-3.49144	-0.76052
C	-1.19768	5.87449	-0.53240	H	-5.57512	4.28817	1.55035
H	-1.16478	5.18525	0.30621	H	-1.43807	1.02974	4.69602
H	2.84701	4.11077	5.09703	C	-3.07072	3.54992	1.40437
H	2.69897	1.50693	4.61438	C	-3.84009	-0.15245	2.85194
C	2.22641	3.23940	2.74494	N	-3.26157	0.28653	4.00691
H	-2.18271	6.24156	-3.11279	H	-2.19271	2.99486	1.07963
N	-0.95812	5.15718	-1.77635	H	-3.50991	3.03441	2.25869
N	1.84269	3.57445	1.53698	H	-5.48880	-2.42877	1.42226
H	-0.57404	6.93947	-2.83181	H	-2.76969	4.56002	1.70123
C	3.65664	1.84616	4.22375	C	-5.67568	-1.71187	2.22628
C	-1.12771	5.99890	-2.96011	N	-4.45661	-1.37570	2.94137
H	4.23264	2.32035	5.02708	H	-3.88863	0.89518	5.89945
N	3.45203	2.75016	3.09544	C	-3.77277	-0.03576	5.33334
N	0.71989	4.02408	-0.70624	H	-6.38689	-2.15296	2.93623
C	-0.01041	4.14741	-1.77522	H	-3.09039	-0.69061	5.88682
H	4.20461	0.96844	3.87126	H	-6.12132	-0.81558	1.80433
H	-2.04699	3.27979	-3.08278	H	-4.74727	-0.51587	5.26005
P	1.87500	3.09434	-0.00990	C	-3.89233	-2.47134	3.72151
H	-0.74826	5.50471	-3.85076	H	-2.87383	-2.23320	4.02298
C	4.59122	2.90170	2.20780	H	-3.85611	-3.36339	3.08976
H	2.16225	5.78288	-0.44916	H	-4.49172	-2.70028	4.61119

Table S14: Cartesian coordinates of the optimized geometry of isomer **4_I4** at the B3LYP(GD3BJ)/6-31G(d,p)/SMD(THF) level of theory.

Atom	X	Y	Z	Atom	X	Y	Z
C	0.26617	6.17366	0.20310	H	-7.15977	0.94005	-1.58396
C	0.00376	5.97130	-1.21852	C	-0.73634	-5.59628	0.45424
C	1.00287	5.45424	-2.04588	N	-1.92964	-4.77214	0.27257
C	2.30783	5.12861	-1.49730	H	-6.38359	-0.60807	-1.19914
C	2.55555	5.31920	-0.13364	N	-3.90447	-1.98710	-1.49908
C	1.52024	5.85985	0.73038	H	-6.76280	-3.10784	0.26335
C	-1.36667	5.51468	-1.35900	C	-6.16662	-3.66932	-0.46308
C	0.67420	4.45137	-3.05330	H	-0.04658	-5.42509	-0.37495
C	2.76580	3.91310	-2.15428	H	-5.39196	0.77245	-1.71680
C	3.29018	4.31931	0.62448	H	-0.98537	-6.66369	0.48511
C	1.61455	5.18284	2.01778	H	-6.63783	-4.64667	-0.62274
C	-0.94526	5.85459	0.93739	C	-2.45880	-4.73455	-1.08308
C	3.72609	3.15577	-0.01470	C	-4.92493	-2.39157	-2.20709
C	3.46102	2.95128	-1.42089	N	-6.08215	-2.96853	-1.73433
C	3.59748	1.85263	0.63737	H	-2.06086	-3.89554	-1.65961
C	3.16875	1.52613	-1.60062	H	-3.54376	-4.65397	-1.06533
C	-1.68704	4.56191	-2.33396	H	-3.82780	-0.39723	-3.50629
C	1.77043	3.51303	-3.13419	H	-2.19391	-5.67102	-1.57837
C	3.31853	0.86243	-0.35874	C	-3.88149	-1.29831	-4.11391
C	1.50431	2.13590	-3.30326	N	-4.88035	-2.20940	-3.57329
C	-0.65517	4.02714	-3.20439	H	-8.10598	-2.45860	-1.67073
C	2.71866	4.24409	1.95535	C	-7.36861	-2.80464	-2.40428
C	0.45118	4.87280	2.72937	H	-2.88096	-1.74929	-4.14773
C	-2.60965	3.48534	-2.00669	H	-7.73225	-3.74632	-2.83268
C	-0.85743	5.19467	2.16968	H	-4.17948	-1.02709	-5.13066
C	-1.94686	5.42854	-0.02764	H	-7.29090	-2.06015	-3.19499
C	0.33727	3.58208	3.38321	C	-5.28917	-3.26799	-4.49519
C	-1.78725	4.14038	2.52373	H	-5.76253	-4.08662	-3.95588
C	-2.83345	4.39349	0.28608	H	-4.40655	-3.66813	-5.01046
C	2.23977	1.12709	-2.59423	H	-5.98689	-2.88989	-5.25007
C	2.60503	2.98711	2.57858	H	5.59262	0.01974	4.14255
C	-0.92752	2.61242	-3.39358	H	6.36673	-1.22874	3.13219
C	-1.04481	3.13171	3.24371	H	4.02110	-1.32816	5.78681
C	-2.75587	3.71745	1.57763	C	5.43071	-0.68801	3.32492
C	-3.15835	3.40308	-0.72004	H	5.44634	-2.29297	5.34476
C	-3.27754	2.12063	-0.05467	C	4.40518	-2.07276	5.08093
C	-2.14464	2.28441	-2.65615	H	5.15473	-0.13435	2.43082
C	0.11748	1.69269	-3.43960	H	7.67764	1.20578	-0.10594
C	-0.00417	0.41060	-2.77043	N	4.35297	-1.59026	3.70450
C	-3.06920	2.33125	1.38842	H	7.55979	-0.49472	0.41547
C	-2.31888	1.34508	2.06625	C	7.03435	0.32122	-0.09834
C	-1.90409	0.12538	1.46917	H	6.12252	0.55377	0.44855
C	-1.30683	1.77127	3.04944	H	3.82853	-2.99068	5.18455
C	3.07967	1.78257	1.94148	H	1.98826	-1.25568	4.45854
C	-1.16686	0.09129	-2.05253	C	3.63643	-2.21442	2.71954
C	1.38443	2.66052	3.31122	H	8.03466	1.09299	-2.70926
C	1.10635	1.25199	3.10729	N	6.70713	-0.01697	-1.47549
C	-2.25328	1.05563	-1.98930	N	4.14670	-2.26745	1.51185
C	1.29221	0.07010	-2.22685	H	8.72722	-0.34638	-1.92934
C	1.36870	-0.58947	-1.00832	C	1.59412	-2.21457	4.12973
C	-2.87343	0.96470	-0.69496	C	7.82794	0.05994	-2.40866
C	-2.33103	-0.26982	0.06436	H	1.48816	-2.88163	4.99311
C	-0.22913	0.82574	2.98157	N	2.45660	-2.79145	3.10214
C	-0.58278	-0.14676	1.95853	N	5.13907	-1.52246	-0.71799
C	-1.09721	-0.61539	-0.80780	C	5.60271	-0.80139	-1.71166
C	2.13807	0.70184	2.26473	H	0.60586	-2.04131	3.69651
C	2.55182	-0.40673	-0.03415	H	5.62027	1.22978	-3.32576
C	0.16603	-0.87104	-0.27709	P	3.72428	-1.92033	-0.02037
C	1.79319	-0.26054	1.30799	H	7.62641	-0.53337	-3.29895
C	0.44474	-0.68690	1.15808	C	1.80554	-3.75359	2.23462
H	-5.07237	0.25955	4.40617	H	5.39002	-4.07623	-0.47256
H	-5.49643	-1.34573	3.75856	H	1.24150	-4.45364	2.85798
H	-7.02661	1.81082	3.46419	C	5.27927	0.35990	-3.88408
C	-5.07975	-0.36134	3.50578	N	5.12181	-0.78292	-2.98929
H	-7.58549	0.13849	3.68239	H	2.55210	-4.30338	1.66697
C	-7.15027	0.84389	2.96467	N	2.94277	-3.06267	-0.89624
H	-4.05882	-0.48951	3.15291	H	5.14295	-4.77956	1.13230
H	-1.61226	-3.33268	4.38611	C	5.03809	-4.96008	0.05774
N	-5.87535	0.31266	2.48912	H	4.30406	0.59772	-4.31603

H	-3.27461	-3.03968	3.81770	H	1.11475	-3.26435	1.54025
C	-2.22255	-3.10322	3.50792	H	5.98381	0.14834	-4.69740
H	-1.92264	-2.13853	3.10318	H	5.67497	-5.80832	-0.21972
H	-7.85075	0.95757	2.13902	C	4.25412	-1.83507	-3.49108
H	-5.95496	2.59127	1.43937	C	2.86579	-4.35898	-0.97210
C	-5.63105	0.02489	1.17109	N	3.66499	-5.26636	-0.31368
H	-1.24134	-5.79896	3.61775	H	3.20029	-1.54159	-3.44695
N	-2.00962	-4.15340	2.51971	H	4.38719	-2.73688	-2.90112
N	-4.94953	-1.05595	0.88293	H	0.52345	-3.29738	-1.58607
H	-2.99157	-5.53110	3.77017	H	4.52008	-2.04103	-4.53347
C	-6.41144	2.29272	0.49811	C	0.89310	-3.98356	-2.34512
C	-2.14411	-5.50249	3.07286	N	1.87910	-4.89656	-1.77470
H	-7.48098	2.53228	0.51494	H	3.38075	-6.60821	1.26390
N	-6.17705	0.87361	0.24825	C	3.16206	-6.53788	0.19128
N	-3.11778	-2.84230	0.97963	H	1.31479	-3.39101	-3.16677
C	-2.37735	-3.88364	1.21052	H	3.63290	-7.39395	-0.30710
H	-5.93892	2.87025	-0.30168	H	0.06189	-4.57676	-2.73202
H	-0.22942	-5.32577	1.37551	H	2.08287	-6.59928	0.05687
P	-3.65963	-1.61375	0.08215	C	2.12738	-6.08358	-2.59304
H	-2.33601	-6.23018	2.28683	H	3.09060	-6.52722	-2.34947
C	-6.27566	0.47056	-1.14283	H	2.14367	-5.80253	-3.65326
H	-5.17962	-3.82638	-0.03833	H	1.34292	-6.83416	-2.44763

Table S15: Cartesian coordinates of the optimized geometry of isomer **4_I5** at the B3LYP(GD3BJ)/6-31G(d,p)/SMD(THF) level of theory.

Atom	X	Y	Z	Atom	X	Y	Z
C	3.32357	3.03902	-1.43887	C	-5.84869	-0.44332	-1.15481
C	2.50477	3.62029	-2.49751	C	-3.58656	-1.80909	3.17530
C	1.74411	2.81028	-3.33671	H	-5.99714	1.00178	1.00456
C	1.75579	1.35615	-3.15046	N	-5.35562	-1.17607	-0.18489
C	2.50702	0.79514	-2.10222	C	-4.76608	-1.69382	-2.95589
C	3.29192	1.64561	-1.22600	H	-7.50893	1.82650	0.57301
C	1.95698	4.87858	-2.00985	C	-3.12413	-6.21709	-1.80005
C	0.39933	3.20468	-3.71509	N	-3.11963	-4.75709	-1.74460
C	0.42598	0.87372	-3.39943	H	-2.85911	-4.05595	2.07454
C	1.99639	-0.30654	-1.33367	N	-5.50171	-0.53776	-2.47055
C	3.29001	1.08434	0.09097	P	-3.93926	-1.66998	0.43339
C	3.25280	3.91361	-0.29726	H	-3.26445	-6.57400	-2.82587
C	0.70669	-0.75023	-1.56148	H	-6.41428	0.41478	-4.14072
C	-0.09700	-0.16216	-2.61395	N	-2.45450	-2.52813	3.44715
C	-0.20913	-1.10482	-0.46197	C	-2.00528	-3.58129	2.55128
C	-1.47115	-0.12878	-2.14733	C	-5.61292	0.57558	-3.41013
C	0.66188	5.26293	-2.37251	H	-3.68368	-1.54938	-2.88157
C	-0.41654	2.01309	-3.75837	H	-2.16630	-6.60373	-1.42769
C	-1.52855	-0.72799	-0.85584	C	-1.88755	-4.13884	-2.21550
C	-1.74226	2.04501	-3.29484	H	-1.38767	-2.76837	5.27013
C	-0.13544	4.42843	-3.25284	H	-5.79447	1.50389	-2.87156
C	2.64826	-0.32105	0.06253	C	-1.45304	-2.08636	4.41422
C	3.20144	1.92745	1.19321	H	-2.04877	-3.08171	-2.41868
C	-0.21988	5.84842	-1.37173	H	-1.68297	-1.08337	4.76817
C	3.19144	3.37754	0.99744	H	-1.34083	-3.19715	1.77056
C	2.41364	5.05312	-0.64167	H	-1.58360	-4.64326	-3.13687
C	2.34953	1.61402	2.32875	H	-1.46391	-4.32781	3.14081
C	2.32214	3.95760	1.98804	H	-4.66417	0.67444	-3.94234
C	1.56854	5.61869	0.31761	H	-1.07961	-4.21606	-1.47875
C	-2.31453	0.95395	-2.53834	H	-0.47842	-2.05264	3.92020
C	1.57987	-0.45033	1.13804	H	5.69408	-4.44733	-0.02861
C	-1.49972	4.46622	-2.78308	H	7.42005	-4.25037	0.36445
C	1.77300	2.85908	2.78179	C	6.49846	-3.71599	0.10902
C	1.51351	5.06233	1.66119	H	6.77666	-4.64654	-2.29277
C	0.22415	6.02346	-0.05860	H	6.23037	-3.06792	0.94068
C	-0.65421	5.70884	1.05983	H	8.37011	-3.90373	-2.04592
C	-1.56059	5.35093	-1.62884	C	7.30635	-3.69071	-2.20384
C	-2.29900	3.30877	-2.81238	N	6.73001	-2.93467	-1.09610
C	-3.16158	2.99757	-1.70178	H	2.90564	-3.59785	3.84617
C	0.14890	5.13655	2.12584	H	4.36108	-2.67129	5.74801
C	-0.37846	4.07194	2.89047	H	1.83517	-6.59555	-1.04731
C	-1.72002	3.58405	2.62976	H	7.18816	-3.14338	-3.13794
C	0.44728	2.93800	3.24007	H	1.93401	-5.15999	1.11127
C	0.26623	-0.91861	0.85118	C	2.59520	-2.54515	3.89009
C	-3.23254	3.85062	-0.59166	C	4.34650	-1.64668	5.35759

C	1.51824	0.46614	2.23075	C	1.43653	-5.75881	-1.63617
C	0.14681	0.54320	2.69354	H	1.98763	-5.71348	-2.57365
C	-2.40332	5.04774	-0.55523	C	6.25630	-1.65221	-1.24352
C	-3.15656	1.54702	-1.51636	N	5.24329	-1.12443	-0.60808
C	-3.24404	1.01718	-0.23359	N	3.73461	-1.65008	4.03176
C	-1.94659	5.23569	0.81098	H	2.04876	-2.30028	2.98175
C	-2.48645	4.14246	1.60899	H	5.37501	-1.29371	5.30491
C	-0.38457	1.73923	3.19088	C	1.20155	-4.60075	0.51359
C	-1.71900	2.13076	2.82349	H	1.93999	-2.40812	4.75549
C	-3.29949	3.30181	0.73854	N	1.59018	-4.51281	-0.88766
C	-0.64969	-0.30062	1.82726	H	3.78937	-1.01798	6.06089
C	-2.59225	-0.33052	0.15385	H	8.79984	-1.66157	-1.65806
C	-3.25803	1.89971	0.89367	H	3.94923	-2.99742	-4.15329
C	-1.93610	0.05883	1.49148	H	4.70050	-2.98089	-2.55023
C	-2.46184	1.31345	1.95735	H	0.38366	-5.96147	-1.85384
H	-6.65908	-3.43798	0.38782	N	4.22421	-1.94142	1.80312
H	-6.47411	-5.15784	0.82197	C	2.55359	-3.58767	-1.23345
C	-5.93587	-4.24645	0.53881	C	4.40132	-1.24493	2.90298
H	-6.87727	-4.57741	-1.86467	H	1.09856	-3.60012	0.93067
H	-5.27553	-3.96154	1.35528	N	3.30487	-3.09839	-0.28039
H	-6.16379	-6.17246	-1.54932	C	3.82841	-2.67008	-3.11665
C	-5.93133	-5.12001	-1.75117	H	0.23567	-5.11097	0.56899
N	-5.16696	-4.51302	-0.66692	C	8.36633	-0.90221	-2.30657
H	-6.10287	-0.48583	3.81687	N	6.91022	-0.88171	-2.17590
H	-5.17098	-1.70351	5.91920	H	6.58737	-0.84187	1.59514
H	-8.90485	0.39638	-1.09224	N	2.64520	-3.30433	-2.56693
H	-5.37879	-5.05171	-2.68717	P	3.94925	-1.68735	0.22059
H	-7.54258	0.13331	1.13019	H	8.67164	-1.09897	-3.33990
C	-5.09762	-0.06125	3.93962	H	1.61281	-4.11088	-4.23666
C	-4.12957	-1.59796	5.59187	N	5.23283	-0.17056	3.04061
C	-8.01330	0.66466	-1.67199	C	6.30247	0.08322	2.08917
H	-7.97816	0.02076	-2.54927	C	1.49578	-3.34202	-3.46377
C	-3.87869	-4.06565	-0.82876	H	3.75270	-1.57740	-3.10020
N	-3.33237	-3.04574	-0.21924	H	8.77210	0.07160	-2.00632
N	-4.10429	-1.08712	4.22343	C	6.28645	0.35914	-2.61676
H	-4.83136	0.47459	3.03089	H	5.78440	0.88469	4.80175
H	-3.66032	-2.57910	5.64242	H	0.58958	-3.53364	-2.89713
C	-6.97643	0.87181	0.54717	C	5.02173	0.89653	4.01379
H	-5.11142	0.64364	4.77558	H	5.21501	0.21805	-2.74084
N	-6.82530	0.47054	-0.84425	H	4.03261	0.81371	4.45738
H	-3.61754	-0.92169	6.28500	H	6.00192	0.80871	1.32670
H	-3.91596	-6.62325	-1.17310	H	6.72726	0.64333	-3.57645
H	-5.02605	-1.85753	-4.00544	H	7.16593	0.47767	2.63483
H	-5.05455	-2.57241	-2.38626	H	1.39424	-2.36829	-3.95263
H	-8.11412	1.70545	-1.99824	H	6.44325	1.17570	-1.90048
N	-4.23102	-1.77778	2.03406	H	5.07578	1.85631	3.49233

Table S16: Cartesian coordinates of the optimized geometry of isomer **4_I6** at the B3LYP(GD3BJ)/6-31G(d,p)/SMD(THF) level of theory.

Atom	X	Y	Z	Atom	X	Y	Z
C	3.48028	3.17807	-0.22180	C	-3.92110	-2.11380	-2.79611
C	3.13516	3.46225	-1.59203	C	-5.55987	-0.30391	1.69168
C	2.72991	2.41637	-2.43422	H	-5.09731	0.15864	-2.67280
C	2.68393	1.05323	-1.94068	N	-4.46180	-1.92164	-1.61410
C	3.04060	0.78085	-0.63344	C	-2.74281	-4.12466	-2.11829
C	3.38356	1.85984	0.24414	H	-5.07265	0.48063	-4.42090
C	2.42915	4.73310	-1.62555	C	-3.10498	-5.51292	3.08115
C	1.62012	2.59295	-3.34625	N	-2.62485	-4.58650	2.05511
C	1.55666	0.36733	-2.58784	H	-5.31607	-2.69312	2.64409
C	2.44208	-0.38561	0.18103	N	-2.94519	-3.03840	-3.06217
C	2.83775	1.57903	1.56314	P	-3.96051	-1.60197	-0.09820
C	2.99002	4.27451	0.61104	H	-2.52444	-6.44183	3.08397
C	1.14044	-0.86459	-0.45216	H	-1.85746	-3.55671	-4.81130
C	0.79097	-0.49558	-1.77576	N	-5.09197	-0.63531	2.94148
C	-0.10016	-0.93755	0.28514	C	-4.82758	-2.00829	3.33288
C	-0.69102	-0.47052	-1.87620	C	-1.85901	-2.80806	-4.01038
C	1.35449	4.90210	-2.50218	H	-2.15701	-3.81069	-1.25339
C	0.89866	1.33293	-3.42779	H	-3.00815	-5.04458	4.06829
C	-1.22659	-0.82586	-0.61640	C	-1.45711	-3.81563	2.46528
C	-0.51723	1.35490	-3.52452	H	-5.25978	0.32723	4.82841
C	0.94977	3.82774	-3.39960	H	-1.93809	-1.81234	-4.44049

C	2.15746	0.32291	1.52145	C	-4.65937	0.36268	3.91111
C	2.41443	2.64134	2.38566	H	-1.02970	-3.29027	1.61700
C	0.16106	5.60562	-2.06012	H	-4.71593	1.35960	3.48115
C	2.46663	4.00794	1.87472	H	-3.75703	-2.22695	3.35381
C	2.33827	5.23377	-0.26140	H	-0.72237	-4.50545	2.88226
C	1.18626	2.48823	3.11791	H	-5.24105	-2.16942	4.33627
C	1.27388	4.70658	2.32704	H	-0.90322	-2.85757	-3.47951
C	1.19045	5.91083	0.16832	H	-1.70882	-3.06978	3.23012
C	-1.31302	0.41336	-2.78559	H	-3.61295	0.16952	4.17405
C	0.94889	0.17936	2.20214	H	5.63830	-4.44489	0.70846
C	-0.49262	3.85017	-3.49810	H	7.34199	-4.21654	0.24030
C	0.47749	3.76097	3.09298	C	6.33636	-3.78447	0.18286
C	0.64602	5.63439	1.48720	H	5.67083	-5.67234	-1.47493
C	0.07860	6.09133	-0.74881	H	6.34015	-2.82366	0.69179
C	-1.15356	5.94778	0.00668	H	7.17225	-5.07399	-2.20965
C	-0.98231	4.93935	-2.66256	C	6.12001	-4.83215	-2.01805
C	-1.20378	2.63867	-3.54037	N	5.97488	-3.62202	-1.21611
C	-2.43343	2.49871	-2.79128	H	4.39682	-1.81137	4.56226
C	-0.80339	5.65792	1.38743	H	6.46621	-0.39807	5.19644
C	-1.56938	4.75281	2.13079	H	1.84489	-6.77404	2.01514
C	-2.71078	4.09222	1.51786	H	5.60292	-4.72014	-2.96984
C	-0.91571	3.78335	2.99655	H	3.11865	-4.65648	3.16872
C	-0.19013	-0.53464	1.64726	C	4.08981	-0.78920	4.30255
C	-2.91553	3.55792	-2.00801	C	6.24034	0.38297	4.46046
C	0.46278	1.28718	3.02597	C	1.28450	-6.10351	1.35367
C	-0.96974	1.30961	2.92790	H	1.45069	-6.41867	0.32411
C	-2.17176	4.80603	-1.94232	C	5.37759	-2.48058	-1.69943
C	-2.49409	1.13474	-2.29617	N	4.65650	-1.63091	-1.01681
C	-3.03867	0.87487	-1.05141	N	5.14421	-0.06640	3.60662
C	-2.25271	5.30719	-0.57778	H	3.19891	-0.83698	3.68025
C	-3.04746	4.37023	0.19475	H	7.13798	0.55849	3.86995
C	-1.66073	2.53460	2.92033	C	2.09448	-4.36088	2.90695
C	-2.77061	2.72596	2.02639	H	3.85183	-0.24868	5.22329
C	-3.45593	3.28906	-0.69928	N	1.75501	-4.73518	1.54004
C	-1.37329	0.21539	2.04440	H	5.98331	1.30083	5.00052
C	-2.58639	-0.30137	-0.16169	H	7.54063	-2.93180	-3.02622
C	-3.46446	1.96876	-0.22820	H	2.11527	-4.42784	-2.76465
C	-2.47114	0.39946	1.20521	H	3.53569	-4.01825	-1.79511
C	-3.11324	1.67518	1.15251	H	0.21768	-6.20321	1.58562
H	-5.77858	-4.36564	-0.94037	N	4.70924	-1.27977	1.70130
H	-6.55131	-4.99478	0.53161	C	2.27273	-3.98488	0.51468
C	-5.74651	-4.35353	0.15500	C	5.28501	-0.23952	2.25200
H	-4.47270	-6.48558	-0.63071	H	2.00590	-3.28203	3.03380
H	-5.93361	-3.33587	0.49228	N	3.23709	-3.15446	0.80829
H	-4.86472	-6.90683	1.04870	C	2.47451	-3.82948	-1.92220
C	-4.23828	-6.26524	0.41790	H	1.39937	-4.85839	3.58684
N	-4.47983	-4.84865	0.67084	C	6.82717	-2.47635	-3.71076
H	-7.67918	0.99203	-0.02521	N	5.54484	-2.25362	-3.04589
H	-8.29758	1.22817	2.38567	H	6.79118	-0.65855	0.18686
H	-5.45289	-1.89260	-5.61027	N	1.75786	-4.25142	-0.73248
H	-3.19113	-6.50742	0.59386	P	3.82438	-1.69355	0.39635
H	-6.33753	-0.56196	-3.72343	H	6.71498	-3.12095	-4.58935
C	-6.73448	1.38988	0.36819	H	0.09229	-5.17501	-1.63063
C	-7.26605	1.16336	2.74957	N	6.06030	0.68772	1.60954
C	-4.42949	-1.94955	-5.22312	C	6.63877	0.41126	0.30551
H	-4.12086	-2.99377	-5.22772	C	0.31826	-4.35721	-0.93909
C	-3.49624	-4.01463	1.14996	H	2.30915	-2.77109	-2.14882
N	-3.30531	-2.76683	0.83498	H	7.23930	-1.51438	-4.03983
N	-6.39860	0.77441	1.64345	C	4.69536	-1.26703	-3.69940
H	-5.94699	1.19866	-0.35453	H	7.15379	2.32753	2.41284
H	-7.22910	0.41846	3.54270	H	-0.20019	-4.53303	-0.00116
C	-5.28130	-0.27032	-3.65285	C	6.15305	2.07991	2.04008
H	-6.84176	2.46948	0.51160	H	3.68323	-1.31584	-3.30399
N	-4.40973	-1.41738	-3.86449	H	5.41732	2.28404	2.81552
H	-6.98558	2.13942	3.16110	H	5.99931	0.76335	-0.51028
H	-4.15484	-5.75339	2.92489	H	4.67532	-1.48541	-4.77124
H	-2.22263	-4.93632	-2.63267	H	7.60733	0.91734	0.24208
H	-3.70558	-4.49307	-1.76955	H	-0.05403	-3.41743	-1.35780
H	-3.77711	-1.37883	-5.89308	H	5.06885	-0.24493	-3.55665
N	-5.31069	-0.93825	0.56505	H	5.93161	2.72516	1.18509

Table S17: Cartesian coordinates of the optimized geometry of isomer **4_I7** at the B3LYP(GD3BJ)/6-31G(d,p)/SMD(THF) level of theory.

Atom	X	Y	Z	Atom	X	Y	Z
C	1.55889	4.66228	1.95860	H	-7.61562	1.07285	-1.55873
C	2.52405	3.57552	2.00856	C	-0.57670	-4.47472	0.18032
C	2.21572	2.40024	2.70486	N	-2.01496	-4.24209	0.10039
C	0.94697	2.26995	3.38452	H	-6.98539	-0.53105	-1.14140
C	0.01664	3.31348	3.34230	N	-4.60190	-1.99893	-1.46589
C	0.32915	4.53111	2.60536	H	-7.12710	-3.19002	0.46065
C	3.17809	3.49511	0.72436	C	-6.73363	-3.76258	-0.38655
C	2.55241	1.09670	2.14552	H	-0.09293	-3.97345	-0.66295
C	0.51195	0.87968	3.24410	H	-5.87689	0.73080	-1.72666
C	-1.39621	3.03383	3.16081	H	-0.33348	-5.54335	0.13848
P	-4.31304	-1.65198	0.11316	H	-7.33810	-4.66726	-0.51502
C	-0.89335	4.99042	1.96307	C	-2.56516	-4.34734	-1.23944
C	1.62038	5.25704	0.62941	C	-5.65929	-2.37336	-2.13727
C	-1.80310	1.70040	3.00808	N	-6.77279	-2.99583	-1.62224
C	-0.86368	0.62107	3.07451	H	-2.28845	-3.48959	-1.86169
C	-2.80931	1.34125	1.99348	H	-3.64964	-4.40425	-1.19398
C	-1.28870	-0.36771	2.07933	H	-4.57405	-0.34796	-3.36842
C	3.46468	2.22763	0.19301	H	-2.17822	-5.26175	-1.70111
C	1.51874	0.14003	2.53848	C	-4.73097	-1.18220	-4.04852
C	-2.53032	0.03627	1.49947	N	-5.70128	-2.11194	-3.48731
C	1.08558	-0.77920	1.53578	H	-8.76273	-2.43453	-1.31495
C	3.21918	1.02163	0.92861	C	-8.11574	-2.75483	-2.14028
C	-1.95522	4.06683	2.31107	H	-3.75978	-1.66016	-4.23194
C	-0.83187	5.57525	0.68897	H	-8.55208	-3.65763	-2.58391
C	3.25256	1.96999	-1.22436	H	-5.12230	-0.80603	-4.99813
C	0.44647	5.69577	0.01076	H	-8.09632	-1.96464	-2.88945
C	2.61669	4.52951	-0.13612	C	-6.23249	-3.08078	-4.44373
C	-1.82688	5.22268	-0.30777	H	-6.66228	-3.93603	-3.92498
C	0.23728	5.44210	-1.40956	H	-5.41955	-3.44563	-5.08384
C	2.42287	4.29064	-1.49709	H	-6.99895	-2.63079	-5.08409
C	-0.31826	-1.10176	1.37919	H	5.13264	-0.12478	4.72987
C	-2.91543	3.71089	1.34088	H	6.34588	-0.85923	3.64955
C	3.02020	-0.15693	-0.05218	H	3.82109	-2.21546	5.70337
C	-1.16746	5.14105	-1.60262	C	5.28175	-0.61233	3.76228
C	1.20779	4.74935	-2.14333	H	5.49914	-2.64785	5.31058
C	2.75627	2.98424	-2.05600	C	4.47395	-2.62036	4.92254
C	1.76950	2.65205	-3.05278	H	4.99635	0.07662	2.97076
C	2.86208	0.59981	-1.39245	H	7.17269	2.80545	1.21114
C	1.74842	-0.92123	0.27770	N	4.44274	-1.80160	3.71343
C	0.75962	-1.17584	-0.71071	H	7.65771	1.10715	1.45062
C	0.80545	3.73820	-3.11136	C	6.93219	1.76875	0.95864
C	-0.55056	3.45370	-3.29563	H	5.93737	1.53432	1.33046
C	-0.99643	2.07365	-3.43875	H	4.17072	-3.64232	4.70075
C	-1.55958	4.17546	-2.53964	H	2.02882	-2.32149	4.08393
C	-3.39156	2.35850	1.21355	C	4.07139	-2.29563	2.48951
C	0.82383	-0.68104	-2.03264	H	7.99222	3.32958	-1.24713
C	-2.83540	4.30411	0.00914	N	6.96693	1.62929	-0.49044
C	-3.23916	3.30311	-0.95364	N	4.73758	-1.89722	1.43206
C	1.89367	0.27973	-2.33409	H	9.01465	2.07220	-0.51768
C	-0.53772	-1.31157	-0.05114	C	1.96792	-3.20763	3.45617
C	-1.69693	-0.92105	-0.68597	C	8.12383	2.24809	-1.13274
C	1.33743	1.32308	-3.18202	H	1.97439	-4.10589	4.08473
C	-0.06654	1.03516	-3.37631	N	3.06363	-3.22036	2.48962
C	-2.61570	3.23357	-2.20652	N	5.85519	-0.37534	-0.30262
C	-2.28566	1.94796	-2.77534	C	6.25705	0.60723	-1.07221
C	-0.37538	-0.19609	-2.64465	H	1.01872	-3.15887	2.91490
C	-3.55338	2.08545	-0.22387	H	5.89275	2.79602	-2.41382
C	-2.92213	-0.38449	0.08635	P	4.52435	-1.28587	-0.05465
C	-1.62042	-0.30828	-1.98574	H	8.29934	1.80682	-2.11242
C	-3.26930	0.84987	-0.77459	C	2.83860	-4.04061	1.31323
C	-2.56453	0.78471	-2.03193	H	6.85102	-2.59347	-0.89782
H	-5.61217	0.38637	4.43928	H	2.35029	-4.96873	1.62266
H	-6.10201	-1.21114	3.81740	C	5.94551	1.97516	-3.12556
H	-7.40104	2.06153	3.42547	N	6.07265	0.70098	-2.42538
C	-5.63596	-0.25292	3.55232	H	3.79290	-4.28336	0.85410
H	-8.10617	0.45150	3.68667	N	4.21186	-2.36496	-1.25065
C	-7.60471	1.09423	2.95353	H	6.79068	-3.46163	0.64127
H	-4.61692	-0.43572	3.21758	C	6.78962	-3.58211	-0.44768
H	-2.06810	-3.24782	4.32269	H	5.01623	1.96754	-3.70328

N	-6.37728	0.44023	2.50763	H	2.20892	-3.52521	0.58077
H	-3.76476	-3.55025	3.86769	H	6.78104	2.14423	-3.81503
C	-2.79425	-3.18418	3.50707	H	7.67433	-4.15702	-0.74201
H	-2.89945	-2.14468	3.20542	C	5.72522	-0.46264	-3.22284
H	-8.28534	1.24362	2.11687	C	4.52408	-3.61872	-1.44997
H	-6.20818	2.68436	1.39506	N	5.60204	-4.28197	-0.91308
C	-6.14831	0.09174	1.20291	H	4.64327	-0.54547	-3.37181
H	-0.74146	-5.21441	3.13383	H	6.07578	-1.36729	-2.73316
N	-2.29985	-3.97560	2.38951	H	1.97458	-3.30644	-1.74968
N	-5.56531	-1.06024	0.95998	H	6.21243	-0.37208	-4.19934
H	-2.39439	-5.71918	3.54437	C	2.38740	-3.85424	-2.59296
C	-6.69315	2.40616	0.46203	N	3.70363	-4.37661	-2.25392
C	-1.76805	-5.28459	2.75643	H	5.85431	-5.81421	0.48544
H	-7.73476	2.74768	0.47079	C	5.56005	-5.69829	-0.56440
N	-6.59652	0.96511	0.25170	H	2.42504	-3.17267	-3.45280
N	-3.79970	-2.92405	0.99741	H	6.24720	-6.29221	-1.17849
C	-2.73115	-3.67553	1.12138	H	1.73334	-4.69552	-2.84246
H	-6.16857	2.91284	-0.35342	H	4.55010	-6.08763	-0.68434
H	-0.17725	-4.04591	1.09533	C	4.23966	-5.33214	-3.22099
H	-1.78621	-5.95859	1.90145	H	5.31979	-5.42024	-3.11683
C	-6.76956	0.53262	-1.12336	H	4.02433	-4.98170	-4.23815
H	-5.71288	-4.05712	-0.15172	H	3.78834	-6.32254	-3.09586

Table S18: Cartesian coordinates of the optimized geometry of isomer **4_I8** at the B3LYP(GD3BJ)/6-31G(d,p)/SMD(THF) level of theory

Atom	X	Y	Z	Atom	X	Y	Z
C	-1.45523	4.46101	-2.32705	C	4.19018	-2.82094	-1.86058
C	-2.17427	3.27699	-2.77391	C	4.37408	-1.62392	3.07303
C	-1.51047	2.28800	-3.49421	H	2.45083	-3.69617	-0.14290
C	-0.09773	2.43695	-3.79798	N	4.61949	-2.50812	-0.66162
C	0.59615	3.57110	-3.36714	C	5.60632	-1.21091	-3.02843
C	-0.09677	4.60337	-2.61395	H	1.88699	-5.06553	-1.12329
C	-3.12838	2.91296	-1.73196	C	9.18079	1.14527	-0.53397
C	-1.75655	0.87790	-3.19770	N	7.77740	0.83716	-0.79988
C	0.51766	1.12131	-3.69064	H	6.58484	-0.34827	2.66523
C	1.93273	3.44141	-2.80711	N	4.51436	-2.16702	-3.01647
C	0.81921	5.10484	-1.59600	P	4.70763	-1.24477	0.35839
C	-1.96575	4.82275	-1.01198	H	9.72841	1.34536	-1.46140
C	2.51743	2.17726	-2.70611	H	4.05905	-2.62374	-5.04144
C	1.79680	1.00880	-3.14200	N	4.79893	-0.50714	3.74018
C	3.26615	1.79454	-1.50109	C	5.91815	0.27952	3.25082
C	2.11478	-0.06725	-2.19484	C	3.62904	-2.10583	-4.17631
C	-3.34243	1.55999	-1.42758	H	5.26413	-0.20764	-2.75048
C	-0.51175	0.16261	-3.33699	H	9.24397	2.03683	0.10226
C	3.08234	0.40226	-1.25784	C	6.94643	2.00232	-1.07110
C	-0.19841	-0.86346	-2.42229	H	4.54691	0.06090	5.77230
C	-2.65870	0.52472	-2.18389	H	2.66011	-2.53801	-3.93598
C	2.07931	4.40566	-1.73345	C	4.03074	0.12628	4.80718
C	0.32547	5.46137	-0.33375	H	5.98626	1.69106	-1.47528
C	-3.52954	1.11119	-0.07125	H	3.04809	-0.33261	4.89110
C	-1.08558	5.29875	-0.03679	H	5.58599	1.11343	2.62337
C	-2.99145	3.85979	-0.65129	H	7.46252	2.63359	-1.80049
C	1.07134	5.08392	0.85782	H	6.46481	0.67817	4.11161
C	-1.21288	4.85212	1.34649	H	3.46976	-1.05689	-4.44205
C	-3.10411	3.41977	0.67433	H	6.75885	2.59273	-0.16491
C	1.12991	-1.02419	-1.89095	H	3.89033	1.18281	4.56015
C	2.79647	4.02389	-0.57969	H	-7.34957	-2.97976	0.95050
C	-2.37161	-0.57075	-1.29270	H	-8.09520	-4.01749	-0.29329
C	0.12291	4.73297	1.90220	C	-7.34544	-3.23922	-0.11393
C	-2.20168	3.92398	1.68638	H	-9.33370	-1.59208	0.19070
C	-3.35565	2.01791	0.96288	H	-6.36347	-3.63742	-0.36174
C	-2.65968	1.67311	2.20925	H	-9.72644	-2.31315	-1.38311
C	-3.11991	-0.37679	0.04672	C	-9.03864	-1.63481	-0.86459
C	-1.15521	-1.21696	-1.38368	N	-7.65533	-2.09095	-0.95139
C	-0.39877	-1.60840	-0.18739	H	-3.25931	-4.59183	2.74475
C	-1.91543	2.83102	2.61553	H	-2.36768	-6.57362	1.35615
C	-0.61842	2.73178	3.17179	H	-6.72374	-0.68503	5.28684
C	0.00281	1.42640	3.24705	H	-9.13573	-0.63710	-1.29005
C	0.40824	3.67865	2.78125	H	-5.05673	-2.34866	4.17187
C	3.45534	2.74827	-0.47930	C	-2.39662	-4.15849	2.22124
C	-0.90517	-1.22238	1.06384	C	-1.94044	-5.92721	0.58040

C	2.28035	4.39009	0.73675	C	-6.38506	0.16006	4.67582
C	2.58351	3.30979	1.64928	H	-7.26176	0.61770	4.22033
C	-2.17991	-0.57797	1.22805	C	-6.67752	-1.36942	-1.59488
C	0.99988	-1.44407	-0.48551	N	-5.41766	-1.29877	-1.25206
C	1.90326	-1.00508	0.48543	N	-2.36252	-4.55048	0.81970
C	-2.02318	0.40417	2.23563	H	-2.44385	-3.07469	2.29829
C	-0.67329	0.29303	2.80707	H	-2.30233	-6.27679	-0.38522
C	1.67186	2.96410	2.65712	C	-4.55602	-1.37482	4.09019
C	1.42683	1.57448	2.94228	H	-1.47921	-4.51213	2.70110
C	0.01859	-0.74260	2.07392	N	-5.46973	-0.33051	3.64867
C	3.29060	2.27820	0.90713	H	-0.84962	-6.02907	0.61065
C	3.17096	-0.18538	0.14400	H	-8.42468	-2.15754	-3.31946
C	1.37711	-0.58902	1.74538	H	-7.78566	1.75469	0.67495
C	3.07695	0.94088	1.20229	H	-7.33208	0.07679	0.32350
C	2.09138	0.59550	2.19182	H	-5.90256	0.89021	5.33454
H	7.11213	-3.36972	-0.01864	N	-4.04148	-3.04298	0.36767
H	8.62841	-2.99648	0.84062	C	-5.72035	-0.20502	2.30709
C	7.67140	-2.58440	0.50228	C	-3.09316	-3.82646	-0.08865
H	8.65744	-2.65834	-1.89884	H	-3.72901	-1.46091	3.38833
H	7.09622	-2.27898	1.37403	N	-5.42802	-1.21542	1.52116
H	9.92342	-1.75139	-1.04699	C	-6.94935	1.05269	0.60662
C	8.89626	-1.69790	-1.42662	H	-4.16376	-1.09509	5.07197
N	7.93341	-1.44271	-0.36030	C	-8.04250	-1.19589	-3.65776
H	3.47365	-4.40965	2.86935	N	-7.09850	-0.64586	-2.68674
H	4.12137	-3.87122	5.30075	H	-4.73545	-3.68545	-2.07479
H	3.80242	-5.85381	-2.72188	N	-6.30929	0.96162	1.90639
H	8.83752	-0.91776	-2.18420	P	-4.60369	-1.52715	0.15705
H	3.44706	-5.16336	-0.26706	H	-8.88533	-0.51601	-3.82284
C	2.85622	-3.52825	3.08955	H	-7.02929	2.59517	3.06182
C	3.87381	-2.80721	5.20288	N	-2.78518	-4.03345	-1.40617
C	3.52733	-4.86651	-3.11206	C	-3.71882	-3.67501	-2.45988
H	4.31385	-4.54354	-3.79189	C	-6.10839	2.23756	2.58660
C	7.17553	-0.29629	-0.30604	H	-6.25418	1.40528	-0.16357
N	5.95348	-0.19993	0.14697	H	-7.53240	-1.35178	-4.61663
N	3.60751	-2.50319	3.79951	C	-6.21858	0.38449	-3.22090
H	2.47457	-3.12549	2.15418	H	-1.38856	-5.37406	-2.28806
H	4.72289	-2.23022	5.56558	H	-5.32255	2.14637	3.33334
C	2.76632	-4.49425	-0.81041	C	-1.44026	-4.36234	-1.86856
H	2.01390	-3.83531	3.71656	H	-5.74047	0.92831	-2.40887
N	3.39287	-3.93132	-1.99787	H	-0.72774	-4.26779	-1.05194
H	3.00400	-2.59662	5.83483	H	-3.51174	-2.67873	-2.86470
H	9.66262	0.32231	-0.00873	H	-6.82135	1.07841	-3.81376
H	6.03370	-1.17913	-4.03459	H	-3.63293	-4.41115	-3.26596
H	6.37400	-1.53253	-2.33064	H	-5.78683	2.97929	1.84978
H	2.59091	-4.96615	-3.67197	H	-5.43175	-0.03497	-3.86132
N	4.66454	-1.93976	1.83459	H	-1.15353	-3.64832	-2.64653

Table S19: Cartesian coordinates of the optimized geometry of isomer **4_I9** at the B3LYP(GD3BJ)/6-31G(d,p)/SMD(THF) level of theory.

Atom	X	Y	Z	Atom	X	Y	Z
C	-0.53875	5.68064	-1.20100	C	3.79690	-3.87978	1.68924
C	-1.24982	4.89622	-2.19956	C	6.19322	0.09880	0.90313
C	-0.53837	4.11939	-3.11396	H	6.24645	-3.16299	1.14616
C	0.91352	4.09755	-3.07292	N	3.58866	-2.65026	1.31101
C	1.59589	4.85393	-2.11980	C	1.59451	-3.80955	2.73982
C	0.85430	5.66035	-1.16192	H	6.90334	-4.81281	1.17890
C	-2.45329	4.36877	-1.57265	C	1.39262	-3.40197	-3.13751
C	-0.99062	2.77120	-3.44036	N	2.53284	-3.48113	-2.22894
C	1.34163	2.73560	-3.37441	H	6.50718	-0.69161	-1.48180
C	2.74590	4.29114	-1.42676	N	2.87549	-4.46900	2.52995
C	1.55259	5.59165	0.11748	P	4.16263	-1.63932	0.14937
C	-1.30887	5.63419	0.03724	H	1.29385	-4.31146	-3.74227
C	3.14967	2.98912	-1.72041	H	2.75497	-6.27921	3.62843
C	2.45254	2.20408	-2.71151	N	6.64563	0.88315	-0.11630
C	3.54952	2.07307	-0.64544	C	6.68634	0.38080	-1.47718
C	2.41754	0.82729	-2.21454	C	3.29298	-5.32484	3.63882
C	-2.88009	3.08011	-1.88863	H	1.66015	-3.00463	3.48384
C	0.16937	1.92937	-3.63550	H	0.48225	-3.27119	-2.54656
C	3.16736	0.74571	-1.00387	C	2.32982	-4.33820	-1.07799
C	0.13610	0.59913	-3.14446	H	7.96845	2.54792	-0.05606
C	-2.15211	2.27463	-2.83918	H	4.36256	-5.52142	3.59145

C	2.72328	4.75732	-0.05203	C	6.89937	2.31427	0.01095
C	0.81622	5.56328	1.30788	H	3.28781	-4.55870	-0.61771
C	-3.37287	2.17657	-0.83973	H	6.49984	2.68881	0.95080
C	-0.64109	5.58617	1.26733	H	5.91850	0.86948	-2.08435
C	-2.49359	4.83695	-0.19813	H	1.88075	-5.27827	-1.41565
C	1.22191	4.66435	2.37218	H	7.66809	0.59604	-1.91528
C	-1.13323	4.70191	2.30605	H	3.08307	-4.82106	4.59090
C	-2.97401	3.97030	0.80233	H	1.66842	-3.87756	-0.33778
C	1.29210	0.01842	-2.49111	H	6.38000	2.82959	-0.80212
C	3.11747	3.87502	0.97351	H	-6.32838	-3.75674	0.85198
C	-2.18716	0.89904	-2.34195	H	-7.62865	-4.10294	-0.31677
C	0.01728	4.13093	2.98917	C	-6.82958	-3.40102	-0.05517
C	-2.26857	3.90918	2.07828	H	-8.36878	-2.65503	1.89649
C	-3.47415	2.65439	0.48267	H	-6.10018	-3.38649	-0.86279
C	-3.01936	1.75354	1.55276	H	-9.47024	-2.45673	0.51849
C	-3.00634	0.83952	-1.17338	C	-8.57769	-2.04861	1.00720
C	-1.07034	0.06260	-2.54939	N	-7.41188	-2.08049	0.12954
C	-0.66144	-0.78827	-1.43997	H	-2.32961	-5.13717	-0.42197
C	-2.30475	2.54299	2.54279	H	-2.44540	-5.97580	-2.88389
C	-1.19999	1.99776	3.22017	H	-3.53710	-4.03743	5.00758
C	-0.77064	0.65716	2.92300	H	-8.78265	-1.02811	1.32743
C	-0.01554	2.81087	3.44866	H	-2.64127	-4.52224	2.62899
C	3.58639	2.54383	0.67987	C	-1.61320	-4.49149	-0.94691
C	-1.39980	-0.87139	-0.26389	C	-2.13243	-5.00336	-3.28530
C	2.34166	3.83568	2.20873	C	-3.47924	-2.95028	4.87741
C	2.31182	2.46847	2.67496	H	-4.44008	-2.52404	5.16155
C	-2.78911	-0.21455	-0.09936	C	-6.78273	-0.93910	-0.30764
C	0.78960	-0.80623	-1.39776	N	-5.50274	-0.80272	-0.53380
C	1.45660	-0.91736	-0.18402	N	-2.13879	-4.00566	-2.21597
C	-2.61898	0.45479	1.28577	H	-1.36533	-3.64956	-0.30232
C	-1.44869	-0.06669	1.93837	H	-2.83006	-4.72973	-4.07445
C	1.15419	1.95902	3.28886	C	-2.20924	-3.53919	2.85957
C	0.70034	0.63283	2.96544	H	-0.70523	-5.06480	-1.15068
C	-0.71015	-0.88206	0.98155	N	-3.18101	-2.65193	3.47992
C	3.05210	1.65624	1.72533	H	-1.13318	-5.11442	-3.71940
C	2.85138	-0.29988	0.05815	H	-9.17477	-1.00395	-1.27017
C	0.69853	-0.90087	1.02009	H	-6.48529	0.09023	3.51062
C	2.63029	0.36800	1.43615	H	-6.36468	-1.01645	2.13183
C	1.40990	-0.11631	2.02159	H	-2.70486	-2.56366	5.54918
H	5.87568	-1.43125	-3.85439	N	-3.68751	-2.77708	-1.03009
H	4.56451	-0.78276	-4.86848	C	-4.00802	-1.90239	2.68371
C	4.80731	-1.17937	-3.87821	C	-3.08515	-2.99861	-2.16957
H	4.83071	-3.38112	-5.22158	H	-1.83603	-3.09579	1.93941
H	4.59909	-0.41738	-3.13279	N	-4.14646	-2.25657	1.42675
H	3.14431	-2.90029	-5.50575	C	-5.83187	-0.26679	2.70881
C	3.84819	-3.26508	-4.74939	H	-1.37518	-3.67530	3.55397
N	3.96834	-2.33840	-3.62844	C	-8.91647	0.04062	-1.10525
H	6.28373	-1.05228	3.61110	N	-7.59322	0.15550	-0.49453
H	8.29715	0.42849	3.26464	H	-5.30556	-1.88389	-2.84385
H	4.96280	-6.40209	0.08186	N	-4.65223	-0.86613	3.30362
H	3.52117	-4.24403	-4.40221	P	-4.13522	-1.55725	-0.04923
H	6.13444	-4.18564	-0.29882	H	-9.68660	0.50386	-0.47913
C	5.80660	-0.11393	3.29668	H	-4.58510	-0.19809	5.32119
C	7.80689	1.07488	2.52711	N	-3.32620	-2.34284	-3.33959
C	4.64850	-6.13838	1.09898	C	-4.52131	-1.53135	-3.50868
H	3.60040	-6.40977	1.21668	C	-4.04204	-0.08598	4.37586
C	3.63460	-2.67701	-2.34489	H	-5.57796	0.58022	2.06180
N	4.39835	-2.24360	-1.35885	H	-8.91327	0.54639	-2.07863
N	6.51045	0.49738	2.17730	C	-6.96841	1.46188	-0.65987
H	4.77371	-0.31953	3.02669	H	-2.68292	-2.84681	-5.30080
H	8.45211	1.13244	1.65227	H	-3.00516	-0.38347	4.51550
C	6.09262	-4.18255	0.79758	C	-2.36376	-2.25609	-4.43355
H	5.82244	0.58569	4.13750	H	-6.12133	1.56446	0.01489
N	4.82731	-4.70279	1.29294	H	-1.38437	-2.59618	-4.10362
H	7.70253	2.07502	2.96115	H	-4.32692	-0.47732	-3.28422
H	1.49250	-2.53830	-3.79085	H	-7.71113	2.23038	-0.42750
H	0.87824	-4.55716	3.09307	H	-4.85966	-1.62071	-4.54549
H	1.23549	-3.37735	1.80895	H	-4.05423	0.96982	4.09019
H	5.25073	-6.72500	1.80282	H	-6.60789	1.61697	-1.68484
N	5.52968	-1.02129	0.74570	H	-2.27400	-1.21028	-4.73602

Table S20: Cartesian coordinates of the optimized geometry of isomer **4_I10** at the B3LYP(GD3BJ)/6-31G(d,p)/SMD(THF) level of theory.

Atom	X	Y	Z	Atom	X	Y	Z
C	0.78926	5.66817	0.37192	C	-6.33755	0.39628	-0.15430
C	1.25305	5.16517	1.66118	C	-3.44368	-2.81583	2.49428
C	0.32173	4.73967	2.61438	H	-5.67668	0.82221	2.31840
C	-1.09677	4.77537	2.30584	N	-5.79971	-0.72564	0.26316
C	-1.54089	5.25410	1.07161	C	-5.97721	-0.07155	-2.52954
C	-0.57360	5.70551	0.08305	H	-7.07360	1.84551	2.71619
C	2.48976	4.44049	1.44731	C	-4.62599	-4.76093	-3.69466
C	0.57445	3.51902	3.36997	N	-4.37694	-3.46346	-3.07133
C	-1.71585	3.59339	2.89628	H	-3.69288	-4.61426	0.63137
C	-2.60810	4.56563	0.36203	N	-6.32203	0.83364	-1.44620
C	-1.05672	5.29563	-1.23143	P	-4.38098	-1.50150	0.27826
C	1.73673	5.23080	-0.64140	H	-5.03589	-4.64680	-4.70410
C	-3.20044	3.43255	0.93308	H	-7.44364	2.42570	-2.30154
C	-2.74441	2.94211	2.20721	N	-2.49042	-3.71564	2.08800
C	-3.49848	2.25942	0.10578	C	-2.63827	-4.46081	0.85037
C	-2.78477	1.47599	2.14280	C	-6.47378	2.23462	-1.82692
C	2.73177	3.25149	2.16996	H	-4.91534	-0.00615	-2.78980
C	-0.68759	2.83103	3.56891	H	-3.68410	-5.31953	-3.76687
C	-3.32706	1.07534	0.88508	C	-3.17213	-2.79229	-3.53761
C	-0.73725	1.42098	3.50921	H	-0.92671	-4.70326	3.13134
C	1.75905	2.80166	3.16066	H	-6.36320	2.87500	-0.95397
C	-2.32329	4.61354	-1.05615	C	-1.14373	-3.74167	2.65159
C	-0.14408	4.88415	-2.20870	H	-3.17626	-1.75229	-3.21898
C	3.32746	2.09813	1.54645	H	-1.01972	-2.94240	3.37765
C	1.27403	4.84151	-1.90019	H	-2.18276	-3.94518	0.00026
C	2.77266	4.45892	0.02800	H	-3.15139	-2.83837	-4.63043
C	-0.44786	3.71976	-3.03178	H	-2.16069	-5.43817	0.97815
C	1.84143	3.66810	-2.55582	H	-5.68068	2.49114	-2.53296
C	3.31566	3.33487	-0.60530	H	-2.26018	-3.26646	-3.15004
C	-1.81213	0.73330	2.84319	H	-0.41873	-3.57915	1.84776
C	-2.61294	3.47765	-1.84319	H	3.73823	-0.68925	-5.19753
C	1.71318	1.35566	3.10766	H	4.35746	-2.25268	-4.61266
C	0.78293	2.98952	-3.26922	C	4.08866	-1.23071	-4.31410
C	2.83746	2.93492	-1.90270	H	5.84861	0.87486	-5.23048
C	3.56637	2.10576	0.15624	H	3.28525	-1.26795	-3.58640
C	3.34068	0.97641	-0.68727	H	6.34023	-0.81162	-5.49300
C	2.66433	0.91035	2.10366	C	6.19265	-0.03771	-4.73117
C	0.49797	0.67926	3.28191	N	5.22246	-0.51771	-3.75258
C	0.19472	-0.45210	2.44820	H	6.94592	-3.43360	-0.80871
C	2.81453	1.46743	-1.91935	H	9.08205	-2.45072	0.12881
C	1.81420	0.80787	-2.66191	H	2.15873	-6.51819	1.49178
C	1.18191	-0.36558	-2.03599	H	7.15000	0.15866	-4.25106
C	0.77119	1.57671	-3.28739	H	3.45489	-4.55772	2.53855
C	-3.25319	2.31811	-1.28042	C	6.81303	-3.66763	0.25548
C	1.12132	-0.85942	1.47370	C	8.68558	-2.36583	1.14777
C	-1.66170	3.04243	-2.86072	C	1.53015	-5.86299	0.87655
C	-1.67989	1.59511	-2.89248	H	1.57031	-6.22579	-0.14893
C	2.39235	-0.20473	1.32924	C	5.48199	-0.57165	-2.41249
C	-1.22980	-0.42155	2.14594	N	4.96170	-1.56344	-1.70752
C	-1.68563	-0.83553	0.91047	N	7.23964	-2.57131	1.11161
C	2.90461	-0.36720	-0.12015	H	5.76507	-3.90226	0.43200
C	1.61770	-0.86121	-0.82431	H	8.91955	-1.37137	1.52460
C	-0.49541	0.87704	-3.10323	C	2.48869	-4.10027	2.28691
C	-0.24408	-0.31535	-2.33648	H	7.41622	-4.54986	0.49371
C	0.64705	-1.25319	0.16782	N	2.03390	-4.49319	0.95913
C	-2.65069	1.13616	-1.91028	H	9.18892	-3.11314	1.77205
C	-2.95051	-0.25054	0.23827	H	7.32096	-0.84183	-0.61917
C	-0.73282	-1.21978	-0.10113	H	2.51822	-4.64434	-3.23399
C	-2.42840	-0.02997	-1.19863	H	3.63403	-3.50708	-2.42038
C	-1.18687	-0.73130	-1.38241	H	0.49973	-5.93434	1.23989
H	-7.29572	-2.92050	0.09611	N	5.18729	-1.32805	1.10102
H	-7.26198	-4.67657	-0.21415	C	2.57226	-3.84418	-0.12883
C	-6.66770	-3.75602	-0.23049	C	6.35572	-1.63246	1.59289
H	-8.04071	-3.01023	-2.30772	H	2.58993	-3.01846	2.34493
H	-5.84749	-3.85790	0.47708	N	3.65707	-3.13496	0.05513
H	-7.51926	-4.63225	-2.80598	C	2.59029	-3.76249	-2.58529
C	-7.17636	-3.60705	-2.62215	H	1.74458	-4.42917	3.01691
N	-6.15881	-3.55118	-1.57799	C	7.16255	0.21914	-0.78704
H	-5.28551	-1.65496	4.47664	N	6.32626	0.40646	-1.95454

H	-3.97836	-3.63955	5.53283	H	5.73940	0.83729	2.20166
H	-9.06983	1.35789	1.12226	N	1.93386	-4.03643	-1.32091
H	-6.78884	-3.18994	-3.55050	P	4.27828	-1.65830	-0.21677
H	-7.27588	0.08026	2.55963	H	8.13310	0.69316	-0.97169
C	-4.23305	-1.39107	4.30738	H	0.28970	-5.27129	-1.85743
C	-3.12366	-3.51427	4.85795	N	6.80026	-0.91299	2.67980
C	-8.30964	1.78301	0.45571	C	6.05794	0.27593	3.07755
H	-8.60861	1.57067	-0.56934	C	0.50158	-4.28522	-1.42706
C	-4.86689	-3.15037	-1.82364	H	2.09936	-2.92371	-3.08757
N	-4.09205	-2.49559	-0.99897	H	6.72177	0.66691	0.10909
N	-3.46378	-2.53293	3.83294	C	6.22252	1.78947	-2.40714
H	-4.18618	-0.58280	3.58209	H	8.47314	-1.02449	3.98114
H	-2.90671	-4.47897	4.40184	H	0.03071	-4.20869	-0.45148
C	-6.74646	0.95835	2.16689	C	7.53486	-1.54965	3.77185
H	-3.79870	-1.04778	5.25029	H	5.38567	1.90167	-3.09236
N	-7.01543	1.17280	0.75193	H	7.75556	-2.58843	3.53286
H	-2.25979	-3.19676	5.45233	H	5.16441	0.02356	3.66218
H	-5.32038	-5.34724	-3.09533	H	6.03530	2.43020	-1.53906
H	-6.57120	0.18991	-3.41043	H	6.71402	0.90186	3.68925
H	-6.21270	-1.09032	-2.23690	H	0.06265	-3.52189	-2.07867
H	-8.28919	2.86816	0.60341	H	6.92138	-1.53466	4.68111
N	-4.35217	-2.24893	1.73314	H	7.14346	2.12542	-2.89796

Table S21: Cartesian coordinates of the optimized geometry of isomer **4_I11** at the B3LYP(GD3BJ)/6-31G(d,p)/SMD(THF) level of theory.

Atom	X	Y	Z	Atom	X	Y	Z
C	-1.49457	-4.92955	-0.32653	C	6.05038	-0.28679	-1.96935
C	-1.71730	-4.64645	1.08631	C	5.34391	0.56029	2.98814
C	-0.63426	-4.60105	1.96483	H	6.10522	-2.15534	-0.18424
C	0.70892	-4.85336	1.47829	N	6.05124	0.28940	-0.78995
C	0.92259	-5.12323	0.12265	C	5.20615	1.67256	-3.16265
C	-0.20127	-5.16248	-0.79744	H	6.99798	-3.33685	-1.16656
C	-2.77200	-3.65026	1.18327	C	4.96033	6.00442	-0.73018
C	-0.55369	-3.55675	2.98455	N	4.55731	4.61758	-0.95523
C	1.60684	-3.95655	2.19537	H	5.23140	3.09839	2.52065
C	2.04448	-4.51859	-0.57734	N	5.53415	0.25915	-3.11103
C	0.23376	-4.57671	-2.06037	P	5.02905	0.95827	0.27675
C	-2.41426	-4.10712	-1.09569	H	4.98083	6.57026	-1.66794
C	2.89521	-3.65487	0.11811	H	5.62326	-0.42538	-5.12173
C	2.68032	-3.37873	1.51573	N	4.58556	1.47417	3.66764
C	3.36703	-2.40917	-0.49681	C	4.39679	2.82194	3.15984
C	3.02169	-1.96704	1.73239	C	5.01283	-0.53688	-4.21808
C	-2.69075	-2.64440	2.15703	H	4.16836	1.85987	-2.86453
C	0.83311	-3.18526	3.14920	H	4.24658	6.48790	-0.05120
C	3.51763	-1.41430	0.51430	C	3.12589	4.43384	-1.15005
C	1.16484	-1.82391	3.34513	H	4.06270	1.56098	5.72681
C	-1.57682	-2.60248	3.07725	H	4.96367	-1.58692	-3.93772
C	1.62284	-4.19416	-1.92709	C	3.71731	1.11799	4.78528
C	-0.66073	-3.79608	-2.80704	H	2.92520	3.44549	-1.55796
C	-3.06072	-1.27531	1.83128	H	3.66059	0.03662	4.88915
C	-2.00031	-3.54907	-2.30303	H	3.47178	2.91238	2.58064
C	-3.19462	-3.31386	-0.15268	H	2.77379	5.19369	-1.85384
C	-0.19076	-2.56477	-3.42134	H	4.35330	3.51047	4.01017
C	-2.36691	-2.17340	-2.63783	H	3.99567	-0.20180	-4.44023
C	-3.52055	-1.98250	-0.45479	H	2.56438	4.53602	-0.21179
C	2.30316	-1.22730	2.69047	H	2.70905	1.48855	4.57623
C	2.07896	-2.99270	-2.51087	H	-7.86197	2.50214	-0.66010
C	-1.24310	-1.19968	3.30852	H	-8.38661	3.85096	0.38087
C	-1.25161	-1.57175	-3.32067	C	-7.62872	3.07150	0.24630
C	-3.09636	-1.40312	-1.71594	H	-9.46178	1.37074	0.94788
C	-3.50537	-0.96121	0.55665	H	-6.66065	3.54867	0.10840
C	-3.27654	0.41936	-0.09844	H	-9.53333	2.51676	2.30193
C	-2.17724	-0.37431	2.58575	C	-8.92734	1.73138	1.83483
C	0.10959	-0.82806	3.45810	N	-7.61521	2.22405	1.42858
C	0.57039	0.37734	2.81217	H	-4.44207	3.62853	-3.79300
C	-2.81410	-0.00920	-1.51071	H	-3.51087	5.97815	-3.27166
C	-1.72544	0.56165	-2.15002	H	-8.06545	-0.82436	-4.18143
C	-0.82653	1.49507	-1.45764	H	-8.82043	0.90282	2.53344
C	-0.93286	-0.22581	-3.08315	H	-6.30251	1.06704	-4.02680
C	2.99623	-2.11667	-1.82368	C	-3.43797	3.41775	-3.40101
C	-0.31609	1.17760	2.07839	C	-2.84773	5.61335	-2.47815

C	1.14232	-2.17140	-3.26876	C	-7.51766	-1.47997	-3.49389
C	1.47085	-0.77825	-3.02876	H	-8.21134	-1.81061	-2.72270
C	-1.71719	0.78095	1.92091	C	-6.44611	1.75936	1.98378
C	1.91560	0.14144	2.31601	N	-5.30025	1.62396	1.36919
C	2.33638	0.72609	1.13503	N	-3.14382	4.20791	-2.21471
C	-2.17004	1.17496	0.62303	H	-3.36770	2.35814	-3.16848
C	-1.04701	1.68798	-0.08110	H	-3.02510	6.21382	-1.58732
C	0.44578	0.17732	-2.93886	C	-5.71851	0.16007	-3.82162
C	0.50999	1.21455	-1.92694	H	-2.70066	3.66253	-4.17096
C	0.10550	1.74599	0.83347	N	-6.40809	-0.73499	-2.90280
C	2.60201	-0.73475	-2.12685	H	-1.81130	5.75518	-2.80398
C	3.37169	0.06906	0.19655	H	-7.80429	2.99079	3.79492
C	1.39743	1.49074	0.35998	H	-7.73982	-1.93999	1.01909
C	2.67951	0.27163	-1.17197	H	-7.36773	-0.22319	0.77838
C	1.61907	1.22353	-1.04987	H	-7.16606	-2.35220	-4.05543
H	7.99726	2.07088	-0.28389	N	-4.49490	2.82106	-0.97665
H	8.41995	3.61138	0.50715	C	-6.32032	-0.48091	-1.55625
C	7.59245	2.94802	0.23288	C	-3.57947	3.75996	-0.99195
H	7.99127	3.71619	-2.21554	H	-4.75661	0.45160	-3.40493
H	7.10216	2.60872	1.14304	N	-5.93268	0.71036	-1.17138
H	7.82597	5.28176	-1.39514	C	-6.97963	-1.23235	0.67602
C	7.27953	4.38967	-1.72353	H	-5.55250	-0.37377	-4.76157
N	6.66024	3.68709	-0.60450	C	-7.26321	2.18339	4.28543
H	7.37756	-1.53112	2.72911	N	-6.52383	1.38940	3.30621
H	7.30155	-0.67178	5.17308	H	-4.75234	4.20246	1.28683
H	8.49050	-2.09350	-2.91724	N	-6.69049	-1.49842	-0.72219
H	6.52397	4.68359	-2.45066	P	-4.85520	1.43683	-0.19705
H	7.85995	-1.97296	-0.40803	H	-7.97485	1.56573	4.84404
C	6.32631	-1.66677	3.01601	H	-7.55301	-3.38062	-1.21100
C	6.24713	-0.37862	5.10615	N	-3.04413	4.39438	0.09691
C	7.46901	-1.87819	-3.25246	C	-3.67447	4.29145	1.40104
H	7.51592	-1.05712	-3.96596	C	-6.57230	-2.90951	-1.07741
C	5.29723	3.56155	-0.47202	H	-6.08766	-1.34004	1.30352
N	4.66141	2.54193	0.04159	H	-6.56017	2.63044	4.99929
N	5.80312	-0.50461	3.72051	C	-5.44050	0.59469	3.86915
H	5.73700	-1.85037	2.12019	H	-1.59764	5.94231	0.25971
H	6.16329	0.65342	5.44279	H	-5.99094	-3.02033	-1.99110
C	6.92300	-2.28130	-0.89048	C	-1.65839	4.85535	0.13060
H	6.25031	-2.53395	3.67821	H	-5.10612	-0.14718	3.14731
N	6.65582	-1.50966	-2.09635	H	-1.14140	4.56848	-0.78251
H	5.66788	-1.02148	5.77796	H	-3.31112	3.42232	1.95946
H	5.94549	6.04820	-0.26915	H	-4.57720	1.21245	4.14778
H	5.34803	2.02872	-4.18722	H	-3.45066	5.20256	1.96476
H	5.87221	2.22194	-2.50387	H	-6.04250	-3.42955	-0.27520
H	7.07721	-2.76803	-3.75694	H	-1.14606	4.37529	0.97000
N	5.69905	0.63692	1.72676	H	-5.81391	0.08750	4.76345

Table S22: Cartesian coordinates of the optimized geometry of isomer **4_I12** at the B3LYP(GD3BJ)/6-31G(d,p)/SMD(THF) level of theory.

Atom	X	Y	Z	Atom	X	Y	Z
C	-3.01731	-2.23645	-1.92383	C	5.94032	0.09939	-2.15218
C	-2.23523	-3.40253	-2.22522	C	5.34858	0.68730	2.86129
C	-1.06997	-3.34436	-3.02474	H	6.15691	-1.86124	-0.46862
C	-0.60478	-2.04619	-3.46228	N	5.93652	0.60041	-0.93862
C	-1.31129	-0.88428	-3.14068	C	4.95994	2.06864	-3.20719
C	-2.52772	-0.95971	-2.30697	H	7.06371	-2.93949	-1.54870
C	-2.25576	-4.29492	-1.06876	C	4.45391	6.24740	-0.63716
C	0.10534	-4.11427	-2.65090	N	4.15118	4.84075	-0.89157
C	0.85460	-2.04004	-3.40837	H	5.05330	3.23461	2.52712
C	-0.61756	0.31814	-2.77503	N	5.39932	0.68738	-3.26308
C	-2.61846	0.20407	-1.51375	P	4.90900	1.18245	0.17522
C	-3.46120	-2.35878	-0.52962	H	4.41276	6.83772	-1.55905
C	0.79466	0.32343	-2.72210	H	5.35658	0.09567	-5.30273
C	1.52517	-0.87221	-3.03558	N	4.56244	1.51747	3.61398
C	1.48977	0.96922	-1.62549	C	4.25306	2.86383	3.16324
C	2.67766	-0.93727	-2.11464	C	4.82620	-0.08796	-4.36127
C	-1.13317	-5.06075	-0.74521	H	3.93739	2.14739	-2.82022
C	1.29809	-3.33436	-2.93754	H	3.71957	6.65759	0.06751
C	2.69953	0.22686	-1.31813	C	2.73231	4.55151	-1.04942
C	2.40221	-3.38250	-2.05420	H	4.18442	1.48049	5.70680
C	0.06839	-4.99252	-1.55628	H	4.85262	-1.15065	-4.13058

C	-1.39832	0.95919	-1.73325	C	3.79667	1.06291	4.77013
C	-3.31347	0.18983	-0.16168	H	2.19232	4.61561	-0.09543
C	-0.73746	-5.20490	0.65303	H	3.80957	-0.02395	4.82745
C	-3.53972	-1.26552	0.31879	H	3.31687	2.90023	2.59563
C	-3.01438	-3.64495	-0.01980	H	2.59268	3.55003	-1.44915
C	-2.36475	0.70725	0.94094	H	4.16461	3.51222	4.04053
C	-3.08555	-1.40793	1.68030	H	3.77850	0.19964	-4.48809
C	-2.64871	-3.79562	1.32167	H	2.30551	5.28234	-1.74282
C	3.15100	-2.21046	-1.69672	H	2.75760	1.38270	4.65171
C	-0.71340	1.62378	-0.70207	H	-7.72576	2.66638	0.56532
C	1.20748	-5.05071	-0.65872	H	-8.14786	4.01020	-0.52701
C	-2.36820	-0.20455	2.05849	C	-7.34059	3.34246	-0.20579
C	-2.68592	-2.64731	2.19929	H	-7.26196	4.66341	2.03317
C	-1.48045	-4.59096	1.66449	H	-7.02765	2.74195	-1.05723
C	-0.79576	-3.93229	2.76707	H	-7.15388	5.98214	0.85000
C	0.71085	-5.19888	0.70649	C	-6.60954	5.13840	1.29077
C	2.34442	-4.27540	-0.89892	N	-6.23431	4.14764	0.28777
C	3.01789	-3.61956	0.20305	H	-7.67126	-1.73715	-1.72104
C	-1.54072	-2.72818	3.09854	H	-7.89457	-1.43265	-4.27438
C	-0.85916	-1.56944	3.46839	H	-8.10590	-0.70946	3.99526
C	0.59307	-1.56305	3.52173	H	-5.72346	5.51477	1.79984
C	-1.29127	-0.27680	2.94889	H	-7.79216	-1.21694	1.45724
C	0.72592	1.62789	-0.64833	C	-6.65353	-1.93645	-2.08315
C	2.55519	-3.77337	1.51395	C	-6.84352	-1.14630	-4.39931
C	-1.21569	1.42954	0.67218	C	-7.03083	-0.57056	4.16141
C	-0.07300	1.34447	1.55917	H	-6.89162	0.38113	4.67130
C	1.37170	-4.57860	1.76978	C	-4.92381	3.87315	-0.02750
C	3.49044	-2.32976	-0.27336	N	-4.44128	2.71490	-0.39042
C	3.49172	-1.23634	0.57740	N	-6.23884	-0.96411	-3.08338
C	0.60165	-3.92637	2.81874	H	-5.97067	-1.91452	-1.23794
C	1.30962	-2.71598	3.20372	H	-6.80973	-0.21664	-4.96527
C	-0.11187	0.51716	2.68620	C	-6.84645	-1.54082	1.91138
C	1.05117	-0.26674	3.03483	H	-6.62925	-2.93189	-2.53550
C	2.51685	-2.62479	2.39135	N	-6.34159	-0.56979	2.87263
C	1.13141	1.44405	0.75966	H	-6.33884	-1.93212	-4.97202
C	3.29412	0.21738	0.08120	H	-5.45351	6.30198	-0.70526
C	2.94051	-1.38182	1.90125	H	-4.46181	3.15638	3.93773
C	2.26320	0.72979	1.11142	H	-5.19582	3.04892	2.32658
C	2.19030	-0.18328	2.22642	H	-6.67476	-1.37829	4.80993
H	7.77334	2.52192	-0.43384	N	-5.83088	0.56444	-1.41732
H	8.13025	4.04573	0.41890	C	-5.67150	0.52913	2.39325
C	7.33489	3.34652	0.13919	C	-5.68096	0.21982	-2.67604
H	7.60390	4.25646	-2.27533	H	-6.11483	-1.69474	1.12042
H	6.89835	2.93441	1.04683	N	-5.81276	0.84717	1.13075
H	7.36106	5.76875	-1.37819	C	-4.48955	2.58211	3.00701
C	6.86499	4.85730	-1.73195	H	-7.01015	-2.48731	2.43402
N	6.32994	4.06561	-0.62860	C	-4.40877	6.26865	-0.40098
H	7.47656	-1.25435	2.39509	N	-4.05478	4.93620	0.08430
H	7.51312	-0.48064	4.85597	H	-5.56043	2.82295	-2.83335
H	8.43033	-1.53397	-3.27736	N	-4.92832	1.22974	3.30304
H	6.06891	5.13054	-2.42286	P	-4.92947	1.15899	-0.19732
H	7.88939	-1.57956	-0.74467	H	-4.23801	7.03059	0.36739
C	6.45646	-1.47576	2.73634	H	-4.83961	0.96341	5.41309
C	6.43509	-0.27968	4.87810	N	-5.08093	0.96192	-3.66012
C	7.38772	-1.35005	-3.56293	C	-4.79046	2.36937	-3.45386
H	7.36699	-0.49024	-4.23072	C	-4.34237	0.62682	4.49590
C	4.98448	3.83038	-0.47007	H	-3.49217	2.59649	2.55279
N	4.44257	2.74732	0.02075	H	-3.79361	6.51656	-1.27525
N	5.89748	-0.38563	3.52347	C	-2.62456	4.66134	0.09451
H	5.83128	-1.66490	1.86639	H	-4.69048	0.58988	-5.71385
H	6.29044	0.72516	5.27121	H	-4.39751	-0.45891	4.43333
C	6.94999	-1.90554	-1.21102	C	-4.29326	0.34094	-4.72298
H	6.48021	-2.37347	3.36068	H	-2.41892	3.77370	0.68888
N	6.60296	-1.08279	-2.36064	H	-4.27505	-0.73945	-4.60097
H	5.96459	-0.99936	5.55685	H	-3.81986	2.51786	-2.96929
H	5.44212	6.35421	-0.19298	H	-2.11201	5.52032	0.53761
H	4.99482	2.48751	-4.21683	H	-4.78556	2.86497	-4.42983
H	5.63053	2.63588	-2.56853	H	-3.28787	0.90920	4.54840
H	7.01809	-2.22956	-4.10114	H	-2.22679	4.49513	-0.91491
N	5.64004	0.84607	1.59476	H	-3.26205	0.70199	-4.65842

Table S23: Cartesian coordinates of the optimized geometry of isomer **4_I13** at the B3LYP(GD3BJ)/6-31G(d,p)/SMD(THF) level of theory.

Atom	X	Y	Z	Atom	X	Y	Z
C	2.28386	-1.49145	-3.19673	C	-4.76884	3.38213	0.08486
C	2.83812	-0.35162	-2.45337	C	-5.55809	-0.73018	2.96878
C	2.18503	0.89458	-2.52111	H	-3.56905	3.02691	2.36054
C	0.93047	0.99372	-3.21878	N	-5.31443	2.34466	0.67336
C	0.38893	-0.09404	-3.93295	C	-5.58635	2.83999	-2.15250
C	1.08710	-1.36447	-3.90042	H	-3.16331	4.73447	2.62881
C	3.50156	-0.83278	-1.28740	C	-9.00119	-0.64837	-2.71133
C	2.04709	1.64768	-1.26330	N	-7.66067	-0.20596	-2.33364
C	0.03841	1.84827	-2.43784	H	-7.32088	-1.55653	1.26812
C	-1.02964	-0.37965	-3.85009	N	-4.74676	3.61908	-1.26168
C	0.09459	-2.43324	-3.83559	P	-5.32525	0.72132	0.62529
C	2.55297	-2.67438	-2.41586	H	-9.32622	-0.18568	-3.64961
C	-1.87831	0.44913	-3.10927	H	-4.02807	5.28325	-2.37124
C	-1.32704	1.57082	-2.38949	N	-5.84553	-2.03362	2.67037
C	-2.92375	-0.12740	-2.25885	C	-6.65078	-2.37739	1.51071
C	-2.06104	1.67409	-1.12119	C	-3.66807	4.35109	-1.92072
C	3.62736	0.02371	-0.03407	H	-5.08970	1.91440	-2.46478
C	0.74071	2.26821	-1.24409	H	-8.99948	-1.73680	-2.84691
C	-3.08467	0.68435	-1.09615	C	-6.58217	-0.87549	-3.04785
C	0.02300	2.38527	-0.03360	H	-5.86664	-3.73967	3.93968
C	2.65431	1.22451	-0.08772	H	-2.87272	4.57232	-1.21226
C	-1.21653	-1.82364	-3.82304	C	-5.17844	-3.16353	3.31010
C	0.36163	-3.58839	-3.08839	H	-5.64289	-0.35125	-2.88958
C	3.08678	-0.73403	1.19890	H	-4.34228	-2.81609	3.91391
C	1.61135	-3.70269	-2.35348	H	-6.03001	-2.58874	0.63326
C	3.26122	-2.23680	-1.20992	H	-6.82267	-0.87798	-4.11507
C	-0.68467	-4.15093	-2.25458	H	-7.24390	-3.26568	1.75021
C	1.35132	-4.37862	-1.09361	H	-3.24748	3.72206	-2.71056
C	3.04995	-2.92557	0.00214	H	-6.45307	-1.91323	-2.71405
C	-1.39806	2.15002	0.02670	H	-4.78317	-3.82383	2.53318
C	-2.23204	-2.36103	-3.00891	H	8.29092	2.03017	0.05506
C	1.90174	1.29357	1.13023	H	9.24347	2.02677	1.56190
C	-0.06893	-4.63749	-1.02201	C	8.24592	1.86821	1.13759
C	2.04768	-3.95541	0.05867	H	8.12389	4.43968	0.80082
C	2.90287	-2.10688	1.21633	H	7.95268	0.83544	1.31396
C	1.86072	-2.69329	2.03680	H	8.55454	4.34396	2.52010
C	2.17427	0.10325	1.92550	C	7.74861	4.17406	1.79626
C	0.63326	1.89574	1.18648	N	7.31391	2.78124	1.78102
C	-0.40903	1.30840	2.00656	H	7.68429	-2.28756	-1.84387
C	1.32333	-3.83068	1.32216	H	8.50359	-4.10020	-0.20426
C	-0.04517	-4.11306	1.38415	H	7.05301	2.71556	-4.67890
C	-0.92658	-3.25068	2.15808	H	6.91197	4.82873	2.03576
C	-0.76161	-4.53620	0.19164	H	7.19311	0.42274	-3.46705
C	-3.14055	-1.52286	-2.26968	C	6.74957	-2.75917	-1.51246
C	-0.14888	0.17336	2.78114	C	7.57394	-4.09286	0.37720
C	-1.94752	-3.55619	-2.21507	C	6.05384	2.93559	-4.28472
C	-2.65682	-3.43258	-0.95839	H	6.08281	3.92459	-3.83023
C	1.17628	-0.43862	2.74857	C	6.05007	2.39565	2.16228
C	-1.66445	1.43887	1.28855	N	5.36076	1.40276	1.66450
C	-2.61724	0.43600	1.36993	N	6.73736	-2.98425	-0.07427
C	1.01039	-1.87297	2.79707	H	5.91425	-2.12105	-1.79187
C	-0.40925	-2.15998	2.85556	H	7.83524	-3.97264	1.42736
C	-2.07365	-3.91207	0.22246	C	6.11640	0.56853	-3.62503
C	-2.18444	-3.13518	1.43391	H	6.64538	-3.72570	-2.01390
C	-1.12536	-0.88302	2.84542	N	5.69888	1.92379	-3.29271
C	-3.37099	-2.16790	-0.97042	H	7.07550	-5.05890	0.24142
C	-3.59521	0.10924	0.21762	H	7.28407	3.37327	4.20332
C	-2.31415	-0.75768	2.11014	H	4.71033	5.07169	-0.52350
C	-3.51062	-1.43511	0.20219	H	5.70522	3.73543	0.08462
C	-2.85069	-1.89900	1.38847	H	5.34810	2.94781	-5.12235
H	-7.98594	2.45108	1.06401	N	6.35215	-0.74080	0.25739
H	-9.53700	1.57297	1.04972	C	5.44668	2.22521	-1.97959
C	-8.47289	1.50585	0.79900	C	6.35797	-1.95649	0.75156
H	-9.01286	3.11414	-1.16427	H	5.57097	-0.14603	-3.01197
H	-8.02442	0.71333	1.39448	N	5.90952	1.41581	-1.05606
H	-10.20248	1.82877	-1.45483	C	4.77564	3.98461	-0.41836
C	-9.13470	2.07458	-1.49125	H	5.88504	0.38613	-4.67824
N	-8.34606	1.20944	-0.61954	C	6.22829	3.61420	4.31404
H	-5.25332	1.58456	4.72431	N	5.47122	3.16320	3.14788

H	-6.21886	-0.40274	6.05110	H	6.72233	-0.48877	2.84866
H	-4.88547	6.26891	1.41034	N	4.76722	3.38717	-1.74150
H	-8.78642	1.99471	-2.51993	P	5.40246	0.58269	0.24089
H	-4.82770	4.15773	2.90246	H	6.12353	4.69431	4.46338
C	-4.51161	0.79287	4.55285	H	4.13375	4.90400	-3.08976
C	-5.73559	-1.15209	5.41247	N	6.06058	-2.30099	2.04168
C	-4.35103	5.78224	0.58570	C	6.05380	-1.30778	3.10193
H	-4.92855	5.93358	-0.32481	C	3.79100	3.95186	-2.66818
C	-7.39442	0.34182	-1.10207	H	3.93252	3.63638	0.18864
N	-6.27516	0.01600	-0.50847	H	5.85682	3.10445	5.21191
N	-5.14047	-0.48621	4.25596	C	4.03837	3.03769	3.37586
H	-3.86853	1.09095	3.72768	H	6.17660	-4.18661	3.01804
H	-6.49663	-1.86337	5.09598	H	3.58499	3.24904	-3.47258
C	-3.93544	4.04699	2.27182	C	5.48522	-3.59046	2.41093
H	-3.90425	0.67686	5.45506	H	3.51329	2.95227	2.42694
N	-4.21618	4.35709	0.87742	H	5.20793	-4.14899	1.51945
H	-4.98185	-1.67820	6.00842	H	5.05193	-0.89867	3.26961
H	-9.71994	-0.40741	-1.92986	H	3.69313	3.93378	3.89975
H	-5.81185	3.44235	-3.03698	H	6.40330	-1.78265	4.02441
H	-6.51678	2.59557	-1.64903	H	2.85610	4.12589	-2.12756
H	-3.37518	6.26719	0.47404	H	3.79189	2.15749	3.98410
N	-5.67806	0.29223	2.15828	H	4.57601	-3.41332	2.99363

Table S24: Cartesian coordinates of the optimized geometry of isomer **4_I14** at the B3LYP(GD3BJ)/6-31G(d,p)/SMD(THF) level of theory.

Atom	X	Y	Z	Atom	X	Y	Z
C	1.94260	3.65509	2.08134	C	-6.55430	1.45192	0.20905
C	2.46804	2.39349	2.57169	C	-4.68638	-2.99442	1.80414
C	1.67298	1.58031	3.39254	H	-5.70034	1.05736	2.61759
C	0.33160	1.99587	3.74977	N	-6.35540	0.16196	0.35243
C	-0.17244	3.21154	3.27567	C	-6.53892	1.40789	-2.23676
C	0.65426	4.06207	2.42832	H	-6.55438	2.43160	3.35338
C	3.29132	1.81384	1.53054	C	-6.60192	-3.00004	-4.51978
C	1.64935	0.14978	3.16988	N	-5.97300	-1.99069	-3.67035
C	-0.51346	0.80430	3.74760	H	-5.25535	-4.02800	-0.49447
C	-1.53813	3.30212	2.79766	N	-6.50383	2.12925	-0.97715
C	-0.21137	4.68300	1.43467	P	-5.24297	-0.96884	0.00895
C	2.44458	3.85850	0.72508	H	-7.02932	-2.55131	-5.42310
C	-2.34285	2.15016	2.79580	H	-6.99755	4.14894	-1.42272
C	-1.82397	0.89664	3.27281	N	-3.98989	-3.98612	1.16867
C	-3.19902	1.84341	1.65186	C	-4.23932	-4.30551	-0.22605
C	-2.37946	-0.16161	2.41134	C	-6.15067	3.54143	-1.08330
C	3.29324	0.44110	1.34077	H	-5.53165	1.17853	-2.60203
C	0.30742	-0.34453	3.42740	H	-5.85172	-3.74028	-4.82472
C	-3.27739	0.41792	1.48156	C	-4.65578	-1.56328	-4.12024
C	-0.23228	-1.34644	2.59382	H	-2.95292	-5.66158	1.96743
C	2.43987	-0.38918	2.14330	H	-5.79486	3.91447	-0.12528
C	-1.56424	4.20728	1.66871	C	-2.79240	-4.60331	1.72984
C	0.26501	4.87535	0.13381	H	-4.36096	-0.65088	-3.60655
C	3.52549	-0.21967	-0.03726	H	-2.47842	-4.07157	2.62560
C	1.61359	4.47503	-0.23111	H	-3.54824	-3.77974	-0.89363
C	3.28486	2.74158	0.38206	H	-4.70376	-1.37264	-5.19631
C	-0.60497	4.58045	-0.99651	H	-4.11463	-5.38469	-0.36333
C	1.57982	3.93946	-1.57466	H	-5.33711	3.64377	-1.80738
C	3.22557	2.19314	-0.90670	H	-3.88786	-2.32517	-3.93159
C	-1.59424	-1.30826	2.13808	H	-1.98463	-4.53093	0.99508
C	-2.39995	3.90556	0.57564	H	7.96583	-2.39742	-1.12835
C	1.89283	-1.44329	1.30372	H	9.39749	-2.13501	-0.09952
C	0.21412	4.00500	-2.05631	C	8.38018	-1.79089	-0.31523
C	2.37243	2.82802	-1.90886	H	9.36246	-0.77400	-2.49132
C	3.28834	0.77800	-1.16131	H	7.76627	-1.94433	0.56981
C	2.38126	0.50413	-2.21310	H	10.44238	-0.06224	-1.27519
C	2.41796	-1.30032	-0.02746	C	9.42276	-0.04144	-1.67762
C	0.59679	-1.92351	1.53468	N	8.42936	-0.37930	-0.66327
C	-0.25266	-2.19870	0.40441	H	4.61499	-5.03647	0.83945
C	1.83985	1.77315	-2.72840	H	5.20554	-5.43084	3.32252
C	0.53062	1.83698	-3.21170	H	5.16680	-4.13456	-4.82086
C	-0.30217	0.65352	-3.24229	H	9.22289	0.94688	-2.08892
C	-0.30168	2.98621	-2.86517	H	4.73219	-4.53361	-2.31312
C	-3.25112	2.74421	0.57953	C	3.84486	-4.46321	1.37288
C	0.23276	-2.01051	-0.89623	C	4.76447	-4.48055	3.64718

C	-1.89798	4.10297	-0.78196	C	4.71852	-3.13394	-4.81119
C	-2.43251	3.04342	-1.61855	H	5.42982	-2.44173	-5.25880
C	1.59002	-1.53966	-1.11288	C	7.47969	0.51598	-0.23066
C	-1.60291	-1.79201	0.75117	N	6.26063	0.22794	0.14551
C	-2.42938	-1.24587	-0.21645	N	4.40588	-3.69027	2.47174
C	1.58738	-0.66673	-2.29431	H	3.35097	-3.80022	0.66589
C	0.22554	-0.55352	-2.73827	H	5.50400	-3.95685	4.25056
C	-1.64735	2.49754	-2.64454	C	3.93335	-3.83368	-2.59263
C	-1.64194	1.06425	-2.85413	H	3.10775	-5.15949	1.78318
C	-0.61218	-1.40688	-1.89398	N	4.43132	-2.75236	-3.43072
C	-3.25391	2.18929	-0.78762	H	3.88948	-4.70146	4.26824
C	-3.52473	-0.20555	0.11258	H	9.81773	1.37532	0.43862
C	-1.90531	-1.00330	-1.53350	H	6.48015	1.00450	-4.07372
C	-3.28320	0.82424	-1.01369	H	6.91036	0.07121	-2.62904
C	-2.44067	0.25559	-2.03002	H	3.81002	-3.15745	-5.42272
H	-8.42020	-1.49550	-0.12736	N	5.30814	-2.22337	0.94600
H	-8.92365	-3.06260	-0.81210	C	5.00358	-1.66097	-2.82780
C	-8.08647	-2.36401	-0.70564	C	4.99096	-2.47782	2.19154
H	-9.32765	-0.83555	-2.40184	H	3.49095	-3.42720	-1.68637
H	-7.29121	-2.85649	-0.14992	N	5.37109	-1.76876	-1.57299
H	-9.29302	-2.37142	-3.29135	C	6.08360	0.49766	-3.18959
C	-8.67616	-1.53752	-2.93578	H	3.16649	-4.37816	-3.15094
N	-7.62235	-1.98564	-2.03134	C	9.19561	2.24494	0.23387
H	-6.20921	-1.81723	4.00318	N	7.86872	1.83454	-0.22050
H	-5.67117	-4.30084	4.52663	H	6.92830	-0.78130	2.44380
H	-8.70704	2.98731	1.97597	N	5.19633	-0.56580	-3.62304
H	-8.24513	-1.02353	-3.79371	P	5.22881	-1.01251	-0.14419
H	-7.44430	0.94085	2.94784	H	9.69574	2.87345	-0.51099
C	-5.11558	-1.86623	3.91670	H	4.78822	-0.24883	-5.68435
C	-4.75700	-4.29600	3.92098	N	5.21948	-1.66209	3.26562
C	-7.89039	3.24864	1.29265	C	6.16424	-0.56099	3.18495
H	-8.31002	3.35693	0.29382	C	4.28907	-0.20486	-4.70948
C	-6.28623	-1.86956	-2.33611	H	5.55915	1.22644	-2.56124
N	-5.31735	-1.63832	-1.48892	H	9.10095	2.82078	1.16291
N	-4.68292	-3.04242	3.17543	C	6.84366	2.86101	-0.08953
H	-4.76026	-0.96646	3.41904	H	4.91138	-2.01516	5.33899
H	-4.80045	-5.14446	3.24014	H	3.42275	-0.86292	-4.71354
C	-6.63560	1.61329	2.63234	C	4.37294	-1.65437	4.45470
H	-4.68396	-1.91395	4.92056	H	5.95602	2.57761	-0.64969
N	-6.88945	2.18506	1.31703	H	3.48909	-2.26834	4.29433
H	-3.89942	-4.41982	4.59134	H	5.67352	0.37829	2.90808
H	-7.38868	-3.52188	-3.97775	H	7.24120	3.79928	-0.48751
H	-7.05089	2.02469	-2.98136	H	6.63963	-0.43927	4.16349
H	-7.09188	0.48256	-2.10632	H	3.93222	0.81605	-4.54552
H	-7.46978	4.20903	1.60973	H	6.55195	3.01831	0.95694
N	-5.37982	-2.04305	1.22567	H	4.04232	-0.62913	4.64411

Table S25: Cartesian coordinates of the optimized geometry of isomer **4_I15** at the B3LYP(GD3BJ)/6-31G(d,p)/SMD(THF) level of theory.

Atom	X	Y	Z	Atom	X	Y	Z
C	-2.30520	3.25941	0.09485	C	6.42192	2.07608	1.04854
C	-2.33625	2.98739	-1.32408	C	5.80649	-1.24929	-2.72466
C	-1.25125	3.32398	-2.13021	H	5.83544	2.80778	-1.37451
C	-0.08289	3.95134	-1.53306	N	6.56859	0.99638	0.31803
C	-0.05336	4.21488	-0.16124	C	6.15616	0.86920	3.16145
C	-1.19120	3.86248	0.67381	H	6.56016	4.42634	-1.26472
C	-3.03604	1.71137	-1.50185	C	7.28114	-3.99018	3.13248
C	-0.80141	2.41217	-3.17243	N	6.46810	-2.84953	2.71707
C	1.09025	3.40486	-2.19649	H	6.65500	-3.15305	-1.17724
C	1.15064	3.94314	0.60734	N	6.07609	2.08417	2.36825
C	-0.69161	3.39726	1.95980	P	5.75166	-0.32487	-0.13861
C	-2.98469	2.14433	0.76113	H	7.49733	-3.95631	4.20590
C	2.27133	3.41904	-0.03519	H	6.16507	3.71610	3.72913
C	2.24070	3.14734	-1.45436	N	5.38963	-2.55514	-2.73076
C	3.06445	2.35494	0.58736	C	5.72358	-3.46094	-1.64604
C	3.01702	1.92138	-1.67399	C	5.46701	3.22907	3.03771
C	-2.65000	0.85659	-2.55116	H	5.19290	0.35024	3.20640
C	0.64435	2.46243	-3.21403	H	6.74190	-4.92159	2.91878
C	3.58086	1.49791	-0.43285	C	5.05535	-2.95937	3.05333
C	1.39794	1.29444	-3.42687	H	4.68720	-3.81387	-4.29110
C	-1.48603	1.19531	-3.34591	H	5.11903	3.95217	2.30265

C	0.75455	3.44629	1.91742	C	4.32655	-3.03563	-3.60841
C	-1.35799	2.33580	2.59898	H	4.57046	-1.99123	2.94787
C	-2.56257	-0.57397	-2.23856	H	3.90959	-2.21412	-4.18530
C	-2.55289	1.73682	2.03642	H	4.94160	-3.48943	-0.87998
C	-3.49800	1.25412	-0.23056	H	4.97315	-3.29600	4.09093
C	-0.56273	1.28801	3.22052	H	5.84960	-4.46607	-2.06240
C	-2.46531	0.29070	2.26832	H	4.59919	2.88112	3.60248
C	-3.69357	-0.22883	0.05618	H	4.53196	-3.67739	2.40865
C	2.62432	1.03713	-2.69746	H	3.52537	-3.45779	-2.99301
C	1.52483	2.43225	2.51421	H	-6.23541	-0.57332	4.33929
C	-0.70924	-0.01401	-3.56901	H	-7.92484	-0.29433	4.82664
C	-1.24770	0.02872	3.01924	C	-7.24660	-0.35826	3.97067
C	-2.91135	-0.63214	1.32966	H	-6.48320	1.91341	4.88585
C	-2.96139	-1.08176	-1.00892	H	-7.56965	-1.17733	3.32916
C	-2.14617	-2.07170	-0.36362	H	-8.19354	2.29644	4.57971
C	-1.37503	-1.10515	-2.88883	C	-7.23127	2.08307	4.10184
C	0.69273	0.03484	-3.60753	N	-7.31166	0.89954	3.24383
C	1.46679	-1.00471	-2.96366	H	-8.72372	-1.04203	0.80508
C	-2.11202	-1.79230	1.06018	H	-10.05445	0.82703	-0.20453
C	-0.96105	-2.09139	1.80771	H	-5.77232	-6.21088	-0.72481
C	0.21719	-2.63110	1.14075	H	-6.91776	2.95384	3.52911
C	-0.51519	-1.14622	2.80280	H	-5.96579	-4.11132	-2.13853
C	2.71679	1.91205	1.87578	C	-8.66934	-1.35185	-0.24595
C	0.82669	-2.07350	-2.32361	C	-9.66862	0.66095	-1.21599
C	0.83944	1.33502	3.18027	C	-4.95704	-5.81646	-0.10624
C	1.59630	0.12446	2.93974	H	-5.22742	-5.96527	0.93763
C	-0.62645	-2.12404	-2.28419	C	-6.85308	0.94269	1.94187
C	2.64660	-0.39134	-2.37651	N	-6.08467	-0.03468	1.53227
C	3.15232	-0.87113	-1.17483	N	-8.50600	-0.21425	-1.13606
C	-1.02455	-2.63620	-0.99429	H	-7.84237	-2.05010	-0.36209
C	0.18705	-2.89521	-0.22413	H	-9.39079	1.62266	-1.64480
C	0.93770	-1.09835	2.76320	C	-4.92065	-4.04962	-1.80576
C	1.39397	-2.01617	1.74422	H	-9.60326	-1.86690	-0.49843
C	1.33269	-2.55599	-1.05977	N	-4.78056	-4.39528	-0.39795
C	2.74615	0.46697	2.11693	H	-10.47477	0.22339	-1.81755
C	3.88465	0.03162	-0.15337	H	-9.17981	2.07346	2.18071
C	2.45197	-1.92098	-0.49036	H	-5.16230	-3.52810	3.78901
C	3.20337	-0.42316	1.15528	H	-5.91835	-2.37247	2.65971
C	2.48094	-1.64509	0.93559	H	-4.04995	-6.38990	-0.32550
H	8.94874	-0.21361	0.32988	N	-6.11543	-0.21553	-1.27563
H	9.79673	-1.78250	0.31096	C	-4.98493	-3.41488	0.54152
C	8.81510	-1.29990	0.37429	C	-7.30248	0.09813	-1.72020
H	9.46069	-0.55966	2.78002	H	-4.55799	-3.03924	-1.98246
H	8.22168	-1.60331	-0.48589	N	-5.62117	-2.32718	0.17708
H	9.78412	-2.30344	2.86369	C	-4.99422	-2.87469	2.92643
C	8.98991	-1.54964	2.80539	H	-4.32334	-4.75225	-2.39402
N	8.16909	-1.71288	1.61039	C	-8.55821	2.69760	1.54006
H	7.00078	1.14199	-3.93062	N	-7.31464	2.00844	1.21246
H	7.04255	-0.83192	-5.62770	H	-6.43763	2.27371	-4.05854
H	8.38904	4.50781	0.58019	N	-4.53943	-3.67680	1.80454
H	8.37214	-1.61444	3.69998	P	-5.50478	-0.71049	0.16243
H	7.60513	2.98939	-1.41934	H	-8.38470	3.65925	2.03805
C	5.94998	0.82816	-3.98975	H	-3.76415	-5.49901	2.58321
C	6.15937	-1.30766	-5.18498	N	-7.38033	0.84974	-2.87253
C	7.46471	4.33164	1.14390	C	-6.15894	1.46502	-3.37685
H	7.74256	4.01464	2.14772	C	-3.42831	-4.57701	2.09468
C	6.83474	-2.03988	1.66711	H	-4.25542	-2.11165	3.19564
N	5.90722	-1.63654	0.83847	H	-9.09705	2.89603	0.60879
N	5.81678	-0.62123	-3.94295	C	-6.57442	2.62577	0.12465
H	5.40129	1.27963	-3.16694	H	-8.92748	1.46653	-4.18618
H	6.39935	-2.35156	-4.98961	H	-2.89445	-4.82607	1.17898
C	6.67625	3.37682	-0.98046	C	-8.37447	0.56347	-3.90598
H	5.52818	1.18326	-4.93408	H	-7.14769	2.58676	-0.80631
N	6.68123	3.29629	0.47337	H	-9.07972	-0.19173	-3.56399
H	5.34154	-1.25919	-5.91229	H	-5.57402	1.87032	-2.55500
H	8.22068	-4.01099	2.58278	H	-5.61750	2.13294	-0.02306
H	6.46210	1.13098	4.17893	H	-5.53028	0.74609	-3.91733
H	6.90055	0.20691	2.73036	H	-2.73141	-4.06375	2.76200
H	6.91488	5.27684	1.20889	H	-6.38650	3.67936	0.36845
N	6.22814	-0.57083	-1.68258	H	-7.87131	0.17543	-4.80030

Table S26: : Cartesian coordinates of the optimized geometry of isomer **4_I16** at the B3LYP(GD3BJ)/6-31G(d,p)/SMD(THF) level of theory.

Atom	X	Y	Z	Atom	X	Y	Z
C	-3.05642	1.29527	0.74467	C	-6.08603	-2.36893	-0.75996
C	-2.74399	2.27672	-0.26091	C	-6.02264	2.70086	-0.37577
C	-1.69677	3.18983	-0.09439	H	-5.76901	-0.80844	-2.80540
C	-0.86526	3.09596	1.07829	N	-6.30784	-1.13571	-0.37195
C	-1.12495	2.10040	2.03206	C	-5.72694	-3.25545	1.48716
C	-2.23537	1.18219	1.85357	H	-6.23771	-2.00328	-4.03221
C	-3.01533	1.71013	-1.57240	C	-6.94889	-0.48829	5.37653
C	-0.84680	3.55088	-1.23133	N	-6.14084	-0.75208	4.18831
C	0.50166	3.38091	0.67147	H	-6.61283	2.49030	2.13264
C	-0.03033	1.36020	2.62306	N	-5.68214	-3.39813	0.04383
C	-1.82317	-0.13523	2.37758	P	-5.61813	0.08994	0.43589
C	-3.76772	0.08825	0.09304	H	-7.07827	-1.39210	5.98198
C	1.28410	1.63942	2.23458	H	-5.40359	-5.46304	-0.37461
C	1.54774	2.65365	1.23796	N	-5.62810	3.54921	0.62279
C	2.24256	0.56101	2.00785	C	-5.78586	3.18699	2.02114
C	2.66172	2.17484	0.41465	C	-4.86675	-4.50952	-0.43869
C	-2.22592	2.08399	-2.67040	H	-4.80602	-2.80436	1.87363
C	0.50755	3.68625	-0.75196	H	-6.45171	0.27066	5.99301
C	3.12002	0.93903	0.93511	C	-4.70662	-0.81583	4.43420
C	1.58392	3.21614	-1.53778	H	-5.36170	5.65794	0.59524
C	-1.11953	3.01569	-2.49533	H	-4.55755	-4.33347	-1.46674
C	-0.46355	-0.01293	2.83205	C	-4.80908	4.73416	0.38714
C	-2.22979	-1.27115	1.65731	H	-4.19602	-1.23602	3.57107
C	-1.82197	1.06398	-3.61262	H	-4.45267	4.75021	-0.64074
C	-3.11262	-1.21459	0.52535	H	-4.88219	2.71952	2.42664
C	-3.49056	0.36671	-1.40382	H	-4.53259	-1.45319	5.30626
C	-1.27130	-2.35411	1.45330	H	-6.00361	4.09532	2.59213
C	-2.65868	-2.16098	-0.42696	H	-3.96362	-4.57477	0.17486
C	-3.08153	-0.61793	-2.29180	H	-4.28075	0.17610	4.63355
C	2.69908	2.51892	-0.96230	H	-3.93716	4.69586	1.04655
C	0.48032	-1.05032	2.62482	H	8.76311	0.32987	0.85612
C	-0.01877	2.54755	-3.31625	H	9.64015	-0.91413	1.78358
C	-1.54103	-2.91504	0.14814	C	8.64690	-0.54160	1.51004
C	-2.70372	-1.95861	-1.83162	H	9.11292	1.46833	3.07966
C	-2.23461	-0.26271	-3.41822	H	8.12660	-1.31731	0.95259
C	-1.30981	-1.35269	-3.63389	H	9.44881	0.11026	4.17232
C	-0.45640	1.34246	-4.01371	C	8.65239	0.65171	3.64829
C	1.29766	2.64162	-2.85326	N	7.92111	-0.21250	2.72701
C	2.22274	1.55208	-3.07132	H	7.28458	-1.23400	-3.42560
C	-1.59063	-2.38341	-2.63310	H	7.61565	-3.78227	-3.56152
C	-0.51144	-3.11591	-2.08974	H	7.93849	4.39047	-1.80453
C	0.84069	-2.80665	-2.48772	H	7.97357	1.08296	4.38250
C	-0.49846	-3.37271	-0.65657	H	7.50502	1.97331	-2.60327
C	1.83779	-0.76366	2.24499	C	6.25471	-1.61326	-3.38319
C	1.80717	0.39798	-3.75186	C	6.68664	-4.00168	-3.02175
C	0.04487	-2.24269	1.91330	C	6.99621	4.44819	-1.24683
C	1.13609	-2.70385	1.08140	H	7.22412	4.78061	-0.23545
C	0.44003	0.29114	-4.22444	C	6.58638	-0.50258	2.89004
C	3.07430	1.45369	-1.89751	N	5.71145	-0.68844	1.93597
C	3.48905	0.20666	-1.45112	N	6.13625	-2.75742	-2.49039
C	0.00452	-1.08881	-4.03299	H	5.60135	-0.81083	-3.04895
C	1.10840	-1.83187	-3.45539	H	6.92238	-4.69220	-2.21367
C	0.87026	-3.26119	-0.17852	C	6.49346	2.37911	-2.46876
C	1.69664	-2.90366	-1.30343	H	5.95242	-1.92531	-4.38703
C	2.21446	-0.90235	-3.26861	N	6.37166	3.12755	-1.22541
C	2.24660	-1.78471	1.25924	H	5.99212	-4.48778	-3.71562
C	3.77148	-0.10357	0.03901	H	7.92356	-1.51491	4.84965
C	3.01051	-0.97246	-2.11507	H	5.96549	3.43907	3.06522
C	3.06061	-1.46761	0.18602	H	6.65882	1.94357	2.41368
C	2.74288	-1.99379	-1.11573	H	6.35437	5.19209	-1.73101
H	-8.72095	-0.65298	0.71504	N	6.24246	-1.33590	-0.68744
H	-9.65011	0.19734	1.97663	C	6.13326	2.43893	-0.06319
C	-8.64215	-0.03198	1.61434	C	5.97083	-2.53450	-1.14560
H	-9.05344	-2.44239	2.49270	H	5.78194	1.55688	-2.47956
H	-8.15016	0.89931	1.34146	N	6.34265	1.14333	-0.05880
H	-9.42541	-1.48775	3.94247	C	5.81493	2.62436	2.35130
C	-8.61494	-1.82458	3.28543	H	6.27008	3.05398	-3.29985
N	-7.90718	-0.70379	2.67495	C	6.94918	-1.22062	5.23598
H	-7.33423	2.00432	-2.89433	N	6.15360	-0.57743	4.19267

H	-7.75647	4.48275	-2.34123	H	6.61965	-3.03976	1.30841
H	-7.88927	-3.74364	-2.98826	N	5.74589	3.18480	1.01409
H	-7.92464	-2.44090	3.85940	P	5.62043	-0.24810	0.35950
H	-7.48636	-1.19563	-3.04901	H	7.09237	-0.55670	6.09548
C	-6.31723	2.40084	-2.77344	H	5.52226	5.28766	1.22952
C	-6.81777	4.58889	-1.78408	N	5.56804	-3.61380	-0.40531
C	-6.94124	-3.95083	-2.47759	C	5.77822	-3.66917	1.03109
H	-7.15555	-4.56890	-1.60724	C	4.95763	4.40853	0.89797
C	-6.58027	-0.44214	2.92391	H	4.89656	2.08465	2.60793
N	-5.71823	0.04924	2.07164	H	6.43350	-2.12511	5.58180
N	-6.20932	3.27176	-1.61169	C	4.72111	-0.55697	4.45381
H	-5.62643	1.56613	-2.67868	H	5.19939	-5.62676	-0.97612
H	-7.05006	5.03353	-0.81791	H	4.62856	4.54999	-0.12917
C	-6.46840	-1.60769	-3.03900	C	4.69129	-4.65633	-0.92869
H	-6.05646	2.98027	-3.66370	H	4.22362	0.12512	3.76843
N	-6.33103	-2.68718	-2.07180	H	4.33128	-4.38802	-1.91914
H	-6.16191	5.26723	-2.34055	H	4.89531	-3.33503	1.58602
H	-7.92923	-0.10574	5.09721	H	4.56191	-0.22100	5.48276
H	-5.85782	-4.24541	1.93322	H	6.00122	-4.70492	1.30842
H	-6.57312	-2.63293	1.76176	H	4.06490	4.31033	1.52223
H	-6.29610	-4.51080	-3.16325	H	4.26780	-1.54961	4.33382
N	-6.27010	1.42017	-0.24671	H	3.82431	-4.75182	-0.26759

Table S27: Cartesian coordinates of the optimized geometry of isomer **4_I17** at the B3LYP(GD3BJ)/6-31G(d,p)/SMD(THF) level of theory.

Atom	X	Y	Z	Atom	X	Y	Z
C	3.63036	-0.21210	-0.13298	C	-5.08806	-3.04980	1.10067
C	3.20767	0.71827	-1.29621	C	-5.60690	-0.04236	-2.96224
C	2.34698	0.32141	-2.30377	H	-3.91456	-3.53838	-1.15508
C	1.64137	-0.96854	-2.25586	N	-5.59845	-2.23126	0.20921
C	1.77816	-1.71813	-1.06760	C	-5.81923	-1.75357	3.04260
C	2.67648	-1.39107	-0.01637	H	-3.63014	-5.26614	-0.85319
C	3.16608	2.08350	-0.83661	C	-8.93533	2.03913	2.33834
C	1.47092	1.29175	-2.94524	N	-7.64560	1.37007	2.17778
C	0.32746	-0.74977	-2.79864	H	-7.29463	1.40264	-1.64332
C	0.59648	-2.38094	-0.49457	N	-5.02489	-2.81172	2.44538
C	2.01888	-1.71351	1.21504	P	-5.44963	-0.69097	-0.28413
C	3.46422	0.74082	1.06965	H	-9.28789	1.98177	3.37385
C	-0.65714	-2.19157	-1.07669	H	-4.42973	-4.09204	4.03518
C	-0.81096	-1.38143	-2.25268	N	-5.78884	1.30764	-3.09023
C	-1.83206	-1.96046	-0.22182	C	-6.57000	2.05674	-2.12135
C	-2.05524	-0.63612	-2.08108	C	-4.00268	-3.38188	3.31753
C	2.35870	3.04239	-1.46305	H	-5.26473	-0.80997	3.09432
C	0.22920	0.62786	-3.26059	H	-8.83497	3.09728	2.06627
C	-2.71816	-1.07061	-0.88768	C	-6.50530	2.14544	2.64566
C	-0.99237	1.31699	-3.12156	H	-5.65701	2.54911	-4.81175
C	1.49217	2.63764	-2.54606	H	-3.23462	-3.87526	2.72601
C	0.75938	-2.39979	0.93224	C	-5.02301	2.12948	-4.02189
C	2.14422	-0.92609	2.38122	H	-5.63054	1.50615	2.74261
C	1.65624	4.03582	-0.65566	H	-4.22166	1.54515	-4.46931
C	2.84091	0.36073	2.24702	H	-5.93839	2.50061	-1.34432
C	3.31851	2.09374	0.60701	H	-6.75351	2.56710	3.62392
C	0.97889	-0.69201	3.19076	H	-7.10154	2.85661	-2.64717
C	2.13719	1.35095	3.04933	H	-3.52649	-2.56938	3.87433
C	2.67570	3.07340	1.38001	H	-6.25516	2.96676	1.96137
C	-2.16294	0.67912	-2.58263	H	-4.56816	2.95575	-3.46700
C	-0.37250	-2.17797	1.74671	H	8.64995	-0.97493	-0.65005
C	0.23891	3.35655	-2.40453	H	9.35788	-2.50882	-0.08149
C	0.98154	0.70610	3.62792	C	8.46322	-1.87668	-0.05663
C	2.06187	2.68562	2.62708	H	9.78341	-0.25709	1.48829
C	1.81222	4.05188	0.73175	H	7.64087	-2.42089	-0.51638
C	0.66229	4.26777	1.59164	H	10.01140	-1.79833	2.33891
C	0.34213	4.22807	-1.23885	C	9.27813	-1.02198	2.08999
C	-0.97122	2.71279	-2.67792	N	8.15448	-1.55651	1.32821
C	-2.11799	2.92732	-1.82582	H	5.68959	-1.56884	-4.50632
C	0.81797	3.42199	2.76788	H	5.29514	-4.09007	-4.92937
C	-0.30022	2.79480	3.32476	H	8.22063	3.39416	-2.77457
C	-1.61434	2.99425	2.74387	H	8.92432	-0.55931	3.01017
C	-0.21638	1.41068	3.77870	H	6.96438	1.24865	-3.53887
C	-1.68557	-2.02149	1.18087	C	4.65366	-1.72112	-4.17425
C	-2.02633	3.77866	-0.71573	C	4.54513	-4.17287	-4.13371

C	-0.25446	-1.31530	2.91434	C	7.49094	3.61605	-1.98674
C	-1.48297	-0.56835	3.03998	H	8.03757	3.76873	-1.05772
C	-0.76873	4.44008	-0.41621	C	6.87087	-1.57819	1.82162
C	-2.83792	1.66647	-1.72954	N	5.77233	-1.44010	1.12783
C	-3.46695	1.31584	-0.54866	N	4.51652	-2.92775	-3.37221
C	-0.60550	4.45575	1.03154	H	4.33972	-0.85369	-3.59912
C	-1.76413	3.80611	1.61801	H	4.82363	-5.00627	-3.49066
C	-1.48169	0.76235	3.48714	C	6.17054	1.88275	-3.12268
C	-2.33653	1.72927	2.83796	H	4.01149	-1.81540	-5.05455
C	-2.64379	3.39331	0.53116	N	6.56561	2.49234	-1.86040
C	-2.36739	-0.97686	1.95902	H	3.57551	-4.38728	-4.59651
C	-3.65464	-0.15301	-0.11059	H	8.34351	-3.13391	3.25103
C	-3.31132	2.16004	0.60590	H	7.32923	2.40556	2.37852
C	-3.22192	-0.06281	1.37165	H	7.39348	0.86105	1.50863
C	-3.15764	1.31801	1.77910	H	6.96956	4.54256	-2.25059
H	-8.27686	-2.24442	-0.12948	N	5.45063	-1.82957	-1.58406
H	-9.72498	-1.25171	-0.43797	C	6.45889	1.74436	-0.71447
C	-8.65454	-1.21887	-0.20706	C	4.78595	-2.87099	-2.02856
H	-9.28940	-2.01716	2.18201	H	5.27887	1.27558	-2.97914
H	-8.13632	-0.72769	-1.02804	N	6.35404	0.44273	-0.82699
H	-10.35814	-0.61200	1.99760	C	6.78049	1.73486	1.71093
C	-9.31519	-0.92203	2.13288	H	5.94620	2.68209	-3.83446
N	-8.46721	-0.47856	1.03119	C	7.58867	-2.70511	3.90806
H	-5.51018	-2.80736	-3.93424	N	6.76521	-1.73770	3.18499
H	-6.28289	-1.23676	-5.82992	H	5.95984	-3.87343	0.07430
H	-5.41218	-6.19967	0.81232	N	6.51007	2.43237	0.46590
H	-8.94640	-0.52554	3.07766	P	5.41203	-0.78601	-0.33322
H	-5.25708	-4.69661	-1.30781	H	8.08638	-2.24091	4.76638
C	-4.70526	-2.06144	-3.97823	H	6.91387	4.49895	0.77883
C	-5.74717	-0.37089	-5.42282	N	4.38268	-3.95682	-1.29700
C	-4.82835	-5.50209	1.42450	C	4.92517	-4.20586	0.02645
H	-5.38811	-5.30705	2.33772	C	6.07251	3.81797	0.60542
C	-7.44001	0.41962	1.20382	H	5.85722	1.41822	2.20943
N	-6.30528	0.43312	0.55461	H	6.95608	-3.52251	4.27650
N	-5.22828	-0.70835	-4.10020	C	5.48674	-1.44332	3.81805
H	-4.10093	-2.14295	-3.07741	H	3.29916	-5.70989	-1.80875
H	-6.44905	0.45900	-5.35834	H	5.53119	4.13192	-0.28563
C	-4.34801	-4.44824	-0.74438	C	3.12786	-4.65390	-1.56947
H	-4.07867	-2.26941	-4.85035	H	5.05780	-0.53912	3.39119
N	-4.62246	-4.26591	0.67445	H	2.59940	-4.17750	-2.39189
H	-4.94326	-0.10552	-6.11810	H	4.35581	-3.68618	0.80399
H	-9.68309	1.59320	1.68475	H	5.65970	-1.29110	4.88728
H	-6.09938	-2.05321	4.05688	H	4.89077	-5.28370	0.21409
H	-6.72195	-1.60636	2.45770	H	5.38992	3.88501	1.45640
H	-3.87855	-5.98246	1.68342	H	4.76196	-2.25719	3.68833
N	-5.80099	-0.74862	-1.87348	H	2.48964	-4.59551	-0.68218

Table S28: Cartesian coordinates of the optimized geometry of isomer **4_I18** at the B3LYP(GD3BJ)/6-31G(d,p)/SMD(THF) level of theory.

Atom	X	Y	Z	Atom	X	Y	Z
C	2.79614	0.47167	-2.06533	N	-6.18953	-0.55773	1.34090
C	2.85202	1.64105	-1.20253	N	-8.50230	-1.12923	1.10945
C	1.90822	2.66511	-1.34480	H	-8.88100	0.04281	-0.61926
C	0.84289	2.53869	-2.33408	C	-8.14169	-2.45161	3.63761
C	0.78537	1.41621	-3.15759	H	-10.30262	-2.17920	1.53906
C	1.79213	0.36562	-3.03589	C	-8.95137	0.09953	0.47269
C	3.35499	1.24582	0.08618	H	-7.73800	-2.17777	4.62040
C	1.34515	3.27529	-0.16128	H	-9.01295	-1.82756	3.44591
C	-0.37161	3.07473	-1.74205	H	-8.35281	0.94404	0.80791
C	-0.49104	0.77503	-3.42728	N	-6.16701	1.87947	0.06905
C	1.12884	-0.90578	-3.22749	H	-5.29380	1.38027	3.20042
C	3.25716	-0.67136	-1.32023	H	-9.99552	0.27772	0.75199
C	-1.65685	1.29157	-2.85070	H	-4.99144	3.14053	-1.77018
C	-1.59682	2.45084	-2.00131	C	-6.06101	2.12807	2.97356
C	-2.66466	0.39231	-2.27510	H	-6.93052	1.61619	2.56917
C	-2.56835	2.24066	-0.92105	C	-5.81145	2.98292	0.68352
C	2.78700	1.80752	1.21880	N	-5.58420	3.12010	2.02566
C	-0.05839	3.54860	-0.40735	H	-6.34439	2.63983	3.89908
C	-3.27681	1.02390	-1.15200	C	-5.59561	4.00794	-1.51324
C	-0.99854	3.34049	0.62071	H	-6.58256	3.90600	-1.98388
C	1.78356	2.85006	1.10044	N	-5.69460	4.12710	-0.06561

C	-0.27689	-0.65827	-3.48936	H	-5.11159	4.90909	-1.90064
C	1.53076	-2.00913	-2.46168	H	-3.94151	3.56733	3.23038
C	2.47543	1.00092	2.41072	C	-4.64766	4.09259	2.58014
C	2.59579	-1.87749	-1.48318	H	-4.07980	4.56642	1.78220
C	3.86059	-0.21455	0.03091	C	-6.14969	5.43105	0.40964
C	0.53166	-2.90024	-1.90881	H	-5.16022	4.86041	3.17155
C	2.27992	-2.74669	-0.33729	H	-6.56495	5.34982	1.41283
C	3.27424	-1.02401	1.18071	H	-6.94026	5.80020	-0.25472
C	-2.28221	2.74344	0.36380	H	-5.33672	6.16532	0.41855
C	-1.25079	-1.51030	-2.93267	H	5.60506	4.09259	-3.48004
C	0.82124	2.66036	2.16607	H	4.26753	3.88285	-2.32814
C	0.99451	-3.34089	-0.59238	C	4.99984	3.35329	-2.94262
C	2.56228	-2.23657	0.94606	H	4.45637	2.74097	-3.66048
C	2.66611	-0.39162	2.30771	H	7.00091	2.51056	-4.31322
C	1.65450	-1.29046	2.87944	H	6.46263	4.13849	-0.95547
C	1.24734	1.50918	2.96238	H	5.85604	1.81343	-5.48165
C	-0.53517	2.89812	1.93668	C	6.59110	1.58062	-4.70368
C	-1.53339	2.00460	2.48711	C	6.17903	3.09413	-0.79649
C	1.59129	-2.44694	2.02679	H	5.35009	3.05760	-0.08043
C	0.36782	-3.07394	1.77062	N	5.81448	2.50829	-2.07449
C	-0.84707	-2.54073	2.36415	H	7.41237	1.01680	-5.16224
C	0.05448	-3.54856	0.43603	N	6.00433	0.78488	-3.62749
C	-2.47656	-1.00001	-2.37797	H	7.02924	2.55815	-0.38436
C	-1.13241	0.90281	3.25532	C	6.03649	1.18045	-2.31272
C	-0.82494	-2.66164	-2.13645	H	5.80635	3.43416	3.66601
C	-1.78635	-2.85014	-1.06939	H	5.10023	2.55992	2.28540
C	0.27327	0.65682	3.51804	H	5.50922	-0.75208	-4.93466
C	-2.59526	1.86989	1.50664	C	5.88889	-0.63794	-3.91552
C	-3.25980	0.66660	1.34832	C	5.75501	2.47119	3.14951
C	0.48790	-0.77611	3.45629	N	6.30980	0.28318	-1.39827
C	-0.78826	-1.41793	3.18663	H	9.64926	2.41143	-0.02977
C	-1.34943	-3.27705	0.19253	H	8.82831	3.18714	1.33781
C	-1.91535	-2.67101	1.37777	H	6.85533	-1.15313	-3.83630
C	-1.79567	-0.36817	3.06437	C	9.35487	2.28861	1.01934
C	-2.78607	-1.80439	-1.18497	H	5.32155	1.72789	3.83097
C	-3.86508	0.21243	-0.00115	N	7.09889	2.10681	2.72246
C	-2.80237	-0.47585	2.09661	P	5.73748	-0.30991	0.00172
C	-3.36084	-1.24905	-0.05457	H	5.18927	-1.10168	-3.22265
C	-2.86195	-1.64801	1.23648	H	8.40633	3.32581	3.86381
H	-5.59238	-3.98726	-3.57041	C	7.23569	1.18669	1.70783
H	-4.28297	-3.77741	-2.38636	N	6.18956	0.48225	1.36568
C	-5.00554	-3.24732	-3.01377	N	8.48301	1.12435	1.13185
H	-4.44918	-2.62337	-3.70999	H	8.92135	0.08651	-0.66421
H	-6.94484	-2.34168	-4.42790	C	8.12448	2.27338	3.75031
H	-6.56480	-4.07556	-1.12039	H	10.26709	2.17762	1.61765
H	-5.74108	-1.61873	-5.51942	C	8.95643	-0.05332	0.42178
C	-6.51306	-1.40356	-4.77268	H	7.74059	1.91345	4.71324
C	-6.26205	-3.04497	-0.91274	H	9.01280	1.69400	3.50466
H	-5.45160	-3.05697	-0.17542	H	8.34904	-0.92067	0.67150
N	-5.84880	-2.42030	-2.15617	N	6.14960	-1.88678	-0.04594
H	-7.30782	-0.82291	-5.25581	H	5.50242	-1.46661	3.07361
N	-5.97933	-0.63938	-3.64819	H	9.99191	-0.25351	0.71940
H	-7.11071	-2.50505	-0.50326	H	4.91669	-3.05993	-1.87621
C	-6.03951	-1.08067	-2.35202	C	6.23623	-2.24669	2.84586
H	-5.85298	-3.65337	3.45887	H	7.12964	-1.77458	2.44296
H	-5.13481	-2.71099	2.13204	C	5.83378	-3.01299	0.54958
H	-5.41057	0.93400	-4.88102	N	5.71583	-3.21674	1.89910
C	-5.84625	0.79069	-3.88812	H	6.49995	-2.77421	3.76793
C	-5.78237	-2.66078	3.00462	C	5.49542	-3.95701	-1.67202
N	-6.31952	-0.20985	-1.41086	H	6.46163	-3.88003	-2.18866
H	-9.70141	-2.31485	-0.12555	N	5.66343	-4.12465	-0.23609
H	-8.88626	-3.19498	1.18005	H	4.95599	-4.82521	-2.06126
H	-6.81642	1.30340	-3.84874	H	4.06917	-3.58853	3.13139
C	-9.39734	-2.26723	0.92682	C	4.73718	-4.14246	2.46441
H	-5.32687	-1.97363	3.72975	H	4.13123	-4.58268	1.67576
N	-7.11992	-2.23821	2.61439	C	6.09412	-5.45768	0.17539
P	-5.74267	0.30662	0.01664	H	5.22216	-4.93954	3.03984
H	-5.18606	1.22841	-3.14211	H	6.56580	-5.42000	1.15601
H	-8.45608	-3.50029	3.67480	H	6.83143	-5.83478	-0.54332
C	-7.24781	-1.25637	1.65841	H	5.25564	-6.16202	0.20922

4 References

1 S. Stoll and A. Schweiger, *J. Mag. Res.*, 2006, **178**, 42.

2 F. Buß, M. B. Röthel, J. A. Werra, P. Rotering, L. F. B. Wilm, C. G. Daniliuc, P. Löwe and F. Dielmann, *Chem. Eur. J.*, 2022, **28**, e202104021.

3 *Deutschland Pat.*, DE 10 2006 010 034 A1 2007.09.06, 2006.

4 P. Jutzi, C. Müller, A. Stammler and H.-G. Stammler, *Organometallics*, 2000, **19**, 1442.

5 I. P. Romanova, V. F. Mironov, O. A. Larionova, V. I. Morozov, V. V. Zverev, O. G. Sinyashin, *Russ. Chem. Bull.*, 2008, **57**, 209.

6 J. R. Aranzaes, M.-C. Daniel and D. Astruc, *Can. J. Chem.*, 2006, **84**, 288.

7 a) R. Kinjo, B. Donnadieu and G. Bertrand, *Angew. Chem. Int. Ed.*, 2010, **49**, 5930; b) O. Back, B. Donnadieu, M. von Hopffgarten, S. Klein, R. Tonner, G. Frenking and G. Bertrand, *Chem. Sci.*, 2011, **2**, 858;

8 G. M. Sheldrick, *Acta Cryst.*, 2015, **A71**, 3.

9 O. V. Dolomanov, L. J. Bourhis, R. J. Gildea, J. A. K. Howard and H. Puschmann, *J. Appl. Crystallogr.*, 2009, **42**, 339.

10 G. M. Sheldrick, *Acta Cryst.*, 2015, **C71**, 3.

11 Gaussian 16, Revision C.01, Inc., Wallingford CT, 2016.

12 S. Grimme, C. Bannwarth and S. Ehlert, grimme-lab/xtb: xtb version 6.3.2, Zenodo, 2020.

13 M. D. Hanwell, D. E. Curtis, D. C. Lonie, T. Vandermeersch, E. Zurek and G. R. Hutchison, *J. Cheminform.*, 2012, **4**, 17.

14 C. Lee, W. Yang and R. G. Parr, *Phys. Rev. B Condens. Matter*, 1988, **37**, 785.

15 A. D. Becke, *J. Chem. Phys.*, 1993, **98**, 5648.

16 M. S. Gordon, J. S. Binkley, J. A. Pople, W. J. Pietro and W. J. Hehre, *J. Am. Chem. Soc.*, 1982, **104**, 2797.

17 M. M. Francl, W. J. Pietro, W. J. Hehre, J. S. Binkley, M. S. Gordon, D. J. DeFrees and J. A. Pople, *J. Chem. Phys.*, 1982, **77**, 3654.

18 P. C. Hariharan and J. A. Pople, *Theoret. Chim. Acta*, 1973, **28**, 213.

19 W. J. Hehre, R. Ditchfield and J. A. Pople, *J. Chem. Phys.*, 1972, **56**, 2257.

20 R. Ditchfield, W. J. Hehre and J. A. Pople, *J. Chem. Phys.*, 1971, **54**, 724.

21 A. V. Marenich, C. J. Cramer and D. G. Truhlar, *J. Phys. Chem. B*, 2009, **113**, 6378.

22 S. Grimme, S. Ehrlich and L. Goerigk, *J. Comput. Chem.*, 2011, **32**, 1456.

23 Zhurko G. A., Chemcraft - graphical program for visualization of quantum chemistry computations, Ivanovo, Russia, 2005. https://chemcraftprog.com.

24 W. H. Hersh and T.-Y. Chan, *Beilstein J. Org. Chem.*, 2023, **19**, 36.

25 Y. Zhao and D. G. Truhlar, *Theor. Chem. Acc.*, 2008, **120**, 215.

26 a) G. W. Spitznagel, T. Clark, P. von Ragué Schleyer and W. J. Hehre, *J. Comput. Chem.*, 1987, **8**, 1109; b) A. D. McLean and G. S. Chandler, *J. Chem. Phys.*, 1980, **72**, 5639; c) R. Krishnan, J. S. Binkley, R. Seeger and J. A. Pople, *J. Chem. Phys.*, 1980, **72**, 650; d) T. Clark, J. Chandrasekhar, G. W. Spitznagel and P. V. R. Schleyer, *J. Comput. Chem.*, 1983, **4**, 294;

27 F. London, *J. Phys. Radium*, 1937, **8**, 397.

28 R. McWeeny, *Phys. Rev.*, 1962, **126**, 1028.

29 R. Ditchfield, *Mol. Phys.*, 1974, **27**, 789.

30 K. Wolinski, J. F. Hinton and P. Pulay, *J. Am. Chem. Soc.*, 1990, **112**, 8251.

31 J. R. Cheeseman, G. W. Trucks, T. A. Keith and M. J. Frisch, *J. Chem. Phys.*, 1996, **104**, 5497.

32 K. Balasubramanian, *J. Phys. Chem.*, 1993, **97**, 6990.

33 D. B. Chesnut, *J. Phys. Chem. A*, 2005, **109**, 11962.

34 K. Abdur-Rashid, T. P. Fong, B. Greaves, D. G. Gusev, J. G. Hinman, S. E. Landau, A. J. Lough and R. H. Morris, *J. Am. Chem. Soc.*, 2000, **122**, 9155.

35 J. R. van Wazer, C. F. Callis, J. N. Shoolery and R. C. Jones, *J. Am. Chem. Soc.*, 1956, **78**, 5715.

36 N. Zumbulyadis and B. P. Dailey, *Mol. Phys.*, 1974, **27**, 633.

37 N. Donoghue and M. J. Gallagher, *Phosphorus, Sulfur, Silicon Relat. Elem.*, 1997, **123**, 169.

38 A. J. Naaktgeboren, R. J. M. Nolte and W. Drenth, *J. Am. Chem. Soc.*, 1980, **102**, 3350.

39 F. Buß, P. Mehlmann, C. Mück-Lichtenfeld, K. Bergander and F. Dielmann, *J. Am. Chem. Soc.*, 2016, **138**, 1840.

40 T.-F. Leung, D. Jiang, M.-C. Wu, D. Xiao, W.-M. Ching, G. P. A. Yap, T. Yang, L. Zhao, T.-G. Ong and G. Frenking, *Nat. Chem.*, 2021, **13**, 89.

41 D. B. Chesnut, *Chem. Phys. Lett.*, 2003, **380**, 251.
